# Oncopeptide MBOP Encoded by *LINC01234* Promotes Colorectal Cancer through MAPK Signaling Pathway

**DOI:** 10.3390/cancers14092338

**Published:** 2022-05-09

**Authors:** Chunyuan Tang, Ying Zhou, Wen Sun, Haihong Hu, Yuxi Liu, Lu Chen, Fengting Ou, Su Zeng, Nengming Lin, Lushan Yu

**Affiliations:** 1 Institute of Drug Metabolism and Pharmaceutical Analysis, College of Pharmaceutical Sciences, Zhejiang University, Hangzhou 310058, China; tangchunyuan@zju.edu.cn (C.T.); 12119004@zju.edu.cn (Y.Z.); sunw127@zju.edu.cn (W.S.); huhaihong@zju.edu.cn (H.H.); liuyuxi@zju.edu.cn (Y.L.); 0919361@zju.edu.cn (F.O.); zengsu@zju.edu.cn (S.Z.); 2 Key Laboratory of Clinical Cancer Pharmacology and Toxicology Research of Zhejiang Province, Department of Clinical Pharmacy, Affiliated Hangzhou First People’s Hospital, Zhejiang University School of Medicine, Hangzhou 310003, China; chen_lu@zju.edu.cn (L.C.); lnm1013@zju.edu.cn (N.L.); 3 Westlake Laboratory of Life Sciences and Biomedicine of Zhejiang Province, Westlake University, Hangzhou 310024, China

**Keywords:** peptide, MBOP, LncRNA, colorectal cancer, MAPK signaling pathway

## Abstract

**Simple Summary:**

Colorectal cancer (CRC) claimed more than 900,000 lives globally in 2020. Therefore, its pathogenetic landscape calls for in-depth investigation. Recently, more attention has been paid to oncogenesis driven by noncoding RNAs and even peptides encoded by noncoding RNAs. Here, we report a *LINC01234*-encoded peptide named MEK1-binding oncopeptide (MBOP), which exists endogenously and is highly expressed in CRC. Through in vivo and in vitro migration and proliferation assays, we demonstrated the oncogenic role of MBOP. In addition, we performed immunoprecipitation assays to identify the interacting proteins and pathways, and MBOP was found to interact with MEK1 and activate the MEK1/pERK/MMP2/MMP9 signaling pathway. Moreover, the ubiquitin–protease-system-mediated degradation of MBOP was elucidated, where the E3 ubiquitin-protein transferase MAEA and RMND5A play vital roles. In conclusion, oncopeptide MBOP plays a substantial role in the tumorigenesis of CRC, and it could be a candidate prognostic biomarker for clinical treatment.

**Abstract:**

Colorectal cancer (CRC) ranks third in incidence rate and second in mortality rate of malignancy worldwide, and the diagnosis and therapeutics of it remain to be further studied. With the emergence of noncoding RNAs (ncRNAs) and potential peptides derived from ncRNAs across various biological processes, we here aimed to identify a ncRNA-derived peptide possible for revealing the oncogenesis of CRC. Through combined predictive analysis of the coding potential of a batch of long noncoding RNAs (lncRNAs), the existence of an 85 amino-acid-peptide, named MEK1-binding oncopeptide (MBOP) and encoded from *LINC01234* was confirmed. Mass spectrometry and Western blot assays indicated the overexpression of MBOP in CRC tissues and cell lines compared to adjacent noncancerous tissues and the normal colonic epithelial cell line. In vivo and in vitro migration and proliferation assays defined MBOP as an oncogenic peptide. Immunoprecipitation trials showed that MEK1 was the key interacting protein of MBOP, and MBOP promoted the MEK1/pERK/MMP2/MMP9 axis in CRC. Two E3-ligase enzymes MAEA and RMND5A mediated the ubiquitin–protease-system-related degradation of MBOP. This study indicates that MBOP might be a candidate prognostic indicator and a potential target for clinical therapy of CRC.

## 1. Introduction

Colorectal cancer (CRC) ranks third in incidence rate and second in mortality rate of malignancy worldwide, with more than 900,000 deaths in 2020 [1]. The pathogenetic landscape of CRC remains ambiguous, but plausible hypotheses include hereditary factors, Western-style diets, inflammation, gene mutation, and the gut microbiome. Accumulated research and clinical trials on CRC have identified molecularly stratified treatment options, which greatly depend on biomarkers expressed in individual patients [2]. Some biomarkers have created substantial breakthroughs [3] in diagnosis and therapeutics, whereas others have not. Yet, the heterogeneous and complex interactions of the present biomarkers in patients with CRC still call for in-depth research on new generalized targets.

The mitogen-activated protein kinases (MAPKs) consist of three main subfamilies: extracellular signal-related kinase (ERK), Jun N-terminal kinase (JNK), and p38 proteins [4], which play vital roles in cell differentiation, proliferation, apoptosis, metabolism, and inflammation across various cell types. Hyperactivation of MAPK signaling pathways is ubiquitous in and has a strong influence on CRC; therefore, many therapeutics focus on these targets and have gained positive feedback [5].

Noncoding RNAs are now regarded as ‘functional peptides templates’ rather than merely ‘junk transcripts’. During the last decade, we have witnessed several identifications of noncoding RNA-derived peptides, including a small regulatory peptide of STAT3 (ASRPS) [6], small integral membrane protein 30 (SMIM30) [7], RNA-binding regulatory peptide (RBRP) [8], and circPPP1R12A-73aa [9], which strongly reshaped our perspectives on noncoding RNAs and broadened therapeutic strategies for various diseases. The emerging focus on this field has led to a parallel increase in accessible prediction or validated databases of noncoding-RNA-derived peptides, such as CPC2 [10], CPAT [11], RegRNA 2.0 [12], GWIPS-viz [13], POSTAR3 [14], and even GEO datasets, for example, GSE139407 [15].

From this perspective, we used available database resources to screen a batch of long noncoding RNAs (lncRNAs) that were prone to encode peptides. We selected one oncopeptide which was encoded by *LINC01234*. This oncopeptide was overexpressed in CRC and was named MEK1-binding oncopeptide (MBOP) for its interaction with MEK1. Furthermore, the roles of MBOP in cancer signaling pathways were studied and it was found that MBOP activated the MEK1/pERK/MMP2/MMP9 signaling pathway. We also investigated the degradation of MBOP and found it to be ubiquitin–protease-system-related. These findings of the present study will help in paving the path for future diagnosis and treatment of CRC.

## 2. Materials and Methods

### 2.1. Cell Culture and Treatments

The human colorectal cancer (CRC) cell lines HCT116, HCT15, and HT29 were gifts from the Institute of Modern Chinese Medicine, College of Pharmaceutical Sciences, Zhejiang University. CRC cell lines SW620 and RKO, embryonic kidney cell line 293T, and cervical cancer cell line Hela were purchased from the National Collection of Authenticated Cell Cultures of the Chinese Academy of Sciences (Shanghai, China, with verified STR authentication). Human gastric cancer cell lines MKN-45 and HGC-27 were purchased from the National Infrastructure of Cell Line Resource (Beijing, China, with verified STR authentication). Normal colonic epithelial cell line FHC and CRC cell line SW48 were purchased from the American Type Culture Collection cell bank (ATCC, Manassas, VA, USA). CRC cell lines HCT116, HCT15, HT29, SW620, RKO, and FHC, and gastric cancer cells MKN-45 and HGC-27 were maintained in medium 1640 (Corning, New York, NY, USA), 293T and SW48 were cultured in medium DMEM (Corning, New York, NY, USA), Hela was cultured in medium MEM (Corning, New York, NY, USA), and all were cultured with 10% (*v*/*v*) fetal bovine serum (Gibco, Grand Island, NY, USA), and 1% (*v*/*v*) penicillin/streptomycin (New Cell & Molecular Biotech, Suzhou, China). They were cultured in a humidified incubator at 37 °C with 5% CO_2_, and all cells were free of mycoplasma infection (Beyotime, Shanghai, China). MG132 (Selleck, Shanghai, China), bafilomycin A1 (CST, Boston, MA, USA), 3-methyladenine (Meilunbio, Suzhou, China), and U0126-EtOH (MCE, Shanghai, China) were all dissolved in DMSO (Aladdin, Shanghai, China) and chloroquine (CQ) (MCE, Shanghai, China) was dissolved in distilled water. In protein degradation assay, cells at a confluency of 70–90% were treated with DMSO, 10 μM MG132, 100 nM BafA, 20 μM CQ, and 5 mM 3-MA for 4–6 h. For inhibition assay, cells were treated with 1 μM U0126-EtOH for 24 h. Plasmids (Sangon Biotech, Shanghai, China) and siRNAs (GenePharma, Shanghai, China) were transfected through jetPrime transfection reagents (Polyplus Transfection, Strausberg, France) following the instructions from the manufacturer’s protocols.

### 2.2. Human Tissue Samples

30 paired CRC tissue samples, 20 paired gastric cancer tissue samples, 20 paired lung cancer tissue samples, 16 kidney cancer tissue samples, and 16 liver cancer tissue samples (Table S5) were all collected immediately after surgical resection. All were with the patients’ written informed consent and approval from the Institutional Review Board of Hangzhou Cancer Hospital (Permit Number: HZCH-2017-09).

### 2.3. Plasmid Construction

To construct plasmid LINC01234ORF-FLAG-pcDNA3.1+ (pORF-FLAG), the sequence of LINC01234ORF linked with one FLAG tag was amplified and inserted into the plasmid pcDNA3.1+ (Sangon Biotech, Shanghai, China). To generate GFP-carrying plasmid LINC01234ORF-FLAG-pCDH-CMV-MCS-EF1-copGFP-T2A-Puro (ORF-GFP), the sequence of LINC01234ORF linked with one FLAG tag was amplified and inserted into the plasmid pCDH-CMV-MCS-EF1-copGFP-T2A-Puro (SBI, Tokyo, Japan). For mutated plasmids pORFmut-FLAG and ORFm-GFP, in which the start codon ATG was mutated to ATT, the sequences mentioned above went through point mutation before being inserted into the corresponding plasmids.

### 2.4. Antibody Generation

We performed amino acid sequencing for one pair of CRC samples with mass spectrometry LTQ Orbitrap Elite (Thermo Fisher, Waltham, MA, USA). Based on the analysis of prediction of epitopes and specificity of peptide sequences, the anti-MBOP polyclonal antibody which targeted the short peptide PSDHASVWGNEDQPR was raised against rabbits. The process of analysis and production was performed by Abclonal Technology (Wuhan, China).

### 2.5. Animal Assay

For in vivo tumor cell proliferation assay, some four-week-old female BALB/c nude mice (Charles River, Jiaxing, China) were subcutaneously injected with 8 × 10^6^ co-GFP, ORF-GFP, and ORFm-GFP HCT116 cells, separately. After three weeks, mice were euthanized and suitable tumors from each group were minced and injected into the right axilla of another group of four-week-old female BALB/c nude mice. The body weight and tumor volume of each mouse were measured and recorded every three days. Tumor volumes were calculated with the formula V = ½ × a × b^2^, where a and b represented the length and width of the tumor, respectively. Relative tumor volume and body weight were normalized to the statistics recorded on the first day of measurements, and this procedure continued before the tumor volume of any one of them reached 2000 mm^3^ (day 22). Then, all mice were euthanized, and the tumors were collected to perform further analysis. During the whole process, mice were maintained under specific pathogen-free conditions with free access to food and water, and under a constant suitable temperature, humidity, and light cycle (12 h/12 h). All mice experiments followed the guidelines of Animal Welfare and were approved by the Zhejiang University Animal Care and Use Committee (Ethics Code: ZJU20200086).

### 2.6. Western Blot Analysis

Cell samples and tissue samples were lysed on ice in RIPA lysis buffer (Beyotime, Shanghai, China) supplemented with a protease inhibitor cocktail (Beyotime, Shanghai, China), and were quantified by a BCA kit (Beyotime, Shanghai, China). After being added with 5× loading buffer (Sangon Biotech, Shanghai, China) and boiled in boiling water for sample denaturation, equal amounts of protein samples were added to the wells of 18% Tris-tricine gel or 10–15% Tris-glycine gel, and target proteins were separated by electrophoresis (70 V for 25 min followed by 120 V for 85 min) and were transferred to 0.2 μm (Beyotime, Shanghai, China) or 0.45 μm (Millipore, Boston, MA, USA) PVDF membranes (200 mA, 40 min for MBOP and H3; 200 mA, 1.5 h for other proteins), followed by a 2 h blocking procedure with 5% skimmed milk in TBST buffer at room temperature. Next, the PVDF membranes were incubated with corresponding diluted primary antibodies (anti-MBOP (1:200, Abclonal), anti-GAPDH (1:10,000, Proteintech-60004-1-ig), anti-H3 (1:1000, Abcam-ab1791), anti-FLAG (1:1000, Multi Sciences-70-ab002-040), anti-MEK1/2 (1:1000, Cell Signaling Technology-9122S), anti-phospho-MEK1/2 (1:1000, Cell Signaling Technology-9154T), anti-ERK1/2 (1:1000, Cell Signaling Technology-4695T), anti-Phospho-ERK1/2 (1:1000, Cell Signaling Technology-4370), anti-MMP2 (1:1000, Abclonal-A19080), anti-MMP9 (1:1000, Abclonal-A0289), anti-RMND5A (1:1000, Proteintech-17559-1-AP), and anti-MAEA (1:1000, Proteintech-28363-1-AP)) at 4 °C overnight. After replacing the diluted primary antibodies with corresponding secondary antibodies (Goat anti-Mouse IgG (H+L) HRP (1:5000, Multi Sciences-70-GAM0072), Goat anti-Rabbit IgG (H+L) (1:5000, Multi Sciences-70-GAR0072), HRP-conjugated IgG fraction monoclonal mouse anti-rabbit IgG, and light-chain specific (1:5000, Proteintech-SA00001-7L)), the PVDF membranes were incubated at room temperature for 2 h. Finally, the secondary antibodies were washed off with TBST buffer, and the target protein bands were exhibited with the help of ultra-sensitive ECL chemiluminescence substrate (4A Biotech-4AW011-500, Beijing, China) in a ChemiDoc Touch Imaging System (Bio-Rad, Hercules, CA, USA).

### 2.7. Real-Time Quantitative PCR

RNA samples from cells and tissues were extracted by RNA extraction kit (Axygen, San Francisco, CA, USA), followed by concentration detection by Nanodrop 2000 (Thermo, Waltham, MA, USA). Then the RNA samples (1 μg) were reverse-transcribed (TAKARA, Osaka, Japan) to cDNA. The whole reaction system contained nuclease-free H_2_O, template cDNA, paired primers, and SYBR Premix Ex Taq (TAKARA, Osaka, Japan), and was implemented by StepOnePlus Real-Time PCR System (Applied Biosystems, Waltham, MA, USA). Relative gene expressions were presented after being normalized to that of GAPDH mRNA. Primer information is listed in Table S6.

### 2.8. Cytoplasmic and Nucleic RNA/Protein Extraction

PARIS™ Kit (Invitrogen-AM1921, Carlsbad, CA, USA) was used to separate cytoplasmic and nucleic parts from the whole-cell samples to exhibit the subcellular localization of MBOP and *LINC01234ORF*.

### 2.9. Cell Migration Assay with Transwell Chambers

Cell migration assay was performed with transwell chamber apparatus with 8.0 μm pore membrane (Corning, New York, NY, USA). First, 2 × 10^4^ treated cells suspended in 200 μL serum-free culture medium were loaded onto the upper transwell chamber apparatus, and 600 μL culture medium with 15% (*v*/*v*) FBS was loaded onto the lower compartment as chemoattractant. After 48 h of incubation, the transwell chambers were immersed in 4% paraformaldehyde (Servicebio, Wuhan, China) for 20 min as fixation, followed by incubation with crystal violet buffer for another 20 min. Cells on the upper surface of the transwell chambers were wiped off with cotton swabs. Finally, the migrated cells on the lower surface of transwell chambers were recorded with Nikon eclipse Ti-S (Nikon, Tokyo, Japan).

### 2.10. Wound-Healing Assay

Cells transfected with pcDNA3.1+, pORF-FLAG, and pORFmut-FLAG were plated to the 6-well plates. When the confluency reached 95%, a 200 μL pipette tip was used to scratch a straight line perpendicular to the cell surface, then PBS buffer was used to wash off the non-adherent cells, and PBS buffer was then replaced by fresh medium with 1% FBS. After doing this procedure for all the three groups of cells, the plates were recorded immediately through Nikon eclipse Ti-S (Nikon, Tokyo, Japan). After 24 h or 48 h, second or third images were recorded to compare the migration abilities of treated cells.

### 2.11. Immunofluorescence Staining

Treated cells were plated on cell slides (WHB Scientific, Shanghai, China) in advance, then fixed by 4% paraformaldehyde (Servicebio, Wuhan, China) at room temperature, followed by cell permeabilization with 0.5% Triton X-100 (Sangon Biotech, Shanghai, China) and cell blockage with 10% goat serum (Servicebio, Wuhan, China). Then, the cell slides were immersed in primary antibody diluted buffer (MBOP, 1:200) in the dark at 4 °C overnight. On the second day, the diluted buffer was washed off, followed by incubation with secondary antibody Alexa Fluor 555-labeled Donkey anti-Rabbit IgG (H+L) (1:1000, Multi-Sciences-A0453) diluted buffer in the dark at room temperature for 1.5 h. Nuclei were counterstained by Hochest 33342 (Beyotime, 1:1000). Immunofluorescence images were captured through OLYMPUS IX83-FV3000 (Olympus, Tokyo, Japan).

### 2.12. Immunoprecipitation and Mass Spectrometry Assay

Immunoprecipitation (IP) and mass spectrometry assay were conducted to investigate the interacting proteins of peptide MBOP. Three 100 mm dishes of cells were transfected with plasmids pcDNA3.1+, pORF-FLAG, and pORFmut-FLAG at a confluency of 50–60%. After 48 h, cells were harvested with Pierce™ IP Lysis buffer (Thermo Fisher Scientific, Waltham, MA, USA) supplemented with Halt™ Protease Inhibitor Cocktail (Thermo Fisher Scientific, Waltham, MA, USA), and anti-FLAG affinity gel (Yeasen, Shanghai, China) was used to perform IP assay, then 12% SDS-PAGE was conducted to separate the interacting proteins. Next, protein bands with the most apparent discrepancies were cut to perform further mass spectrometry assay (Applied Protein Technology, Shanghai, China) and KEGG analysis.

### 2.13. Colony-Formation Assay

Lentiviral transfected cells co-GFP, ORF-GFP, and ORFm-GFP of HCT116 and HCT15 were planted onto the 6-well plates with a density of 1000 cells per well. All the cells were cultured in a complete culture medium with 20% (*v*/*v*) FBS. After 10–14 days, the culture medium was removed, and cells were immersed in 4% paraformaldehyde (Servicebio, Wuhan, China) for 20 min for fixation, followed by incubation with crystal violet buffer for 15 min. The images of formed clones were recorded with Nikon eclipse Ti-S (Nikon, Tokyo, Japan).

### 2.14. Statistical Analysis

All the data results were shown as mean ± SD through the software GraphPad Prism 6 (GraphPad Software, San Diego, CA, USA). Differences among groups were analyzed with two-way analysis of variance (ANOVA), while the differences among one group were analyzed with unpaired *t*-test or one-way ANOVA. * *p* < 0.05, ** *p* < 0.005, *** *p* < 0.0005 and **** *p* < 0.0001 were considered as significant.

## 3. Results

### 3.1. LINC01234 Has Ribosome Binding Sites across Several Perspectives

**Figure 1:**
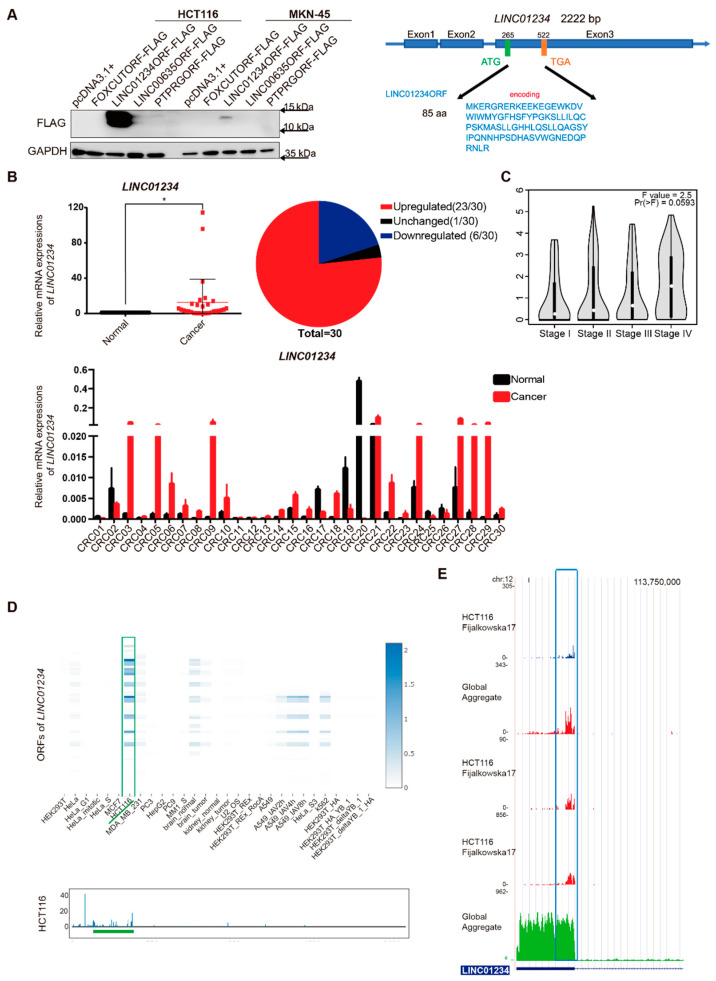
*LINC01234* has ribosome binding sites across several perspectives. (**A**) The translation abilities of some open reading frames (ORFs) encoded from long noncoding RNAs (lncRNAs) were tested in colorectal cancer (CRC) cell line HCT116 and gastric cancer cell line MKN-45, and LINC01234ORF showed positive signals (left panel). This ORF was contained in the exon 3 of *LINC01234* (right panel). (**B**) *LINC01234* was overexpressed in the human CRC tissues compared to the adjacent noncancerous tissues (*n* = 30), data are presented as mean ± SD, * *p* < 0.05. (**C**) *LINC01234* gradually increased in CRC from stage I to stage V. (**D**) Ribosome footprint profiling dataset POSTAR3 indicated that HCT116 owned the highest expression of ORFs originating from *LINC01234*, and it pointed out the same possible translation region as the right panel of (**A**). (**E**) GWIPS-viz browser showed the identical ribosome footprints’ peaks of *LINC01234* with (**A**,**D**) in HCT116.

An increasing number of naturally existing peptides encoded by long noncoding RNAs (lncRNAs) have been identified to play important roles in tumorigenesis and progression [6,7,8], which greatly inspired relevant research progress. Thus, we initially analyzed the coding potential of abundant lncRNAs using the above-mentioned databases RegRNA 2.0 [12], CPC [10], and CPAT [11], and identified 32 candidate lncRNAs (Table S1). Then, some of the open reading frames (ORFs) with FLAG-tags linked to their C termini were inserted into the plasmid pcDNA3.1+, and the FLAG signal was exhibited in colorectal cancer (CRC) cell line HCT116 and gastric cancer cell line MKN-45 through Western blot only by LINC01234ORF-FLAG. Moreover, the FLAG signal in HCT116 cells was more than tenfold stronger than that in MKN-45 cells (Figure 1A).

We further explored the rationale for this phenomenon. First, the mRNA expression of *LINC01234* in paired tissue samples from patients with CRC, lung cancer, kidney cancer, liver cancer, and gastric cancer was detected (Table S5). It was found that *LINC01234* was most significantly overexpressed in CRC (*n* = 30) (* *p* < 0.05) (Figure 1B and Figure S1A). Besides, the expression of *LINC01234* gradually increased in CRC from stage I to stage V (Figure 1C). In addition, the ribosome footprint profiling dataset POSTAR3 [14] showed that tens of ORFs of *LINC01234* were mostly expressed in HCT116 rather than in other cell lines. The longest ORF, with a 258 bp sequence, shared an identical genetic locus and translatome signal with the previously mentioned one in HCT116 (Figure 1D). Moreover, the GWIPS-viz browser [13] exhibited the same translation status and ORF loci as *LINC01234* in HCT116 (Figure 1E). Furthermore, GSE139407 showed hundreds of ribosome read counts of *LINC01234* in CRC cell lines SW620 and SW480 [15] (Figure S1B).

Based on all the above results ranging from bench to databases, the 85-amino-acid (aa) LINC01234ORF was chosen for further investigation in CRC, and it was probably an oncogene for its overexpression in CRC.

### 3.2. LINC01234 Encodes an Endogenous Peptide Highly Expressed in CRC

**Figure 2:**
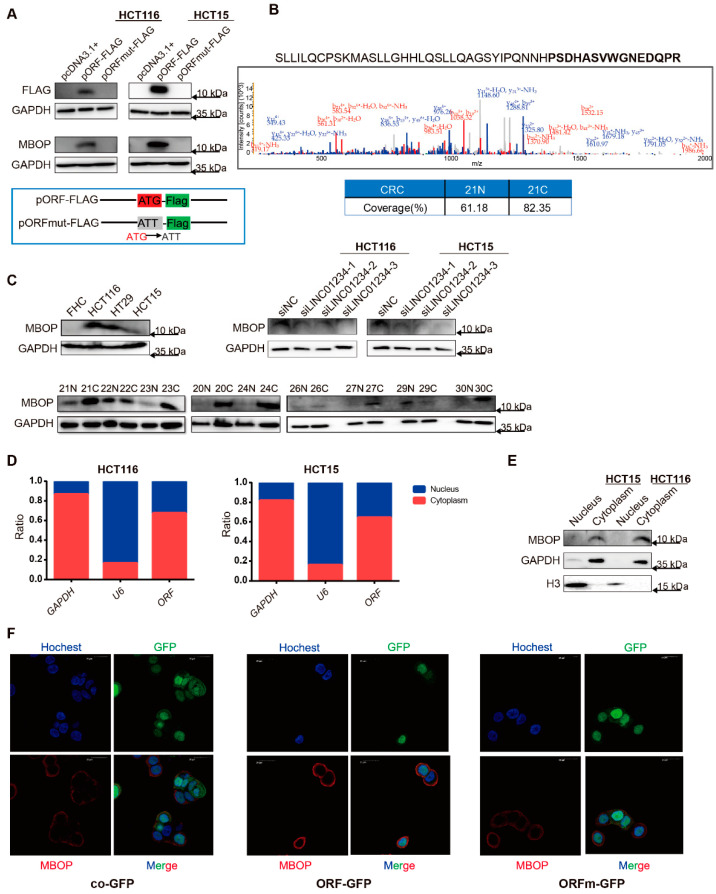
*LINC01234* encodes an endogenous peptide highly expressed in CRC. (**A**) A group of three plasmids, pcDNA3.1+, LINC01234ORF-FLAG (pORF-FLAG), and LINC01234ORFmut-FLAG (pORFmut-FLAG) (upper panel) was prepared to verify the coding potential of LINC01234ORF, and the corresponding antibody for MBOP was validated (lower panel). (**B**) Based on an amino acid mass spectrometry analysis, MBOP was a naturally existing peptide, and the part in bold was applied for the generation of an antibody of MBOP (upper panel) and a pair of CRC tissues exhibited higher sequence coverage in the cancerous one than in the noncancerous one. (**C**) Expression of MBOP in CRC cell lines and tissues, and siRNAs targeting *LINC01234* decreased the expression of MBOP. Note: The same siRNA-treated HCT116 samples from Figure 2C were used to detect the expression of MEK1 in Figure S4D. (**D**) The expression of *LINC01234ORF* and (**E**) MBOP were mostly localized in the cytoplasm of CRC cell lines HCT116 and HCT15. (**F**) Representative images of immunofluorescence of MBOP in lentiviral transfected cell lines co-GFP, ORF-GFP, and ORFm-GFP.

We revalidated the coding potential of LINC01234ORF by transfecting cell lines with plasmids expressing LINC01234ORF-FLAG (pORF-FLAG) and LINC01234ORFmut-FLAG (pORFmut-FLAG). When the start codon was mutated from ATG in pORF-FLAG to ATT in pORFmut-FLAG, only the former one showed a band (Figure 2A, upper panel), and the overexpressed peptide pORF-FLAG was in sequential consistency with the predicted ORF (Table S2). A pair of CRC tissues was used to investigate the sequential coverage of endogenously expressed peptide LINC01234ORF, if any. Mass spectrometry analysis showed that tissue sample 21C had 82.35% coverage, while 21N had 61.18% (Figure 2B, Table S3). Based on this result, a polyclonal rabbit antibody against the C terminus (68–82 aa) of LINC01234ORF was generated, and the existence of LINC01234ORF, which we termed MBOP, was validated in HCT116 cells (Figure 2A, lower panel), other cell lines, and tissue samples from patients with CRC (Figure 2C). CRC cell lines HCT116, HCT15, and HT29 expressed more MBOP than the normal colonic epithelial cell line FHC. Cancerous tissue samples from patients with CRC showed higher MBOP expression than those in paired adjacent noncancerous tissue samples. The knockdown of lncRNA *LINC01234* resulted in lower MBOP expression. In addition, the expression of MBOP was detected in several types of cancer cell lines, and we found relatively higher expression in CRC cell lines than in others (Figure S2A).

To gain insight into whether MBOP was a conserved peptide, the UCSC browser and UniProt browser were utilized to detect alignments of *LINC01234ORF* and MBOP with those of other species. It was found that *LINC01234ORF* shared a common sequence only with Rhesus monkeys and MBOP in parallel owned non-matched hit with many other species such as mice and dogs (Figure S2B). Western blot detecting the existence of MBOP in organs of a female BALB/c nude mouse, however, showed a signal in the colon around 10 kDa (Figure S2C). In addition, the above-mentioned organs from the same mouse also showed certain amounts of existence of *LINC01234* and *LINC01234ORF* (Figure S2D). Thus, it could be concluded that MBOP was not only conserved in primates.

Furthermore, the localization of MBOP in cancer cells was studied. Using subcellular fractionation followed by real-time quantitative polymerase chain reaction (RT-qPCR) of CRC cell lines HCT116 and HCT15, we found that the RNA sequence *LINC01234ORF* (simplified as *ORF*) mainly localized to the cytoplasm (Figure 2D), and Western blot analysis showed the corresponding localization situation of MBOP (Figure 2E). We then conducted an immunofluorescence assay with lentiviral transfected cell lines named as pCDH-CMV-MCS-EF1-copGFP-T2A-Puro (simplified as co-GFP), LINC01234ORF-GFP (ORF-GFP), and LINC01234ORFmut-GFP (ORFm-GFP) (Figure S2E), and the overexpressed MBOP situated more likely in the cytoplasm rather than in the nucleus (Figure 2F).

We also attempted to predict whether MBOP contained any transmembrane regions. The transmembrane hidden Markov model (TMHMM) server 2.0 [16] was used to predict the structure of MBOP, which turned out to be a naught transmembrane domain (Figure S2F). SignalP-5.0 server [17] and ProtScale [18] exhibited similar outputs to those of the TMHMM server (Figure S2G,H).

Therefore, we concluded that this endogenously expressed peptide, MBOP, was mainly localized in the cytoplasm, and was sequence-conserved across primates and mice.

### 3.3. MBOP Promotes CRC Progression through Cell Migration and Proliferation

**Figure 3:**
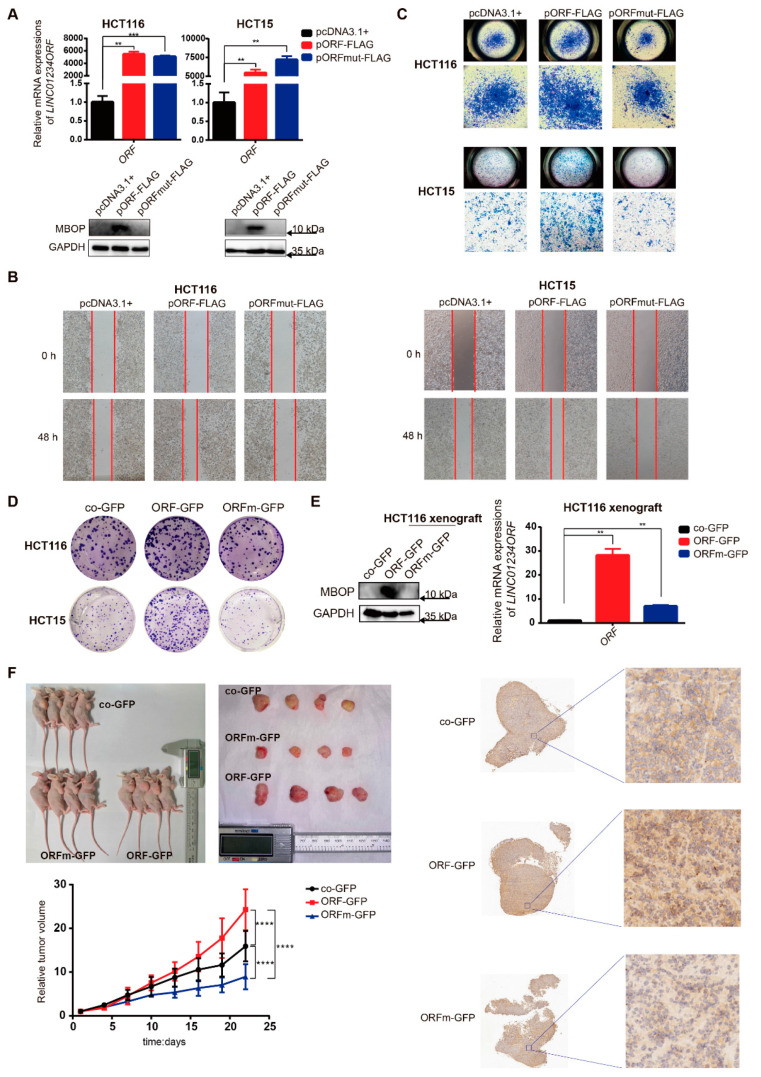
MBOP promotes CRC progression through cell migration and proliferation. (**A**) Representative images of expressions of *LINC01234ORF* and MBOP in HCT116 and HCT15 after transfection of series plasmids pcDNA3.1+, pORF-FLAG, and pORFmut-FLAG. Data are presented as mean ± SD, ** *p* < 0.005, *** *p* < 0.0005. (**B**) Migration ability of cells transfected with series plasmids pcDNA3.1+, pORF-FLAG, and pORFmut-FLAG were detected with wound healing assay. (**C**) Migration ability of cells transfected with series plasmids pcDNA3.1+, pORF-FLAG, and pORFmut-FLAG were detected with transwell assay. (**D**) Colony formation assays of lentiviral transfected cell lines co-GFP, ORF-GFP, and ORFm-GFP. (**E**) The mRNA and protein expressions of MBOP from primary tumors resulted from in vivo plant of lentiviral transfected HCT116 cell lines co-GFP, ORF-GFP, and ORFm-GFP. Data are presented as mean ± SD, ** *p* < 0.005. (**F**) Mice bearing tumor fragments derived from primary tumors of (**E**), and the relative tumor volumes were recorded (*n* = 4). Data are presented as mean ± SD, **** *p* < 0.0001. The representative images of the immunohistochemistry assay are shown in the right panel.

To characterize the functional behaviors of MBOP in CRC cells, series modified plasmids based on pcDNA3.1+ and co-GFP were used to transfect CRC cell lines to investigate the differences in phenotypes between groups. Based on successful transfections with pcDNA3.1+, pORF-FLAG, and pORFmut-FLAG (Figure 3A), pORF-FLAG substantially accelerated the migration of HCT116 and HCT15 cells, whereas pORFmut-FLAG showed inhibitory effects through wound healing assays and transwell assays (Figure 3B,C). We also used lentiviral transfected cell lines co-GFP, ORF-GFP, and ORFm-GFP to perform colony formation assays (Figure S3A). After plating 1000 cells in 6-well plates for approximately 10 days, we observed that the ORF-GFP substantially enhanced the colony formation compared to co-GFP, and the mutant ORFm-GFP slightly decreased this phenotype (Figure 3D). These data provided strong and direct evidence that MBOP encoded by *LINC01234*, rather than the pure RNA sequence LINC01234ORFmut, promoted oncogenic phenotypes of CRC cell lines.

To further investigate the role of MBOP in CRC tumorigenesis in vivo, we subcutaneously injected co-GFP, ORF-GFP, and ORFm-GFP HCT116 cells into BALB/c nude mice. We harvested tumor tissues from each group (Figure 3E) and cut them into pieces. Another batch of nude mice was inoculated with the above-mentioned tumor pieces. Tumor size and mouse weight were measured every three days. We observed significant tumor volume differences between these three groups in three weeks (**** *p* < 0.0001), and the ORF-GFP group noticeably promoted the growth rates of tumors, whereas the ORFm-GFP inhibited the growth rates (*n* = 4) (Figure 3F). Mice weights tended to decrease in all the three groups, but no remarkable difference was observed (Figure S3B).

In summary, MBOP promoted CRC progression through migration and proliferation.

### 3.4. MBOP Interacts with MEK1 in CRC

**Figure 4:**
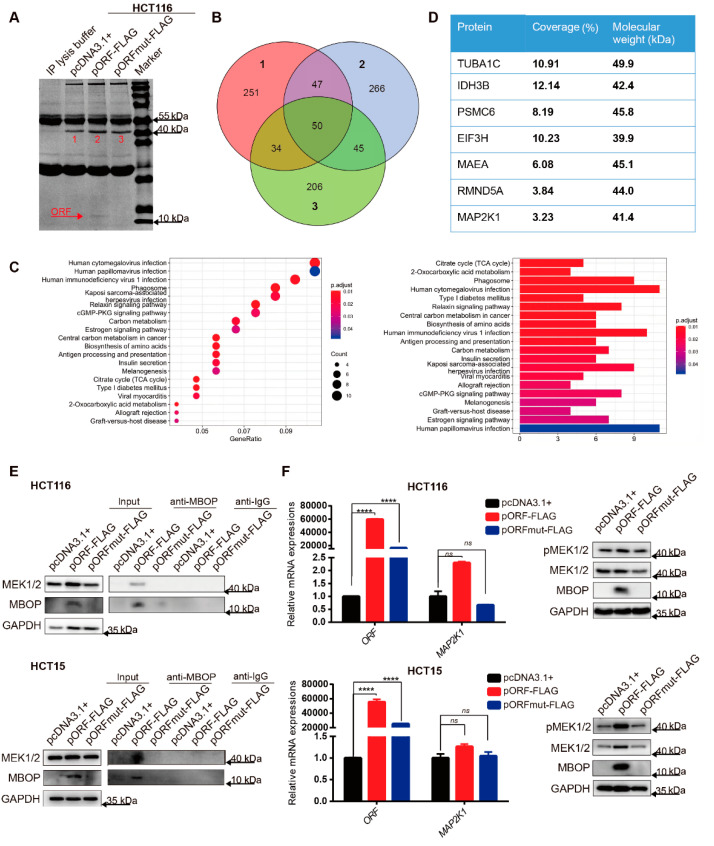
MBOP interacts with MEK1 in CRC. (**A**) Immunoprecipitation (IP) followed by mass spectrometry assay identified the interacting proteins of MBOP. (**B**) Venn diagram where 1 refers to pcDNA3.1+, 2 refers to pORF-FLAG, and 3 refers to pORFm-FLAG, delineated the range of potential interacting proteins—in total 266 candidate proteins. (**C**) KEGG analysis of the above-mentioned 266 proteins, and viral-infection relevant pathways ranked the top among all the pathways. (**D**) Sequential coverage and molecular weight of some candidate interacting proteins. (**E**) After overexpressing pcDNA3.1+, pORF-FLAG, and pORFmut-FLAG in HCT116 and HCT15, the direct interaction between MBOP and MEK1 was proved. (**F**) MBOP did not significantly alter the mRNA expression of *MAP2K1*, but substantially promoted the protein level of total MEK1 (pMEK1 included). Data are presented as mean ± SD, **** *p* < 0.0001. ns: not significant.

To further explore the oncogenic role of MBOP in CRC tumorigenesis, we performed immunoprecipitation (IP) assays with anti-FLAG antibody after transfection with series plasmids. As shown in Figure 4A, pORF-FLAG produced a band adjacent to 10 kDa, which proved the success of the whole procedure. In addition, we noticed one specific position across the three lanes; thus, these parts were cut to perform further mass spectrometry analysis.

Relationships between the detected proteins are illustrated using a Venn diagram (Figure 4B), and the 266 proteins exclusively in group 2 (pORF-FLAG) were the target of our research (Table S4). Through Kyoto Encyclopedia of Genes and Genomes (KEGG) pathway analysis, we found that the top-ranked pathways were mostly in the range of viral infections, which were highly related to the MAPK signaling pathways [19,20,21,22] (Figure 4C). The MAPK signaling pathway is ubiquitously hyperactivated in CRC [23]. As one of the most important members of the MAPK signaling pathway, MEK1 plays a key role in phosphorylating ERK1/2 [24]. Interestingly, we found that gene *MAP2K1* (or protein MEK1) was in the interacting protein list, with correlative oncogenic function roles and matched molecular weight, which led us to further analyze this protein (Figure 4D).

Upon overexpression of pcDNA3.1+, pORF-FLAG, and pORFmut-FLAG in HCT116 and HCT15 cells, we verified the interaction between MBOP and MEK1 using IP assays (Figure 4E). Based on this result, we further investigated the expression of *MAP2K1* and protein MEK1, and we found that the *MAP2K1* did not show a significant difference, whereas the total MEK1 and the phosphorylated MEK1 (pMEK1) showed a similar substantial upregulation through overexpression of MBOP Therefore, the overexpression of MBOP elevated the total MEK1 rather than merely pMEK1 (Figure 4F). In conclusion, MBOP not only interacted with MEK1 but also promoted the expression of MEK1.

### 3.5. The MBOP/MEK1/pERK/MMP2/MMP9 Axis in CRC

**Figure 5:**
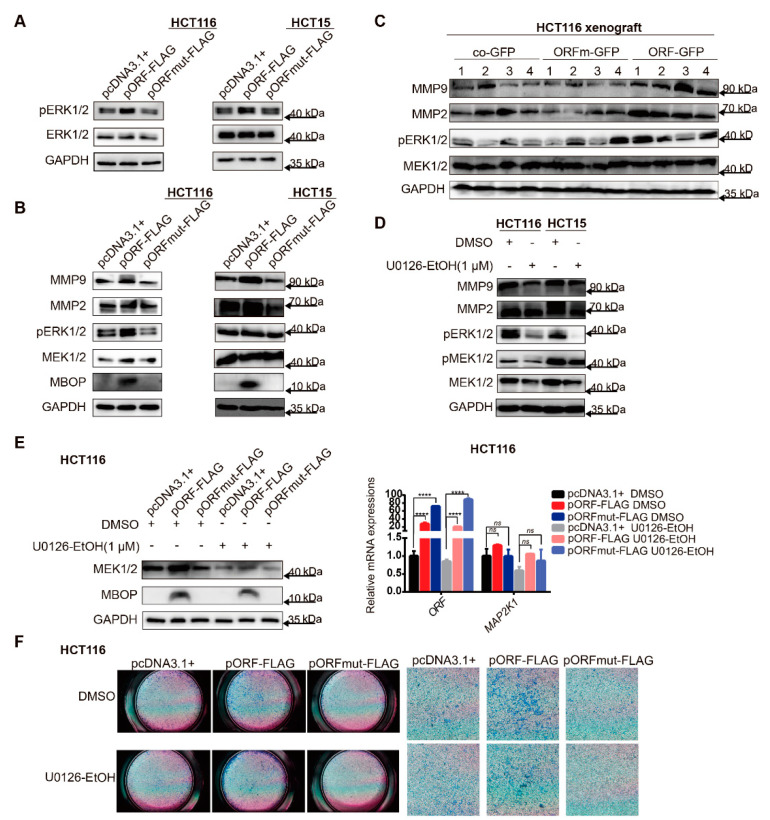
The MBOP/MEK1/pERK/MMP2/MMP9 axis in CRC. (**A**) MBOP substantially promoted the pERK1/2 in HCT116 and HCT15, but the total ERK expression did not show significant differences. (**B**) Overexpressing MBOP in HCT116 and HCT15 paved the axis of MBOP/MEK1/pERK/MMP2/MMP9. (**C**) The signaling pathway axis in animal assay met the expression tendency of in vitro assays (*n* = 4). (**D**) HCT116 and HCT15 were treated with the MEK1/2 inhibitor U0126-EtOH for 48 h, and the protein expression of MEK1/2, pERK1/2, and downstream MMP2 and MMP9 all decreased violently. (**E**) HCT116 cells were transfected with plasmids pcDNA3.1+, pORF-FLAG, and pORFmut-FLAG, followed by treatments of DMSO and U0126-EtOH. The overexpression of MEK1 and MBOP brought by transfecting cells with pORF-FLAG could be partially inhibited by U0126-EtOH, whereas the *MAP2K1* showed no significant alterations. Data are presented as mean ± SD, **** *p* < 0.0001. ns: not significant. (**F**) The pro-migration effect driven by the transfection of pORF-FLAG could be partially reversed by U0126-EtOH.

Having determined the oncogenic role and interacting partners of MBOP, we continued to investigate the underlying mechanisms. Based on previous research and experiments, the MAPK signaling pathway was probably activated by MBOP mainly by interacting with and promoting the expression of MEK1. Therefore, the expression of total ERK1/2 and phosphorylated (*p*-) state of ERK1/2, which represented the true activation status of the MAPK signaling pathway, were detected. We found that *p*-ERK1/2, rather than ERK1/2, showed substantial improvements in pORF-FLAG overexpressing cells (Figure 5A). We also detected the expression of matrix metalloproteinase 2 (MMP2) and 9 (MMP9), because they are two important metastasis-related proteins downstream of the MAPK signaling pathway [25,26]. Consistent changes were shown in Figure 5B, showing the upregulation of MMP2 and MMP9 in MBOP-overexpressing cells. In addition, the in vivo functional trial of MBOP showed that, although the interacting partner MEK1 was seldom upregulated because the overexpression of MBOP decreased violently during in vivo proliferation (Figure S4A), other related MAPK-related members, including p-ERK1/2, MMP2, and MMP9 were overexpressed in the MBOP-overexpressing group (n = 4) (Figure 5C), whereas the ORFm-GFP group showed the opposite results. We also investigated the necessity of binding with MEK1 for MBOP to promote the carcinogenesis of CRC. We used the MEK1 inhibitor U0126-EtOH [27], which was validated efficient in attenuating MEK1/2 and pMEK1/2 (Figure 5D). Then CRC cells were transfected with plasmids pcDNA3.1+*,* pORF-FLAG, and pORFmut-FLAG, followed by treatments of DMSO and U0126-EtOH (Figure 5E and Figure S4B). We found that the pro-migration effects caused by the enhancement of MMP2 and MMP9 driven by peptide MBOP could be partially reversed by the use of U0126-EtOH in HCT116 (Figure 5F) and HCT15 (Figure S4C). This outcome proved that MBOP needed to interact with MEK1 to play an oncogenic role in CRC.

We also investigated the MBOP/MEK1 axis. The overexpression of MEK1 brought by transfecting plasmid pORF-FLAG toward cells had been exhibited (Figure 4F). We found that the knockdown of MBOP suppressed MEK1 (Figure S4D). In contrast, overexpression of MEK1 decreased peptide MBOP, whereas knockdown of MEK1 increased MBOP (Figure S4E). Therefore, we speculated that the binding of MEK1 and MBOP was essential for both. MBOP was possibly an important co-factor of MEK1, and a feedback loop existed. When MEK1 was knocked down, MBOP would be upregulated to rescue the consequences brought by the decrease of MEK1, but the effects driven by the decreased expression of MEK1 weighed greater than the rescued impact brought by the overexpression of MBOP.

### 3.6. MBOP Is Degraded by the Ubiquitin–Proteasome System

MBOP was overexpressed in cancerous tissues and cell lines of CRC, however, the absolute content of MBOP was low. Therefore, we aimed to determine the reasons in this part.

**Figure 6:**
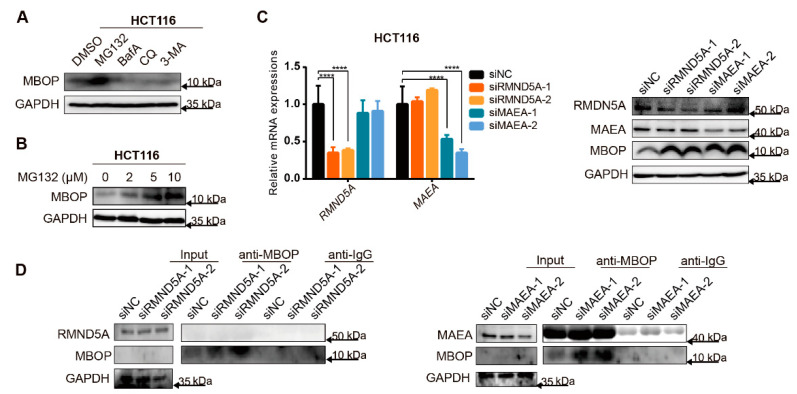
MBOP is degraded by the ubiquitin–proteasome system. (**A**) Cells treated with MG132 exhibited higher protein expressions of MBOP compared to other groups, indicating the possibility of involvement of the ubiquitin–protease system in the degradation process of MBOP. (**B**) The expressions of MBOP and the dose of MG132 showed a positive correlation. (**C**) Two E3-ligase enzymes RMND5A and MAEA were found in MBOP interacting candidate proteins in Table S4, and the siRNAs targeting RMND5A and MAEA were validated in HCT116. Knockdown of RMND5A and MAEA all decreased the degradation rate of MBOP. Data are presented as mean ± SD, **** *p* < 0.0001. (**D**) IP assay showed the direct interaction between MBOP and MAEA, rather than with RMND5A, which indicated that MAEA directly, while RMND5A indirectly, mediated the degradation of MBOP. The expressions of input MBOP in two IP assays were too low to be seen compared to those of the anti-MBOP groups.

We investigated the degradation situation of MBOP, through separately treating HCT116 with the proteasome inhibitor MG132, or the autophagy inhibitors bafilomycin A (BafA), chloroquine (CQ), and 3-methyladenine (3-MA) compared to DMSO for 5 h. We observed the upregulation of MBOP in the MG132-treated group (Figure 6A). In addition, different concentrations of MG132 were used to verify the previous results. We found a positive correlation between the dose of MG132 and the expression level of MBOP in lentiviral transfected cell lines HCT116 ORF-GFP (Figure 6B), which indicated that the degradation process of MBOP was probably conducted through the ubiquitin–proteasome system.

We further investigated the precise proteins that participated in this degradation process; therefore, we investigated the interacting proteins in Table S4. We found two relevant proteins: RMND5A and MAEA, which are members of the CTLH complex [28] that has E3-ligase enzymatic activity. We first verified the efficacy of siRNAs targeting RMND5A and MAEA (Figure 6C, left panel), and then treated the cells with MG132 for 5 h after the knockdown of RMND5A and MAEA. We found that both RMND5A and MAEA contributed to the degradation of MBOP (Figure 6C, right panel). IP assays confirmed that both RMND5A and MAEA participated in the ubiquitin–proteasome-system-relevant degradation of MBOP. However, MAEA seemed to have a more remarkable role in this process through direct binding with MBOP, whereas RMND5A only affected this degradation rather than being directly bound to MBOP (Figure 6D).

## 4. Discussion

We here characterized a peptide named MBOP, which originated from *LINC01234*. It had been clearly shown that MBOP was highly expressed in colon and CRC cell lines compared to other tissues and cell lines. Through in vivo and in vitro gain-of-function experiments, we proved it to be an oncopeptide. It also participated in the MBOP/MEK1/pERK/MMP2/MMP9 signaling pathway through interacting with MEK1. We also discovered that MAEA directly, and RMND5A indirectly, mediated the ubiquitin–protease-system-directed degradation of MBOP.

*LINC01234* is not a new topic in the tumor-related research field. Some researchers have reported the RNA sequence-related functions of *LINC01234* by interacting with miRNAs [29,30,31,32,33] and relevant proteins [34,35]. To the best of our knowledge, the MAPK signaling pathway is of high importance in the field of tumor-related therapy, especially in CRC [36,37,38], and the relationship between *LINC01234* and the MAPK signaling pathway has not been reported. Therefore, we are the first to present the interplay between MBOP encoded by *LINC01234* and the MAPK signaling pathway in CRC. This peptide-related functional investigation broadened our perspectives on the roles *LINC01234* plays in tumors. Based on this research, MBOP could be a candidate biomarker and therapeutic target for CRC. From this point of view, we were inspired to identify more hidden new faces in cancers.

However, our work also had some limitations, and raised questions that would be solved by further experiments. First, we performed an IP assay to identify the interacting partner of MBOP. Based on the KEGG analysis, MEK1, which had a relatively low peptide coverage, was chosen for further investigation. Nevertheless, it could not be concluded that the other protein candidates in Table S4 were not able to bind with MBOP. We not only hypothesized that MEK1 was a potential target based on its role in phosphorylating ERK1/2, but also we considered expanding our research pipelines to other candidates, for example, TUBA1C, PSMC6, and EIF3H.

Second, we determined the downstream destination of MBOP, the degradation directed by the ubiquitin–protease system and key players MAEA and RMND5A. However, we did not determine the upstream translation mechanism of MBOP. To the best of our knowledge, two main mechanisms drive the initiation of translation in eukaryotes: the canonical cap-dependent one and the novel cap-independent one. Circular RNAs possibly translate small peptides via a novel cap-independent mechanism that is mediated via the internal ribosome entry site (IRES) and N6-methyladenosine (m6A) [39]. However, the precise mechanisms steering translation of lncRNAs have not been clarified. Nevertheless, according to the results of the IP assay (Table S4), we found some potential members, such as EIF3H [40] and EEF1A1 [41], which were required for the enhanced translation of human tumors and tumorigenesis. Mining the specific proteins and mechanisms that drive the translation of MBOP will be part of our future studies.

## 5. Conclusions

We reported a naturally existing oncopeptide MBOP, encoded by *LINC01234*, which played an important role in the tumorigenesis of CRC by interacting with MEK1 and participating in the MAPK signaling pathway. Additionally, MBOP was degraded through the ubiquitin–protease system. We believe that MBOP would be a prognostic indicator of clinical examination, and more attention should be paid to noncoding RNA-derived peptides to explore potential targets for clinical therapy.

## Data Availability

The ribosome-bound RNA-seq data presented in Figure S1 is available in NCBI GEO datasets under accession number GSE139407.

## Supplementary Materials

The following supporting information can be downloaded at: https://www.mdpi.com/article/10.3390/cancers14092338/s1. Figure S1: *LINC01234* has ribosome binding sites across several perspectives; Figure S2: *LINC01234* encodes an endogenous peptide highly expressed in CRC; Figure S3: MBOP promotes CRC progression through cell migration and proliferation; Figure S4: The MBOP/MEK1/pERK/MMP2/MMP9 axis in CRC; Table S1: ORF prediction in lncRNAs; Table S2: Verification of LINC01234ORF; Table S3: Mass spectrometry of CRC tissue 21; Table S4: 266 candidate interacting proteins; Table S5: Paired cancer tissue samples from patients; Table S6: Primers and siRNA used in this research; File S1: Supplementary western blot results.
