# Supplementary material for: Oncopeptide MBOP Encoded by LINC01234 Promotes Colorectal Cancer through MAPK Signaling Pathway
Source: Cancers (Basel). 2022 May 9;14(9):2338. doi: 10.3390/cancers14092338 (PMC9100262; doi:10.3390/cancers14092338)

Oncopeptide MBOP encoded by *LINC01234* Promotes Colorectal Cancer through MAPK Signaling Pathway

Fig. 1A

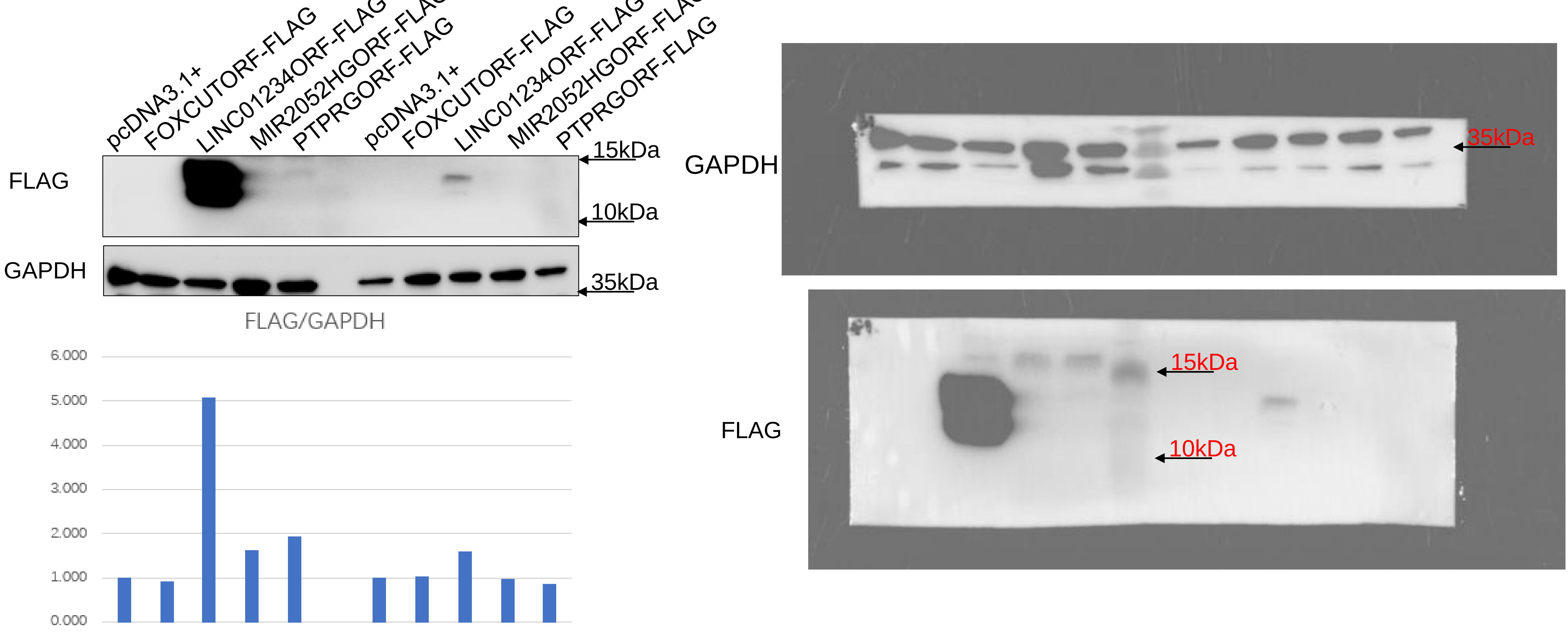

	pcDNA3.1+	FOXCUTORF-(	LINC01234O(	MIR2052H(	PTPRGORF-FLAG		pcDNA3.1-	FOXCUTOF	LINC01234	MIR2052H(	PTPRGORF-FLAG
FLAG	98856	96260	546581	175307	222393		96128	103346	165955	112638	101128
GAPDH	362742	383855	394200	396569	418736		428167	439842	459723	514436	519912
FLAG/GAPDH	0.27252427	0.250771776	1.38655758	0.442059	0.531106		0.224511	0.234962	0.360989	0.218954	0.19451
Ratio	1.000	0.920	5.088	1.622	1.949		1.000	1.047	1.608	0.975	0.866

Fig. 2A upper panel

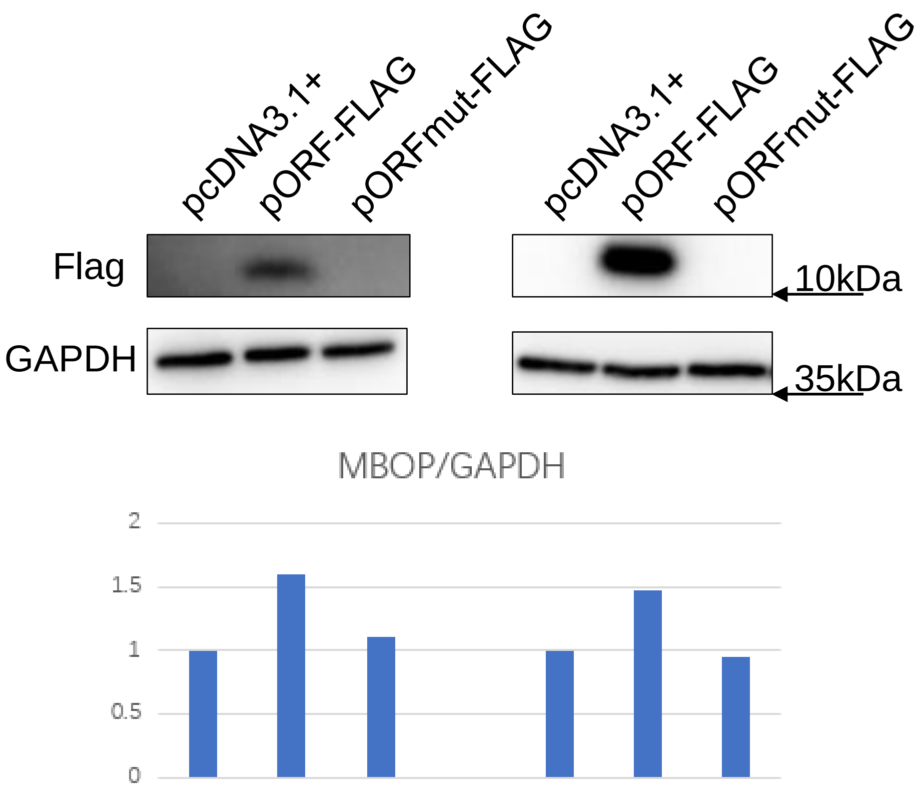

	pcDNA3.1	pORF-FLA	pORFmut-FLAG		pcDNA3.1	pORF-FLA	pORFmut-FLAG
FLAG	585223	842038	559618		985246	1493131	1013376
GAPDH	1716514	1545913	1487922		1086317	1122778	1177070
FLAG/GAPDH	0.340937	0.544687	0.376107		0.90696	1.329854	0.860931
ratio	1	1.597617	1.103157		1	1.466277	0.949249

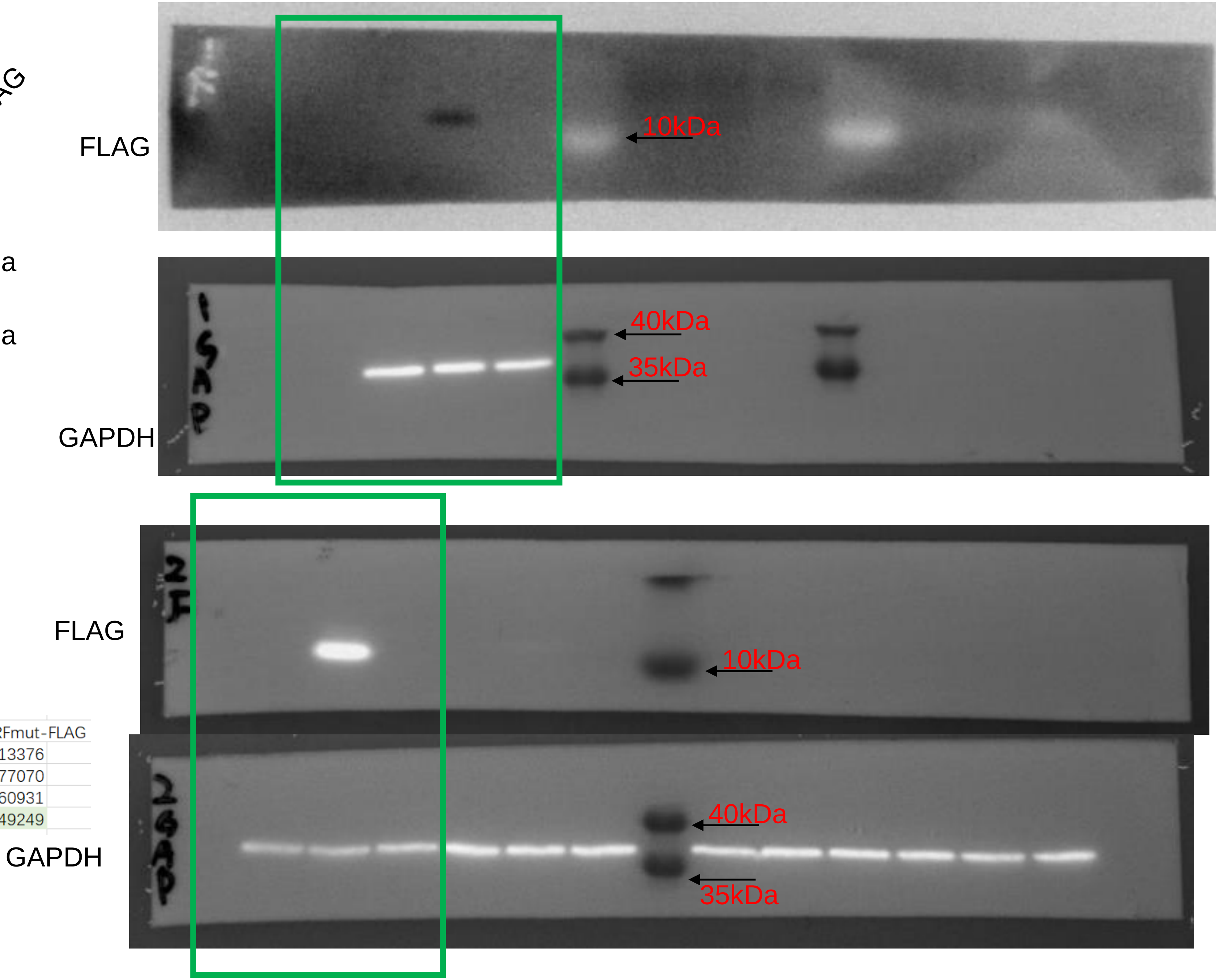

Fig. 2A lower panel

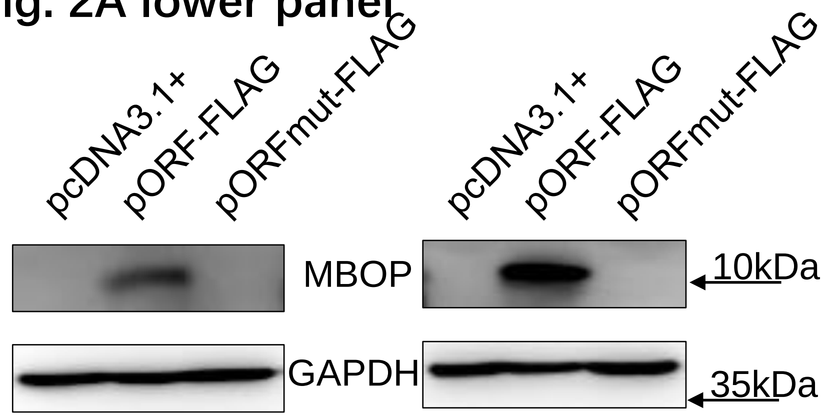

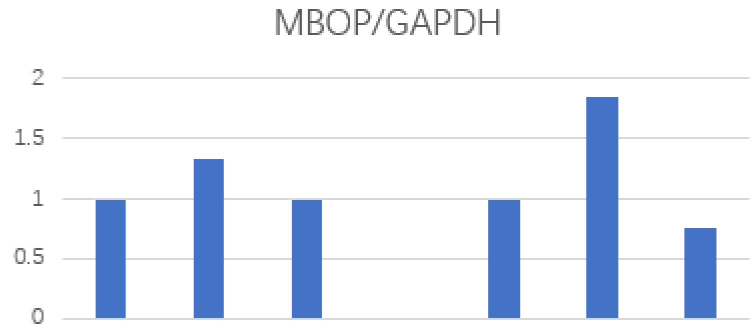

	pcDNA3.1	pORF-FLA	pORFmut-FLAG		pcDNA3.1	pORF-FLA	pORFmut-FLAG
MBOP	78134	102537	75134		95245	177011	70041
GAPDH	259671	256551	249463		264251	265873	258238
MBOP/GAPDH	0.300896	0.399675	0.301183		0.360434	0.665773	0.271227
ratio	1	1.328282	1.000953		1	1.847143	0.7525

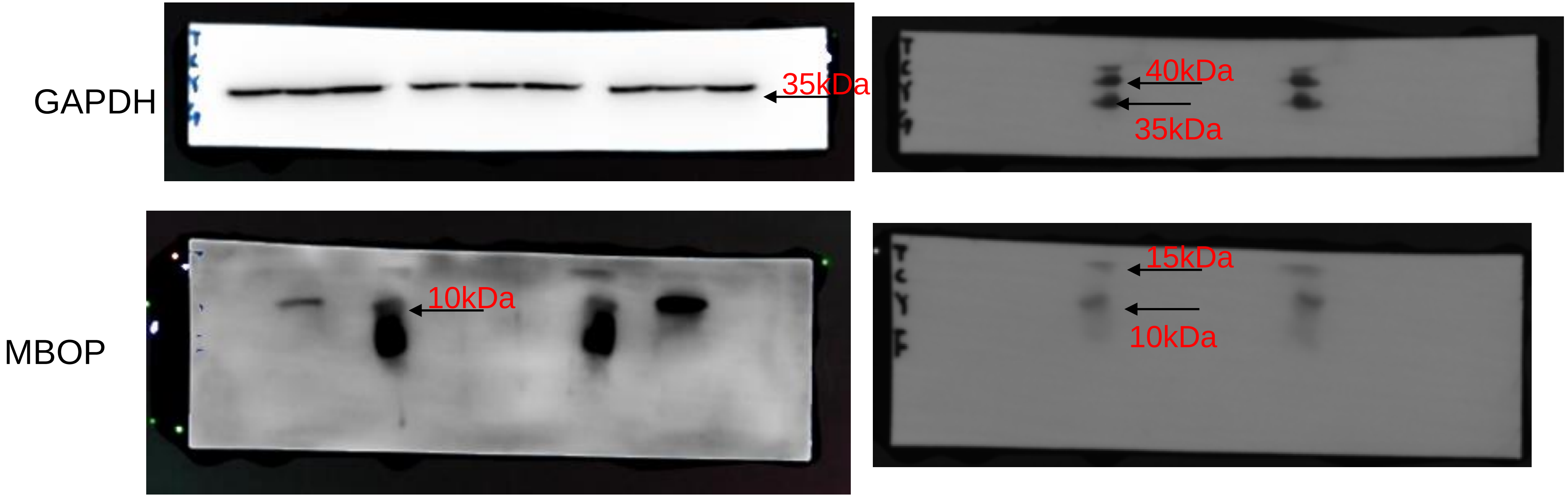

Fig. 2C left panel

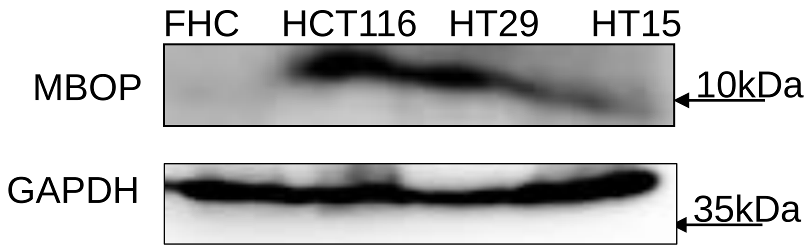

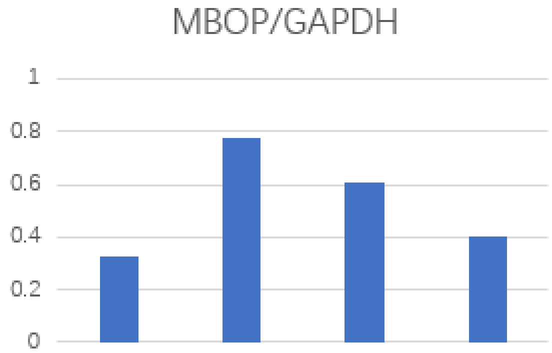

	FHC	HCT116	HCT15	HT29
MBOP	48884	93936	69580	58981
GAPDH	150049	121609	114339	146237
MBOP/GAPDH	0.325787	0.772443	0.608541	0.403325

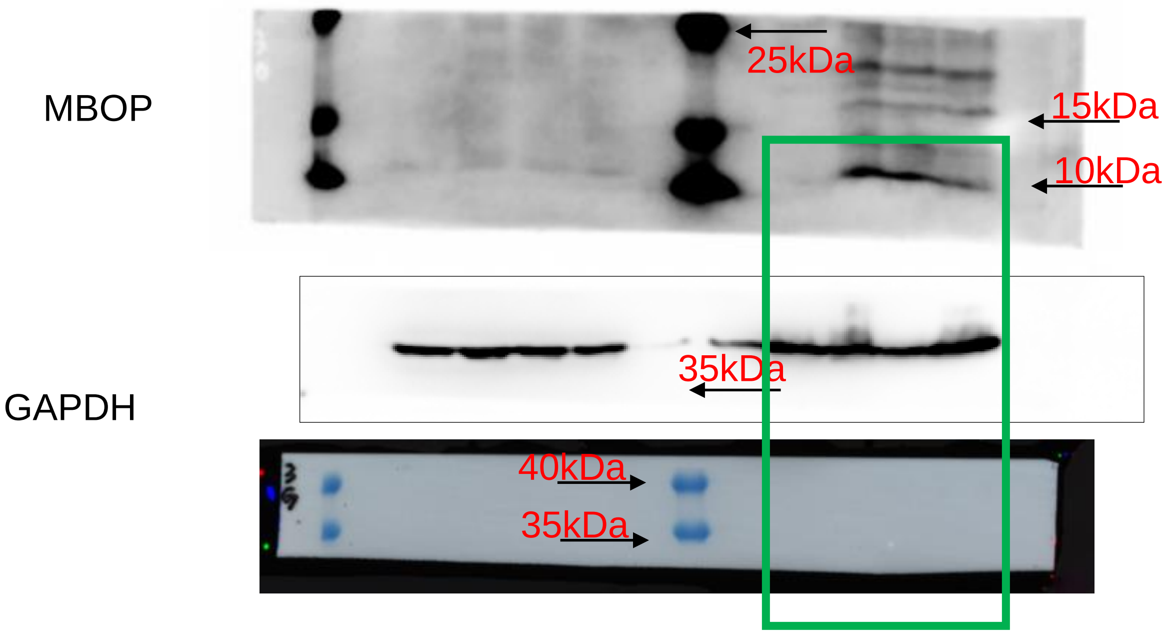

Fig. 2C upper and right panel

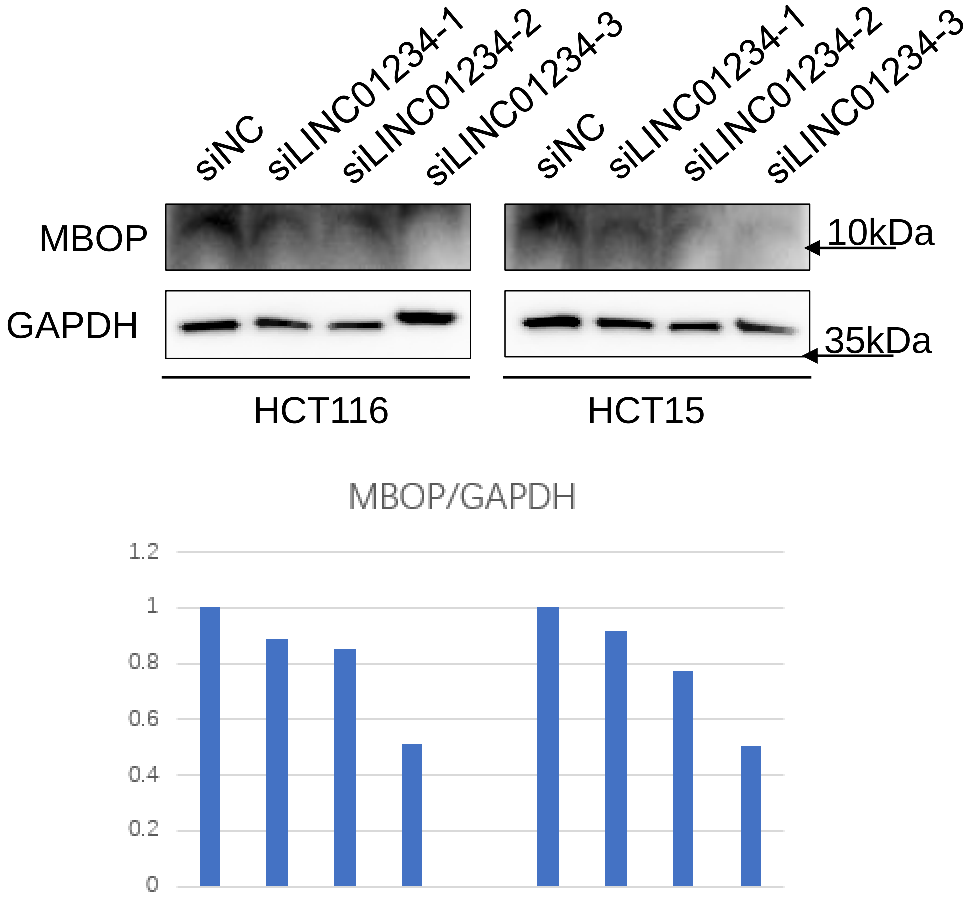

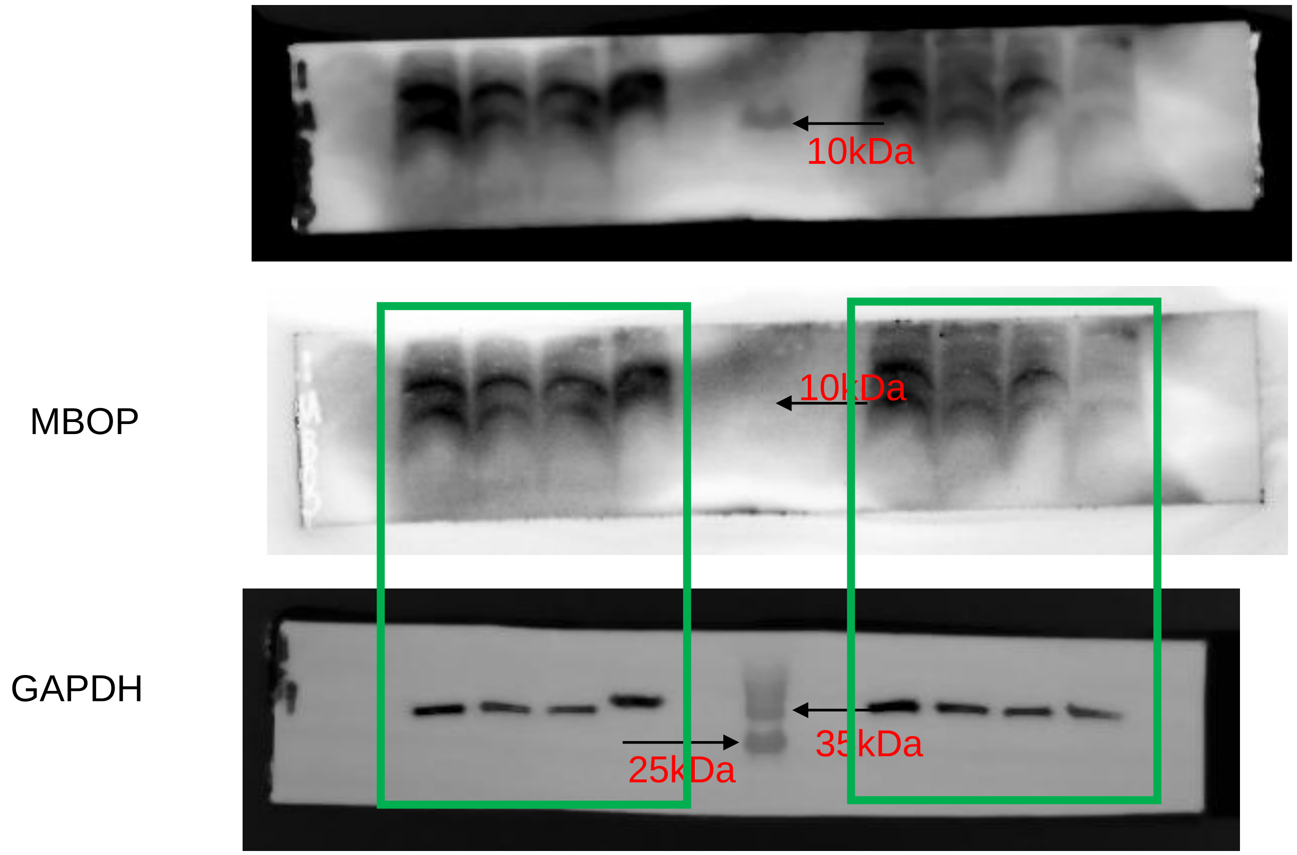

	siNC	siLINC0123	siLINC0123	siLINC01234-3	siNC	siLINC0123	siLINC0123	siLINC01234-3
MBOP	148609	113823	98009	86266	133730	106938	82750	57146
GAPDH	45307	39118	35208	51327	53753	47020	43222	45725
MBOP/GAPDH	3.280045	2.909735	2.783714	1.680714	2.487861	2.274309	1.914534	1.249776
ratio	1	0.887102	0.848682	0.512406	1	0.914162	0.76955	0.50235

Fig. 2C lower panel

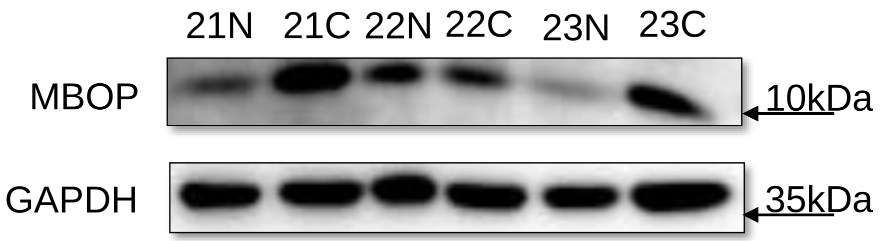

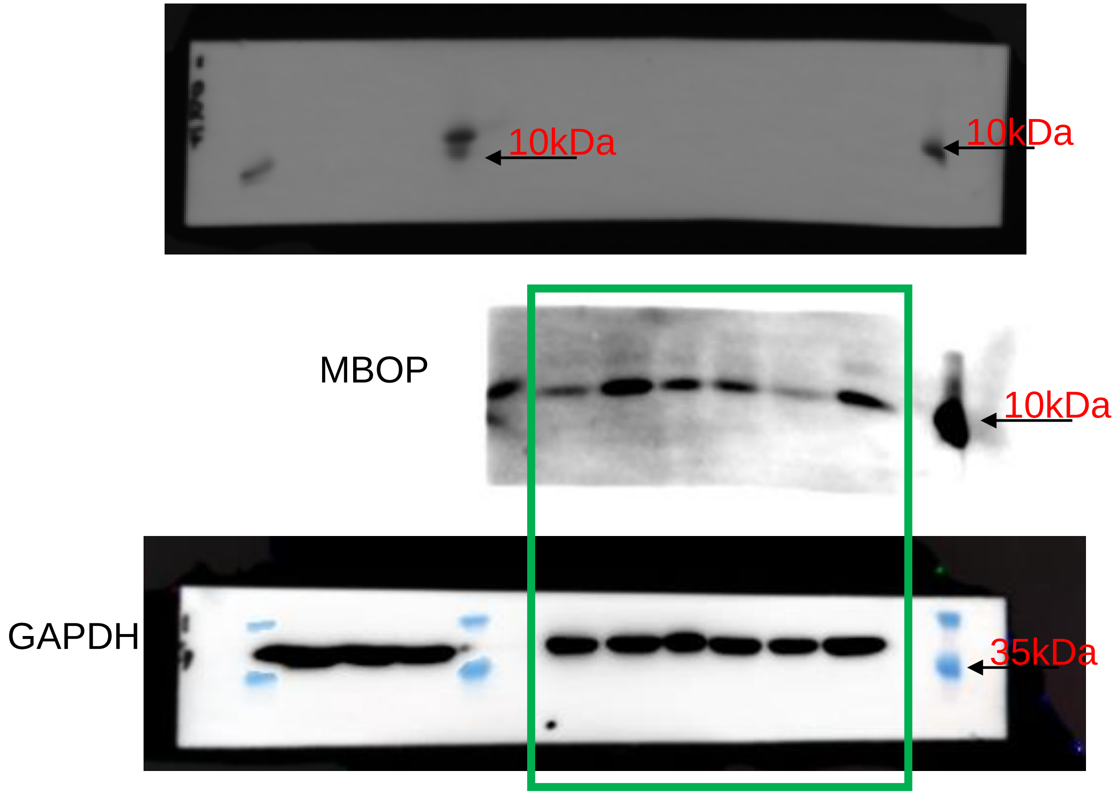

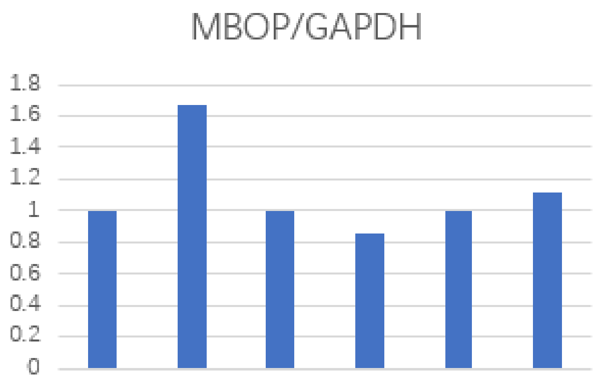

	21N	21C	22N	22C	23N	23C
MBOP	129285	222500	136363	132425	119320	156912
GAPDH	103575	106686	73807	83892	79823	93943
MBOP/GAPDH	1.248226	2.085559	1.847562	1.578518	1.494807	1.670289
ratio	1	1.670819	1	0.854379	1	1.117395

Fig. 2C lower panel

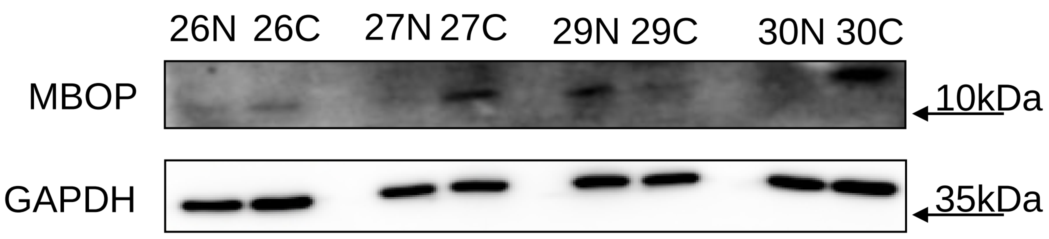

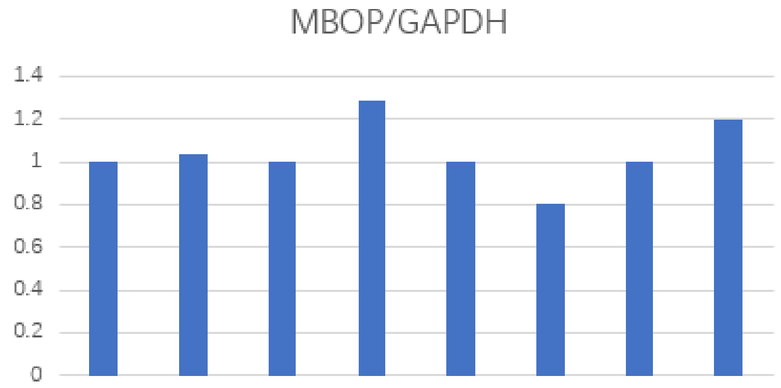

	26N	26C	27N	27C	29N	29C	30N	30C
MBOP	69012	85432	84964	129827	139820	101080	137254	190549
GAPDH	96671	115140	95454	113149	124458	111445	103041	119678
MBOP/GAPDH	0.713885	0.741984	0.890104	1.147399	1.123431	0.906994	1.332033	1.592181
ratio	1	1.03936	1	1.289061	1	0.807343	1	1.195301

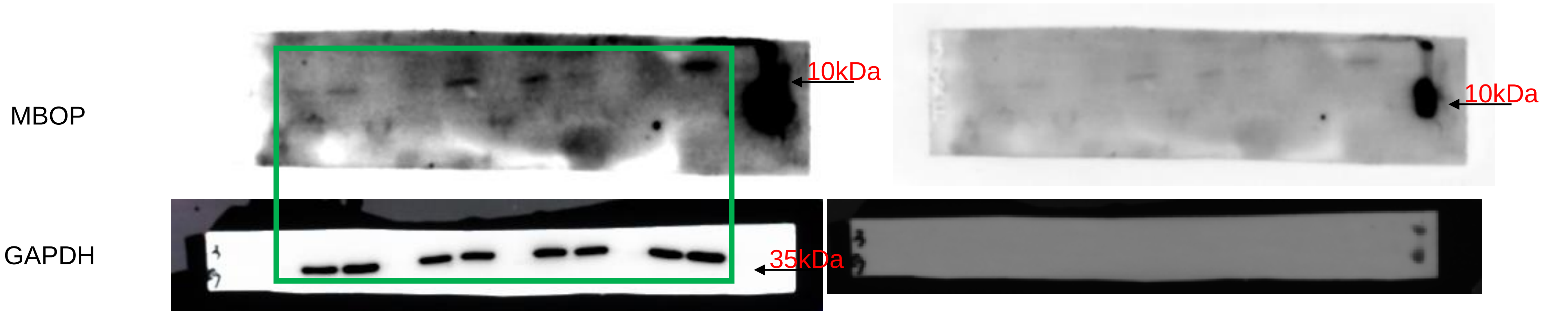

Fig. 2C lower panel

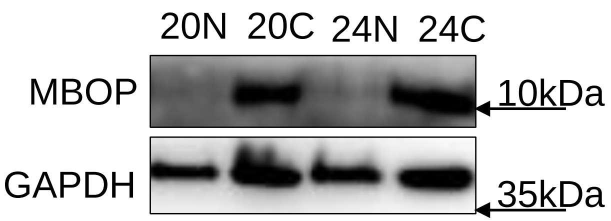

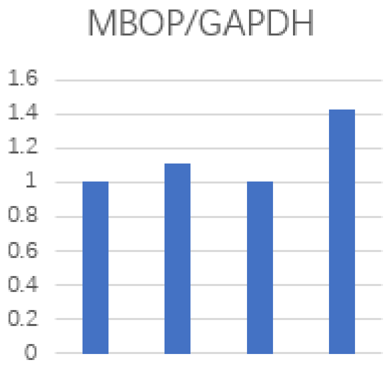

	20N	20C	24N	24C
MBOP	108790	147033	100445	152537
GAPDH	57975	70761	57301	61195
MBOP/GAPDH	1.876498	2.077882	1.752936	2.492638
ratio	1	1.107319	1	1.421979

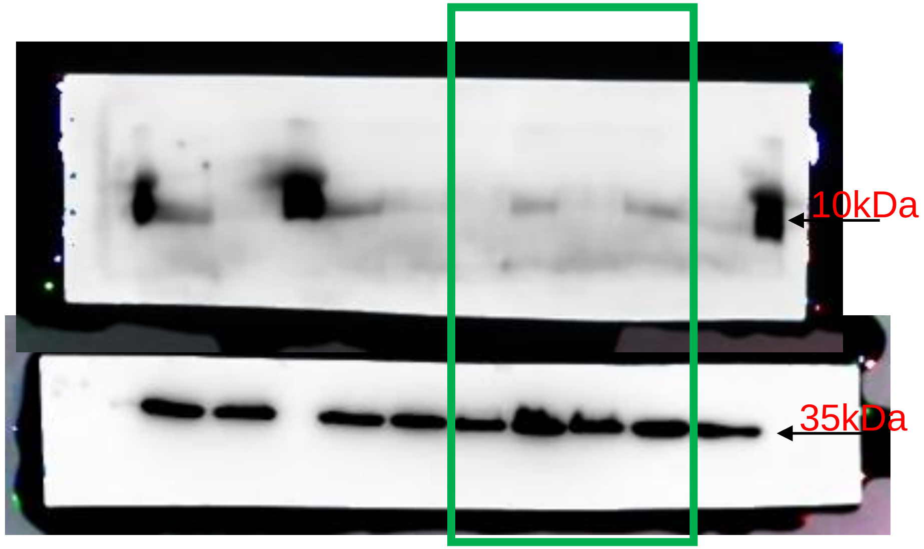

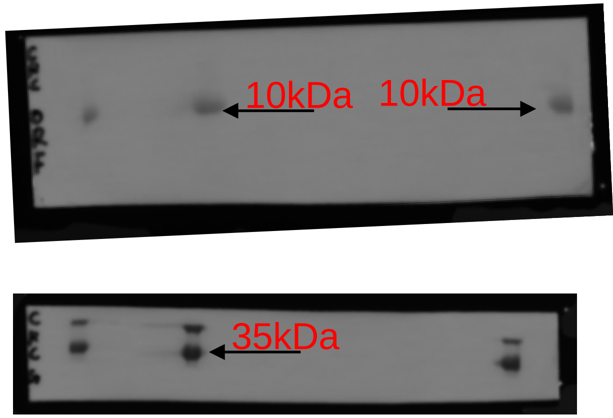

Fig. 2E

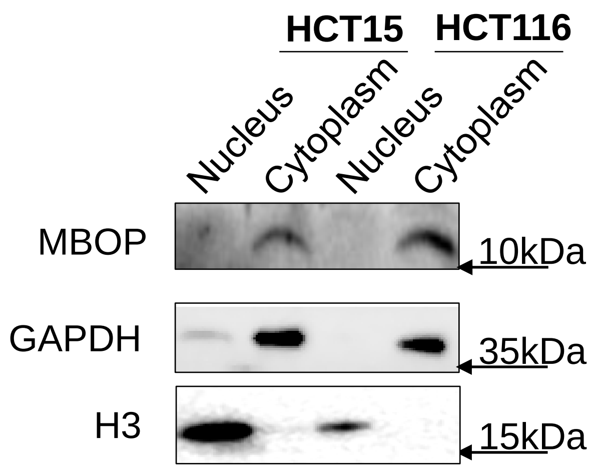

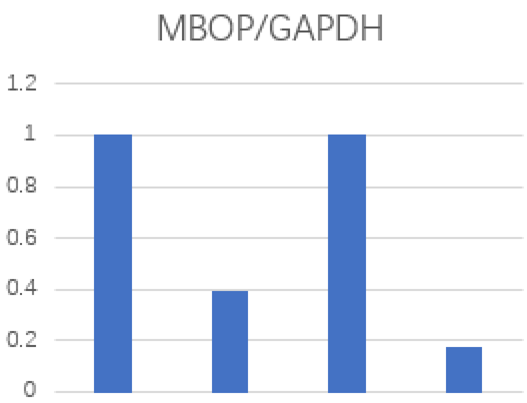

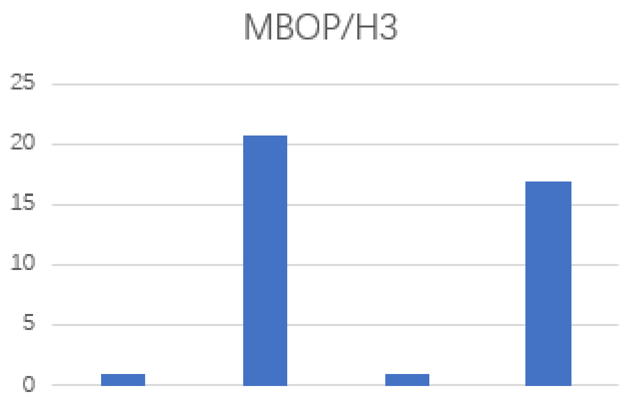

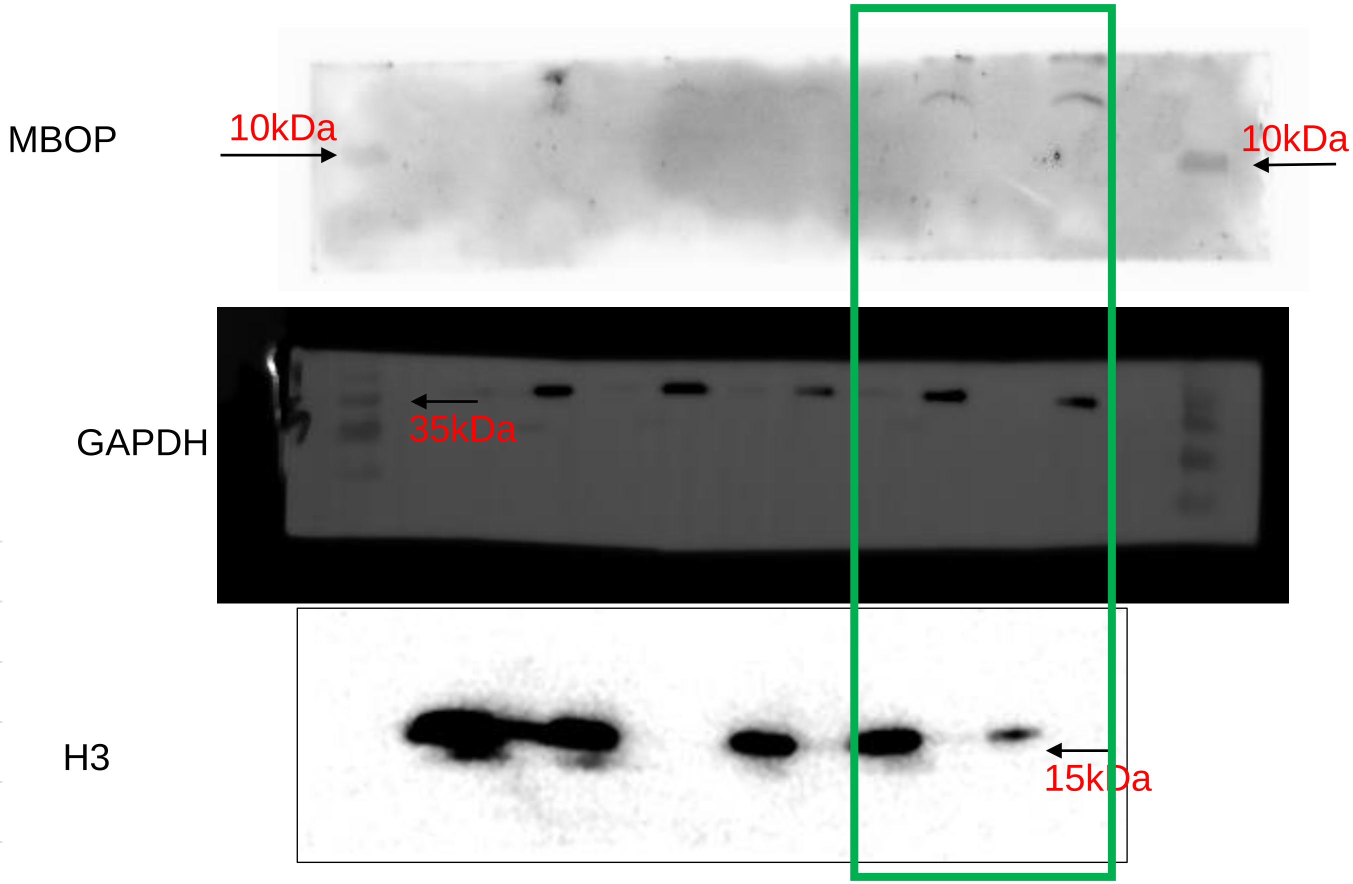

	Nucleus	Cytoplasm	Nucleus	Cytoplasm
MBOP	48032	74174	46029	65731
H3	136445	10201	36060	3038
GAPDH	17843	69381	5982	49490
MBOP/GAPDH	2.691924	1.06908231	7.6945838	1.328167
ratio	1	0.39714431	1	0.172611
MBOP/H3	0.3520246	7.27124792	1.2764559	21.63627
ratio2	1	20.6555093	1	16.95027

Fig. S2A

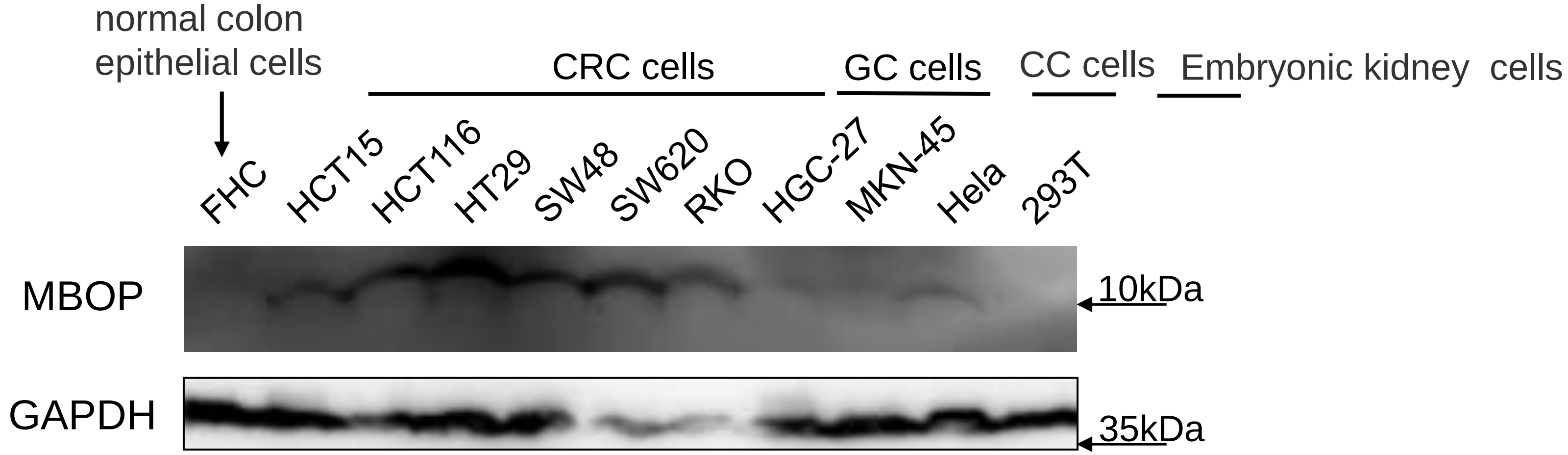

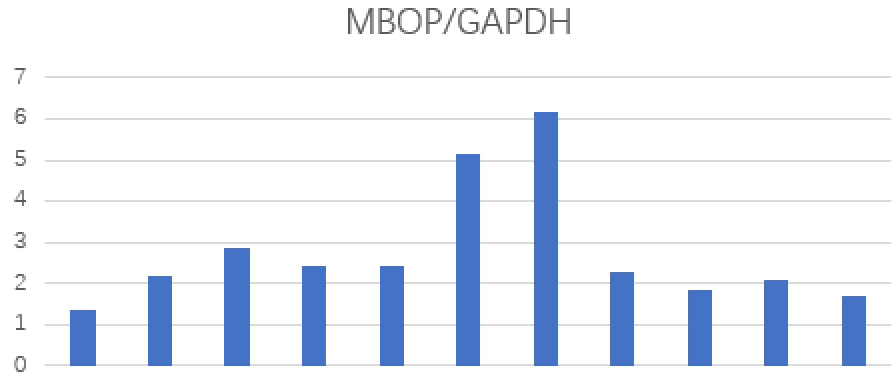

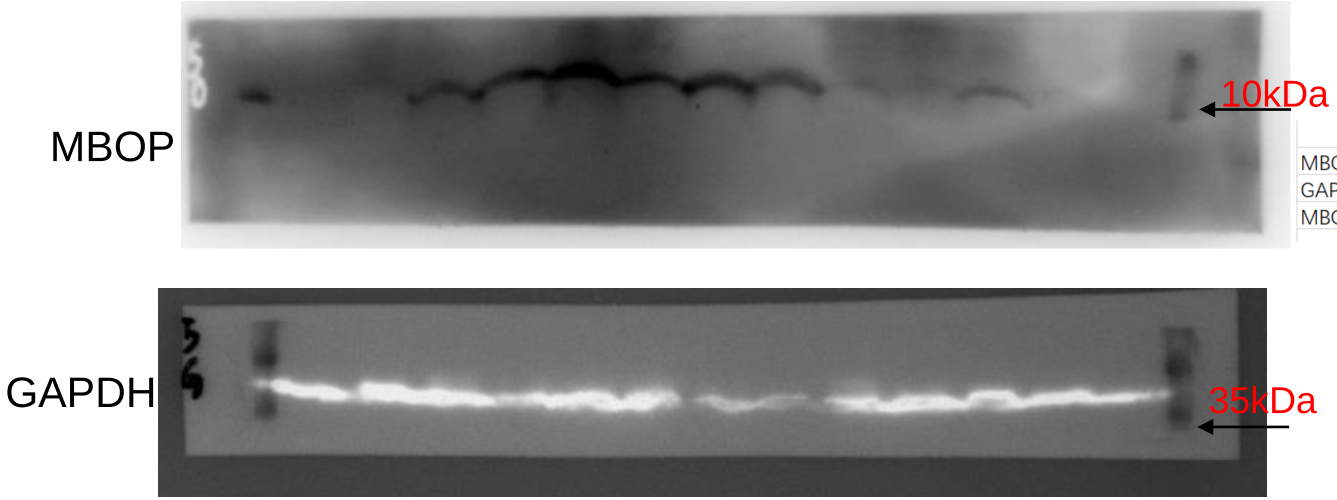

	FHC	HCT15	HCT116	HT29	SW48	SW620	RKO	HGC-27	MKN-45	Hela	293T
MBOP	1286339	2062766	2161783	2414520	2146712	2213243	1977691	1684676	1666453	1792625	1286339
GAPDH	939337	937202	757387	1001258	879142	429030	321753	735173	898572	855701	759519
MBOP/GAPDH	1.369412	2.200983	2.854265	2.411486	2.441826	5.158714	6.146612	2.291537	1.854557	2.09492	1.693623

Fig. S2C

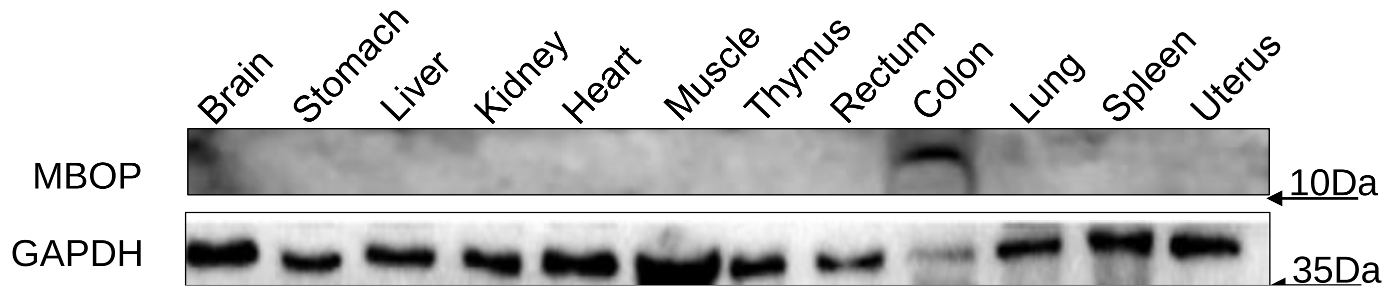

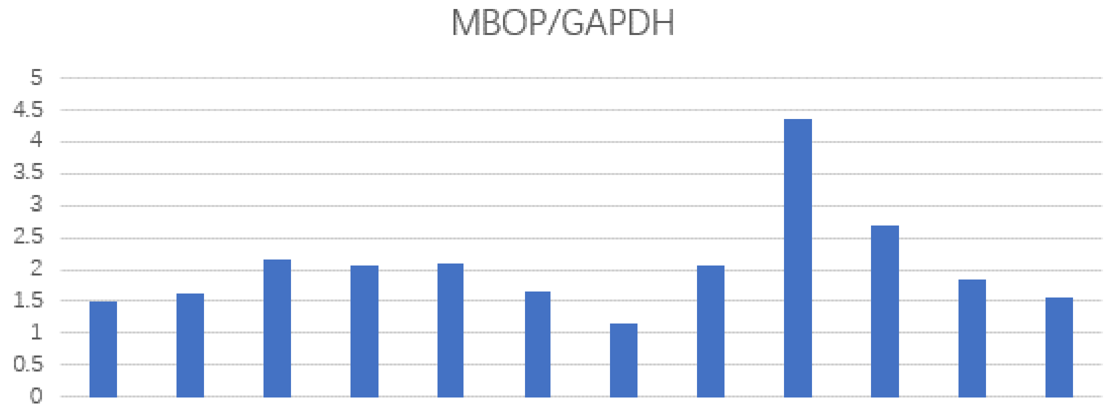

	Brain	Stomach	Liver	Kidney	Heart	Muscle	Thymus	Rectum	Colon	Lung	Spleen	Uterus
MBOP	81021	93829	96236	103366	104337	97536	96793	97248	176664	107344	103570	98961
GAPDH	54109	57182	44703	49694	49467	59156	83186	47085	40569	40044	55679	62848
MBOP/GAPDH	1.497366	1.640883	2.152786	2.08005	2.109224	1.648793	1.163573	2.065371	4.354655	2.680651	1.860127	1.574609

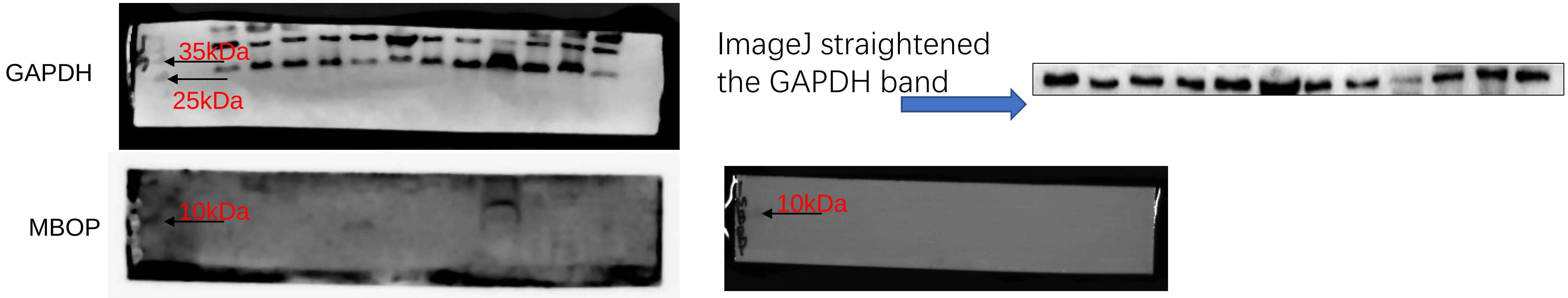

Fig. 3A

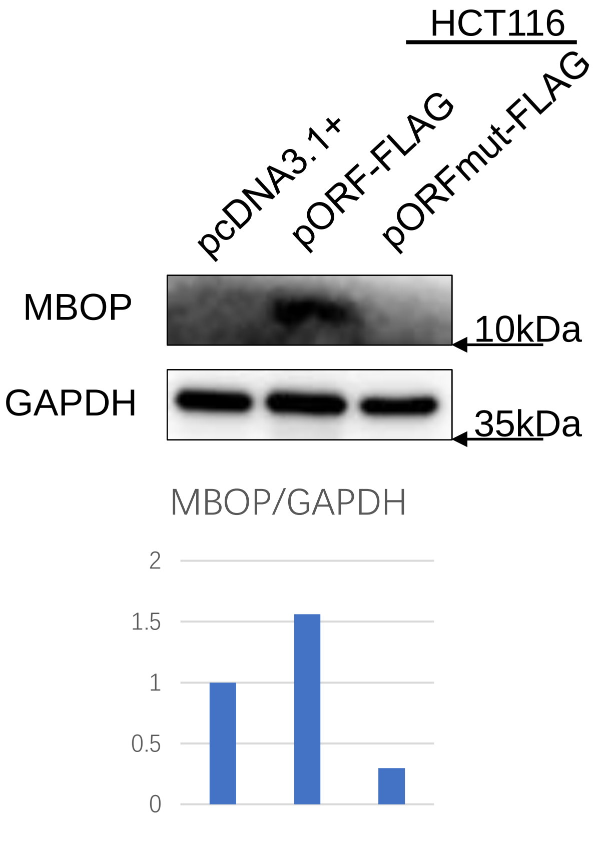

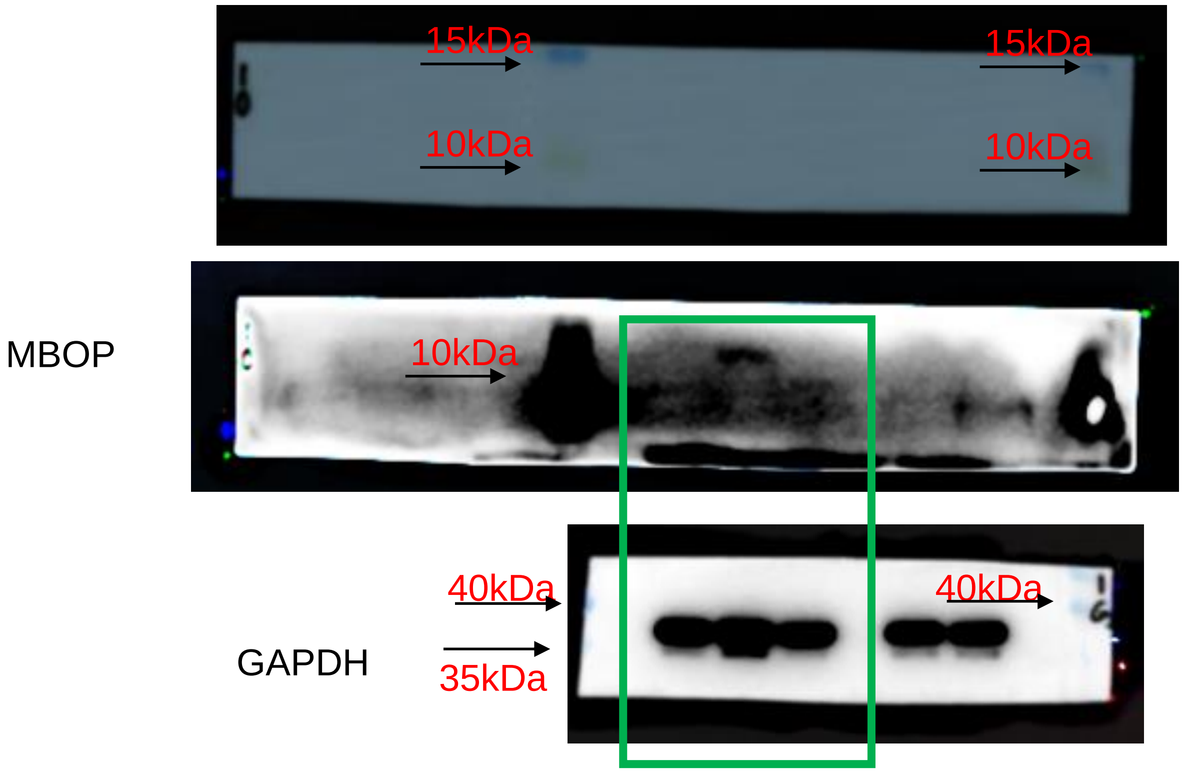

	pcDNA3.1	pORF-FLA	pORFmut-FLAG
MBOP	48086	86164	13039
GAPDH	63226	72513	58021
MBOP/GAPDH	0.760542	1.188256	0.224729
Ratio	1	1.562381	0.295485

Fig. 3A

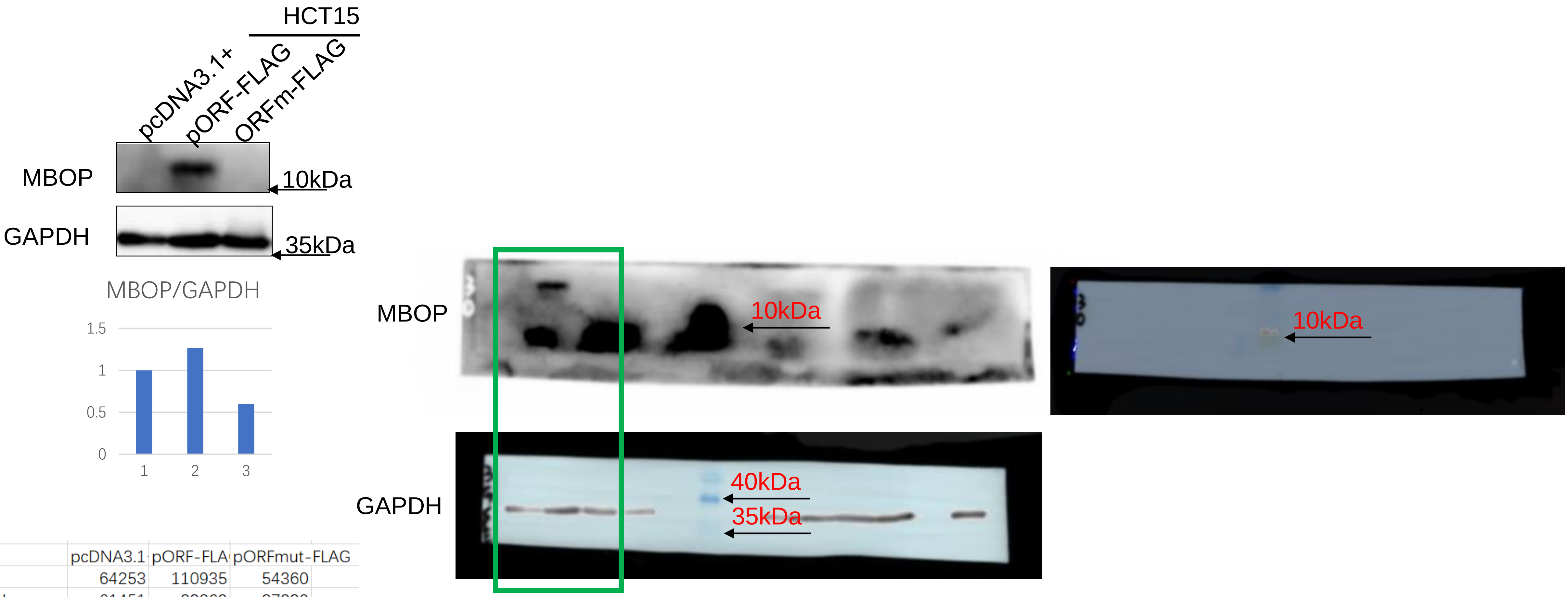

	pcDNA3.1	pORF-FLA	pORFmut-FLAG
MBOP	64253	110935	54360
GAPDH	61451	83869	87230
MBOP/GAPDH	1.045597	1.322718	0.62318
Ratio	1	1.265035	0.596004

Fig. 3E

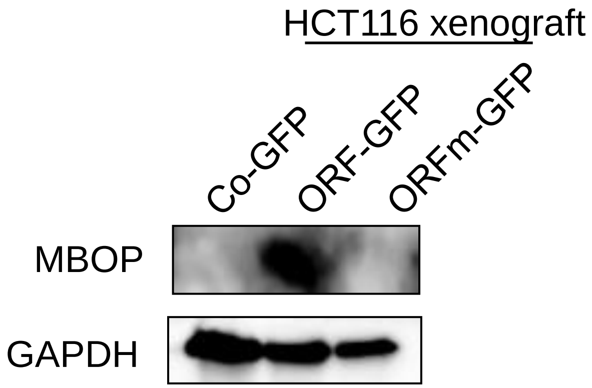

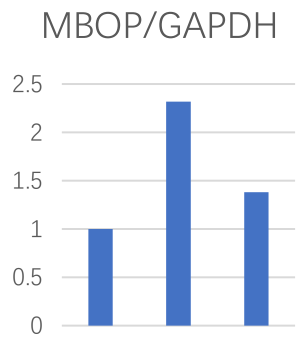

HCT116 xenograft	co-GFP	ORF-GFP	ORFm-GFP
MBOP	114523	214995	100451
GAPDH	141061	114226	89567
MBOP/GAPDH	0.811869	1.88219	1.121518
Ratio	1	2.318343	1.381403

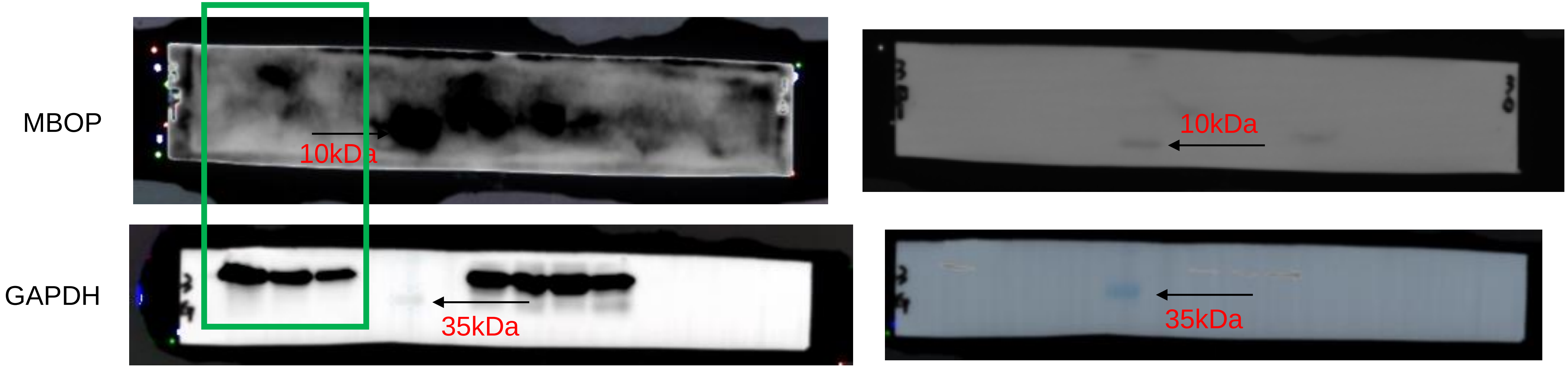

Fig. 4A

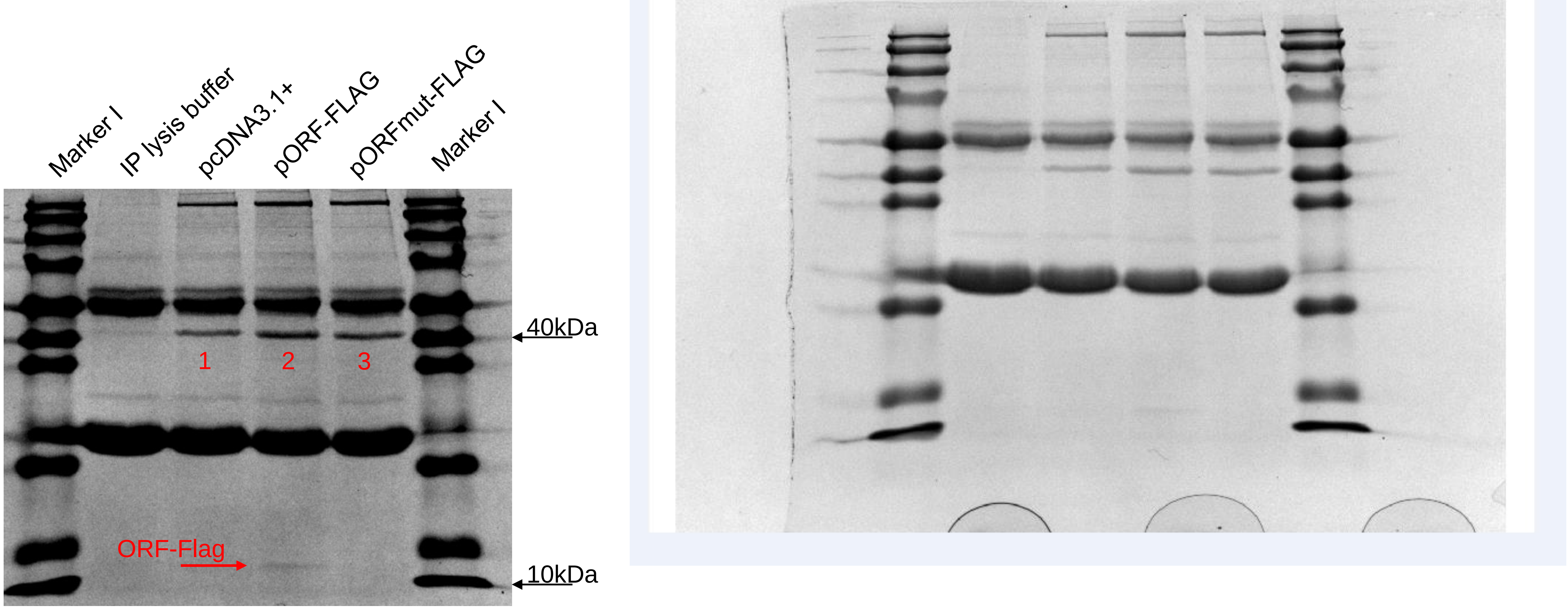

Marker I
IP lysis buffer
pcDNA3.1+
pORF-FLAG
pORFmut-FLAG
Marker I
40kDa
1
2
3
ORF-Flag
10kDa

Fig. 4E upper panel

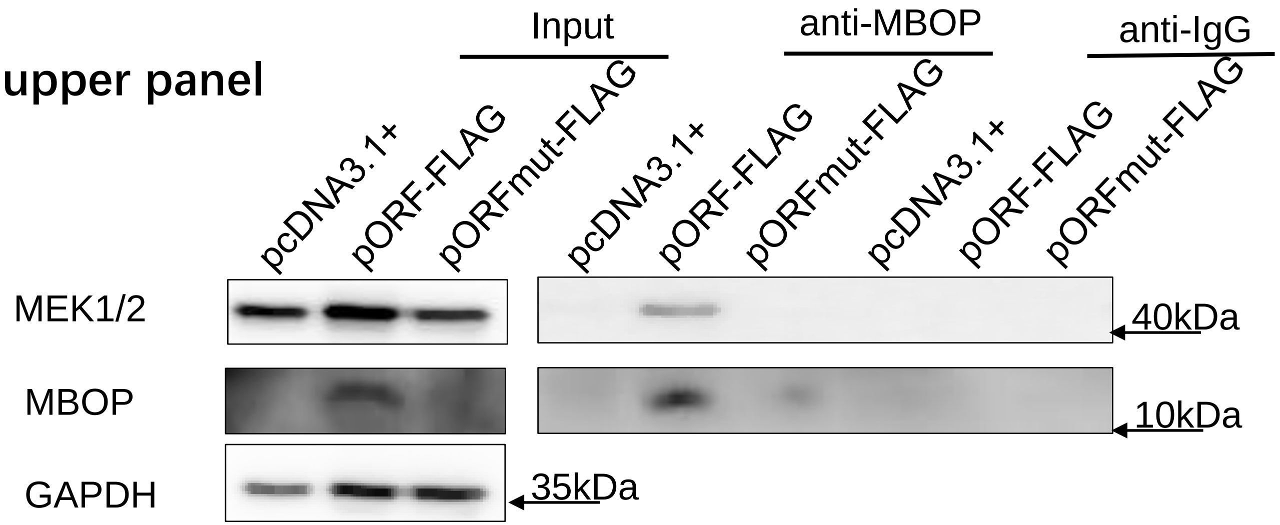

	pcDNA3.1	pORF-FLA	pORFm-FLAG
MEK1/2	40466	72761	49445
MBOP	2816	6321	3312
GAPDH	22592	34760	35716
MEK1/2 / GAPDH	1.791165	2.093239	1.384394
RATIO1	1	1.168647	0.772901
MBOP/GAPDH	0.124646	0.181847	0.092732
Ratio2	1	1.458908	0.74396

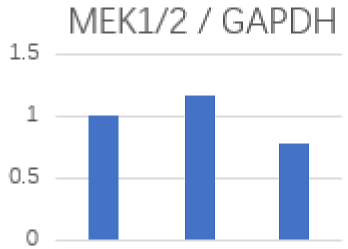

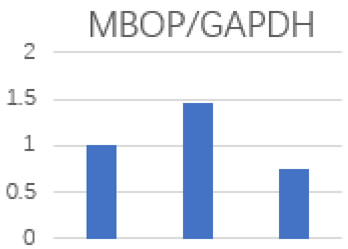

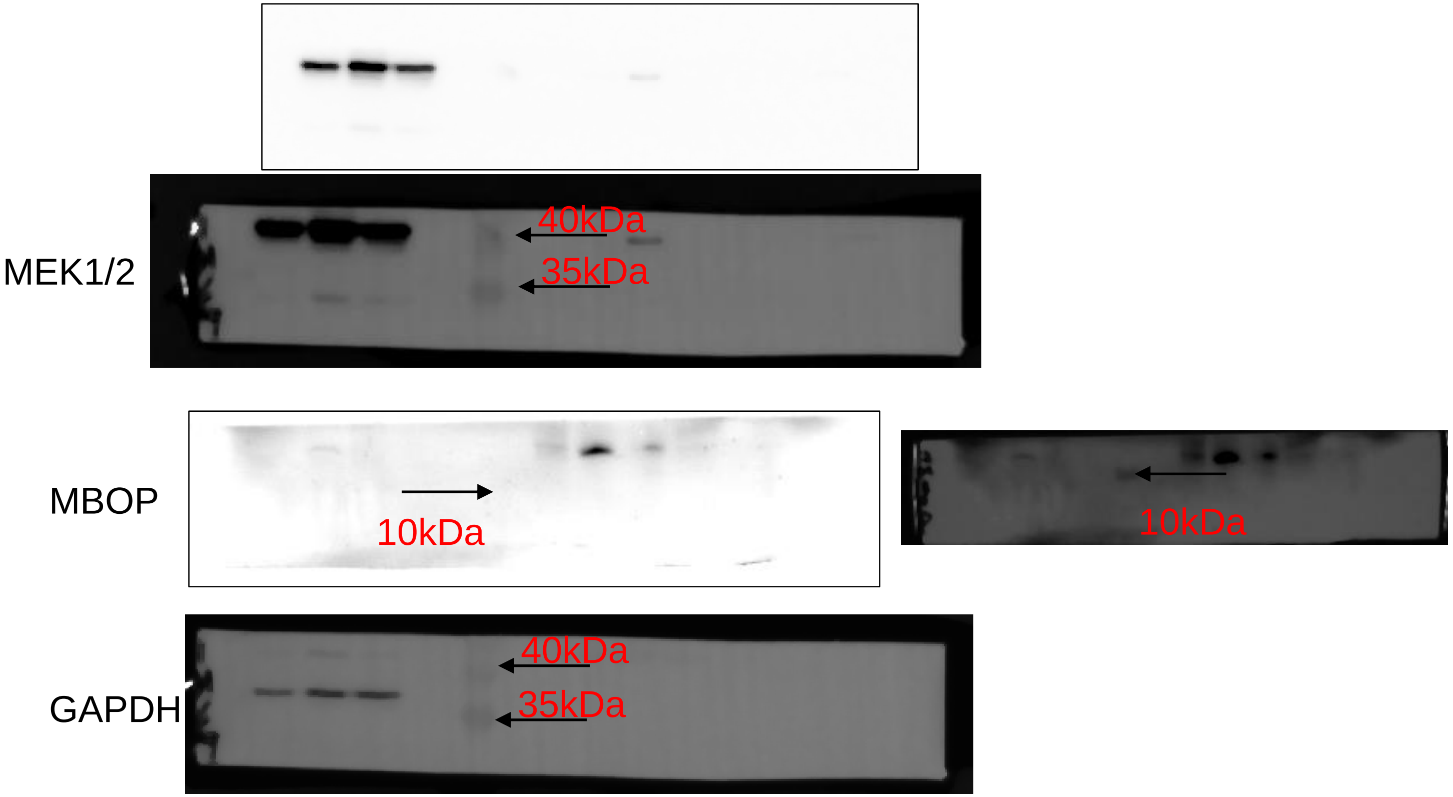

Fig. 4E lower panel

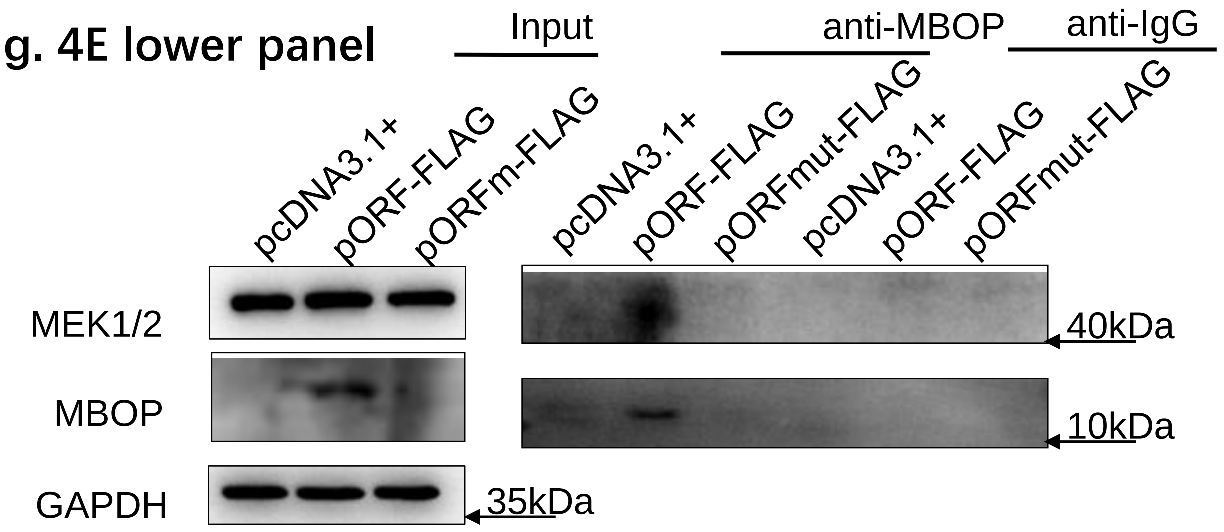

	pcDNA3.1	pORF-FLA	pORFm-FLAG
MEK1/2	1852834	2356890	1518417
MBOP	857482	1175259	687920
GAPDH	1524697	1565606	1457194
MEK1/2 / GAPDH	1.215215	1.505417	1.042014
RATIO1	1	1.238808	0.857474
MBOP/GAPDH	0.562395	0.750674	0.472085
Ratio2	1	1.33478	0.83942

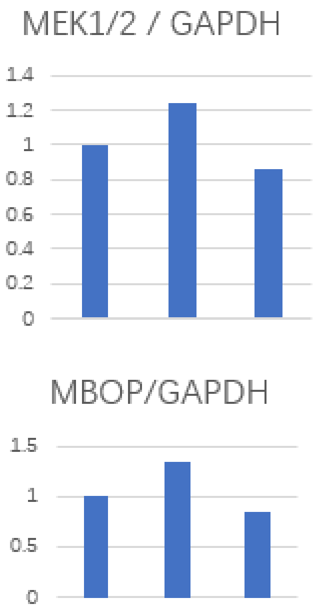

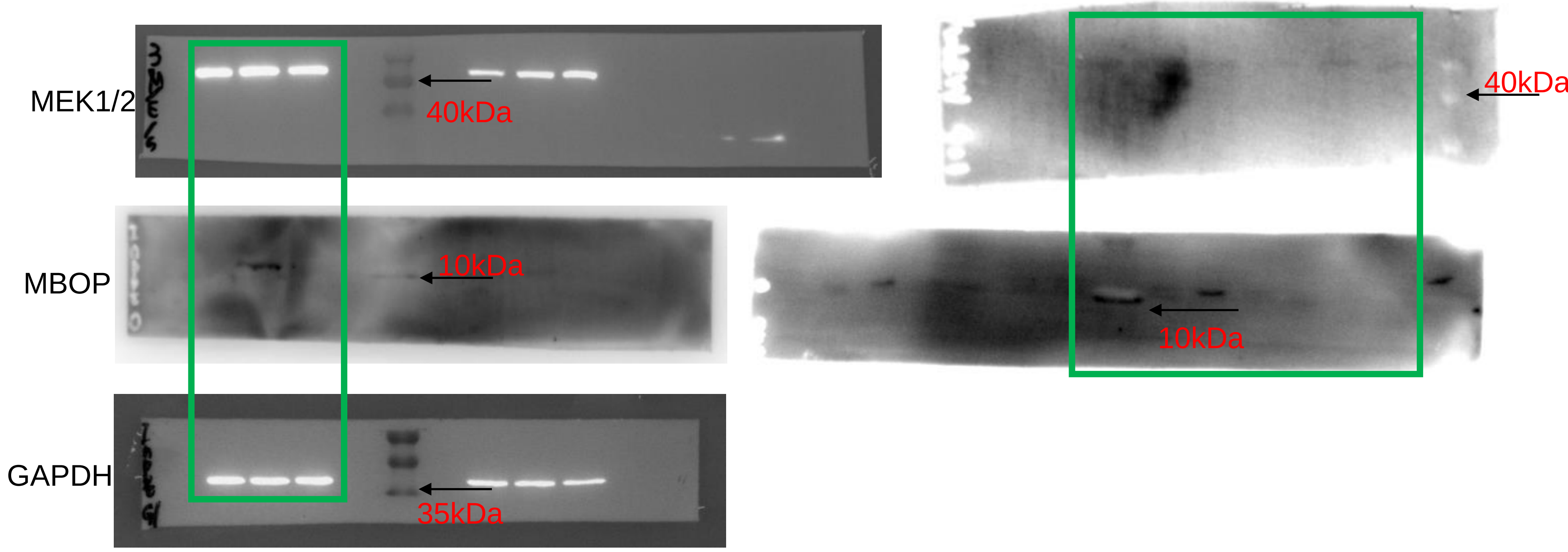

Fig. 4F upper panel

HCT116

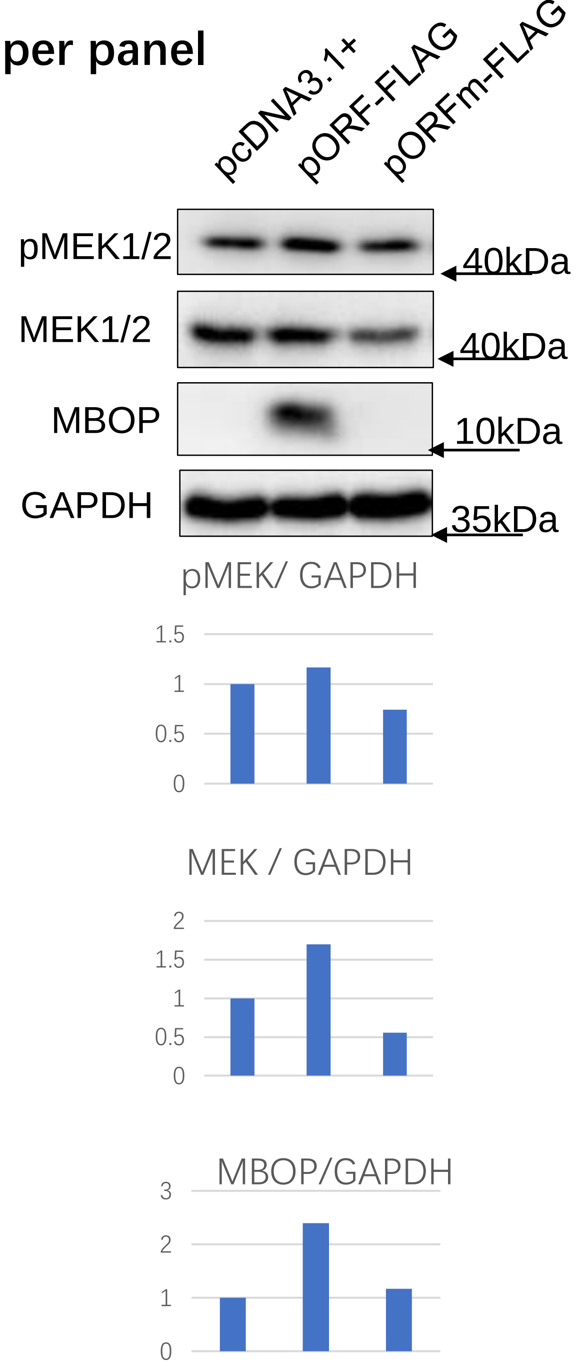

	pcDNA3.1	pORF-FLA	pORFm-FLAG
pMEK1/2	89599	89978	75321
MEK1/2	119814	175177	75334
MBOP	51023	105244	67504
GAPDH	157932	135946	179041
MBOP/GAPDH	0.323069	0.77416	0.377031
Ratio 1	1	2.396266	1.167028
pMEK1/2 / GAPDH	0.567326	0.661866	0.420691
Ratio 2	1	1.16664	0.741533
MEK1/2 / GAPDH	0.758643	1.288578	0.420764
Ratio 3	1	1.69853	0.554627

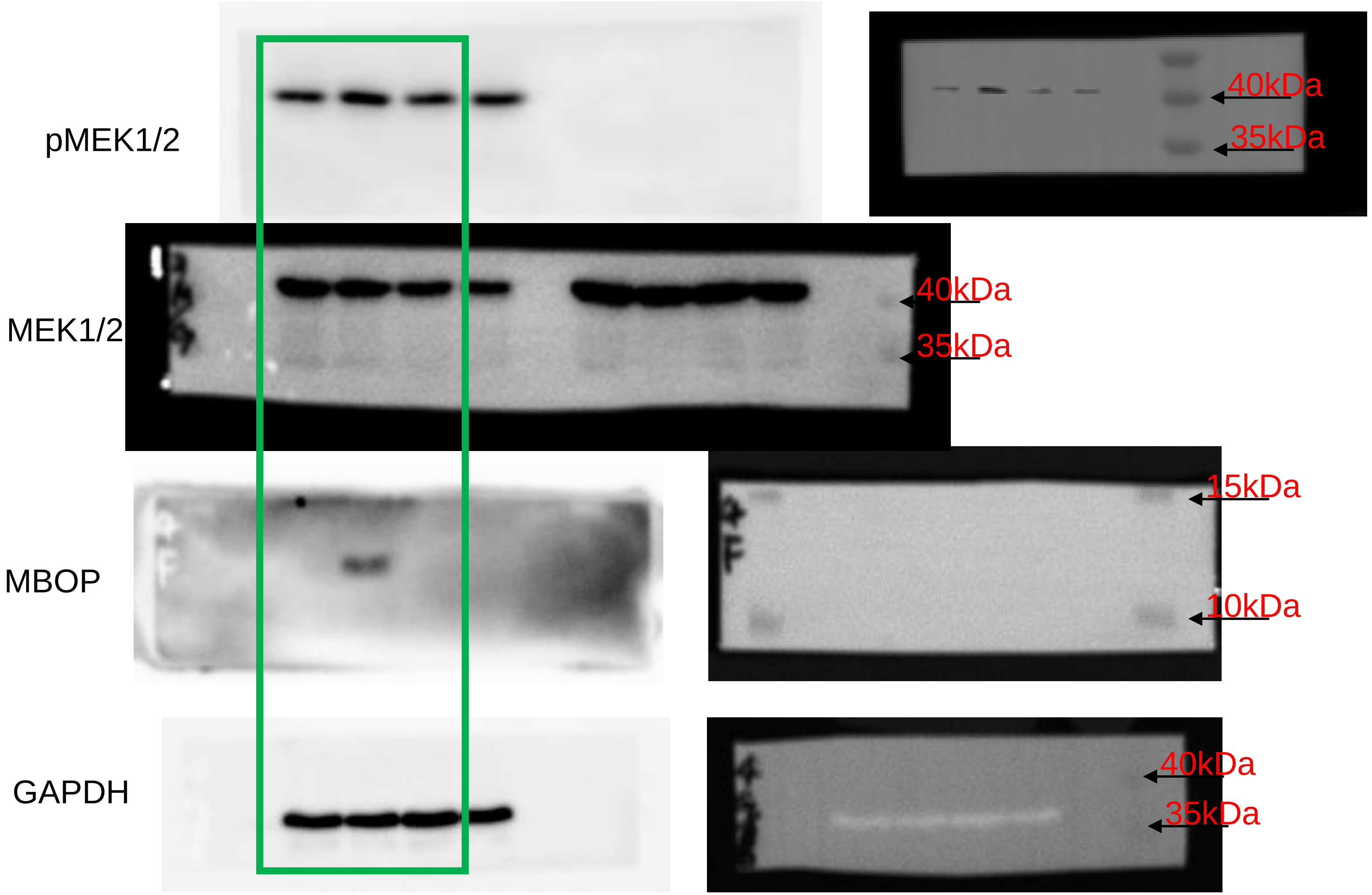

Fig. 4F lower panel

	pcDNA3.1	pORF-FLA	pORFm-FLAG
pMEK1/2	150238	288385	147839
MEK1/2	37281	59978	25644
MBOP	57120	147328	60979
GAPDH	667673	606084	697396
MBOP/GAPDH	0.085551	0.243082	0.087438
Ratio 1	1	2.841372	1.02206
pMEK1/2 / GAPDI	0.225017	0.475817	0.211987
Ratio 2	1	2.114579	0.942093
MEK1/2 / GAPDH	0.055837	0.09896	0.036771
Ratio 3	1	1.772293	0.658541

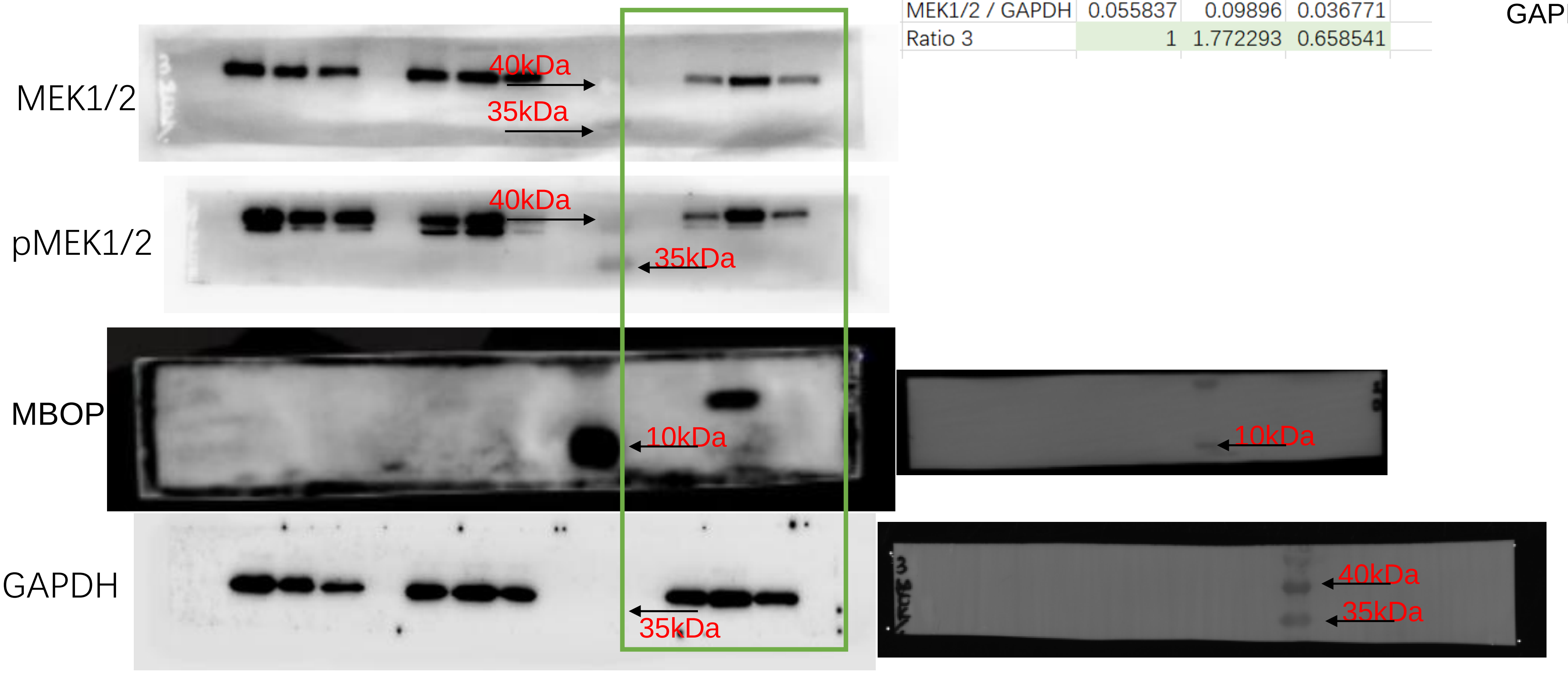

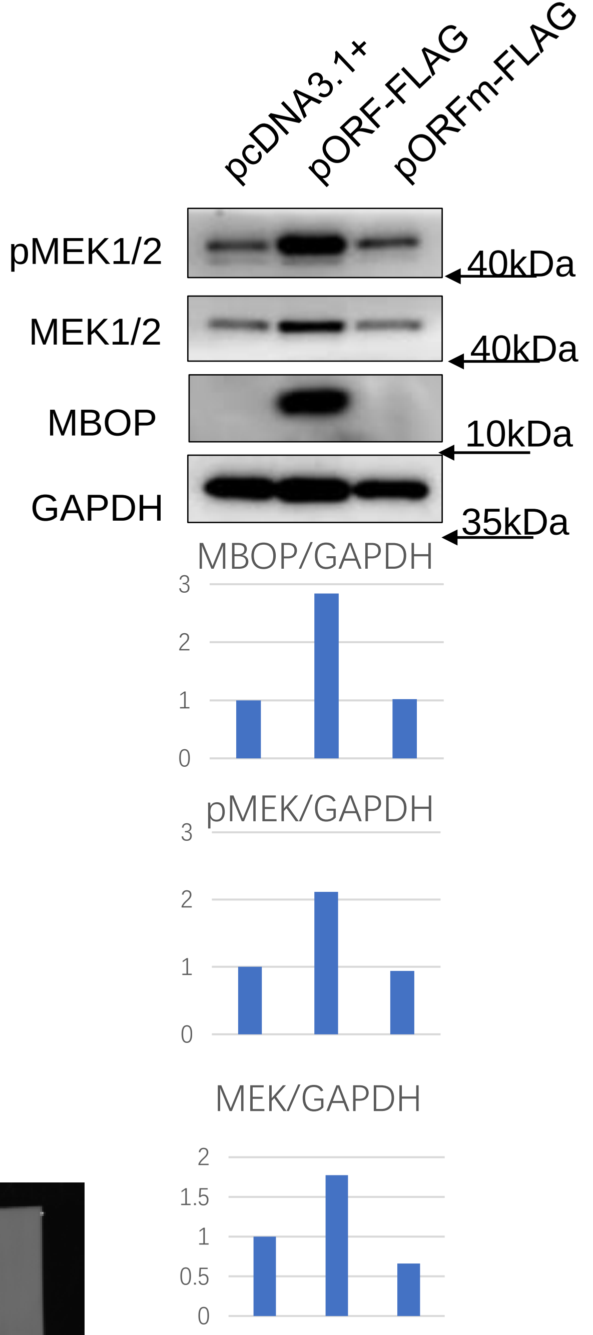

Fig. 5A LETF panel

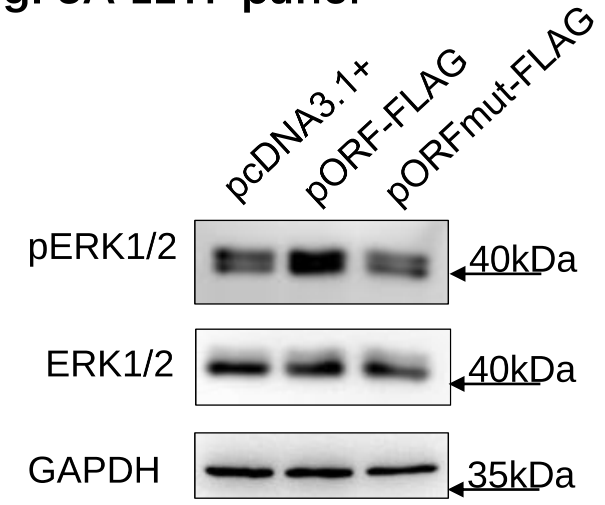

	pcDNA3.1	pORF-FLA	pORFm-FLAG
pERK1/2	55923	68138	53691
ERK1/2	43578	46991	45522
GAPDH	432256	442802	415193
pERK1/2 to GAPD	0.129375	0.153879	0.129316
Ratio1	1	1.189407	0.999544
ERK1/2 to GAPDH	0.100815	0.106122	0.109641
Ratio2	1	1.052638	1.08754

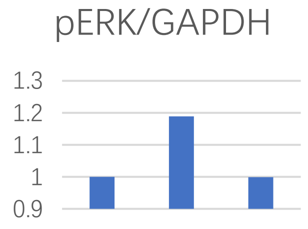

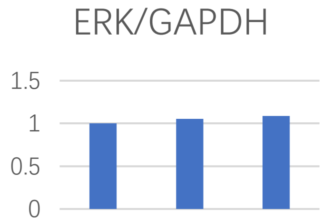

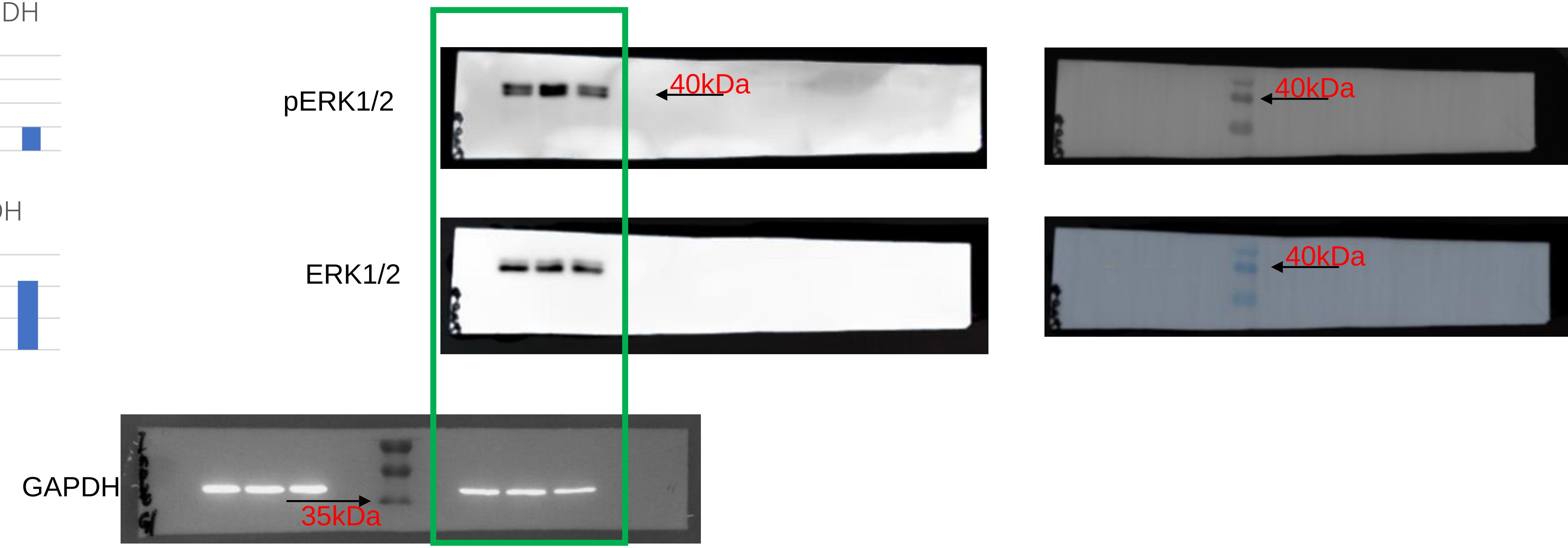

Fig. 5A RIGHT panel

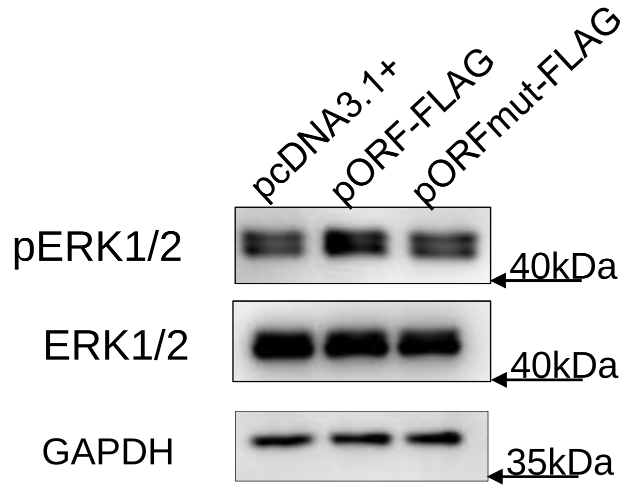

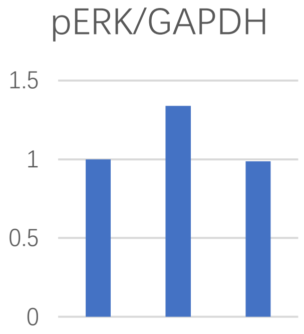

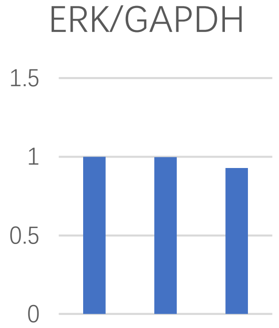

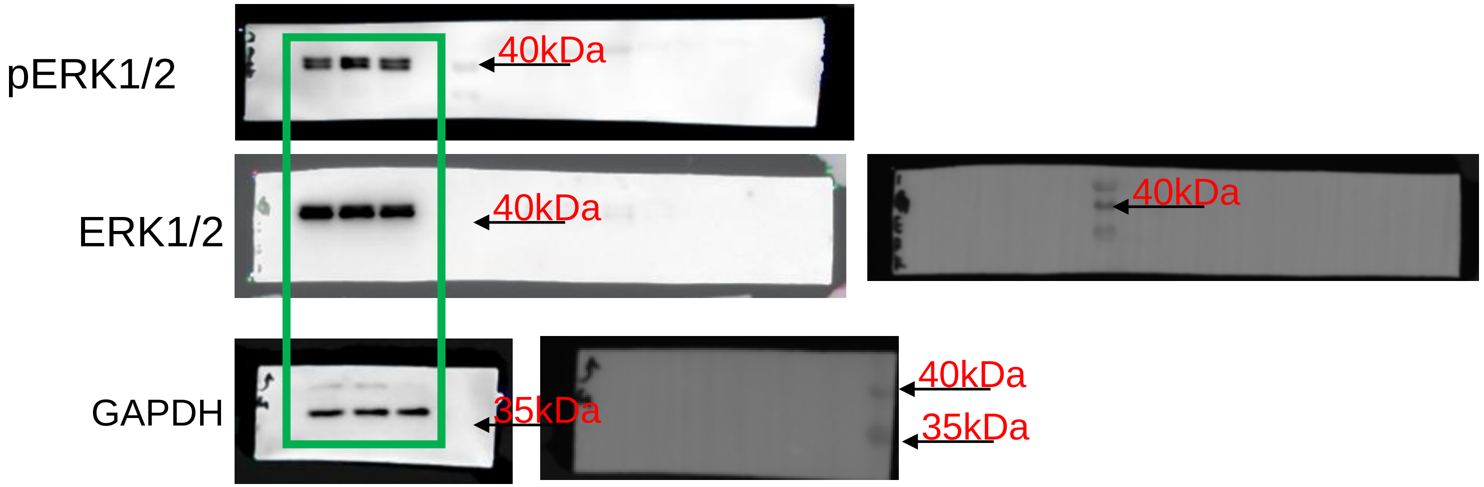

	pcDNA3.1	pORF-FLA	pORFm-FLAG
pERK1/2	55300	74662	54046
ERK1/2	64535	64996	59350
GAPDH	207750	209700	205711
pERK1/2 to GAPD	0.266185	0.356042	0.262728
Ratio1	1	1.337572	0.987011
ERK1/2 to GAPDH	0.310638	0.309948	0.288512
Ratio2	1	0.997778	0.928772

Fig. 5B LETF panel

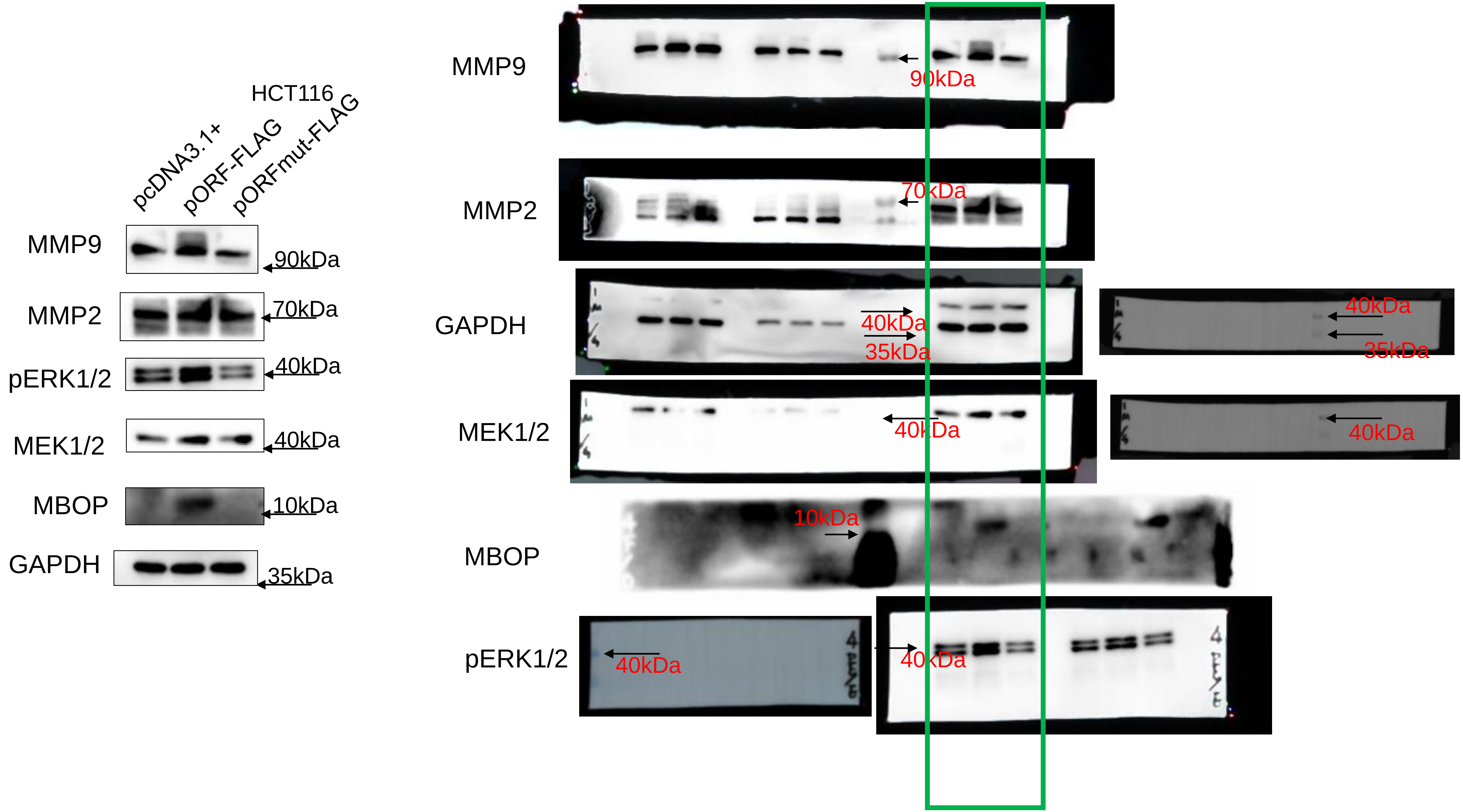

Fig. 5B RIGHT panel

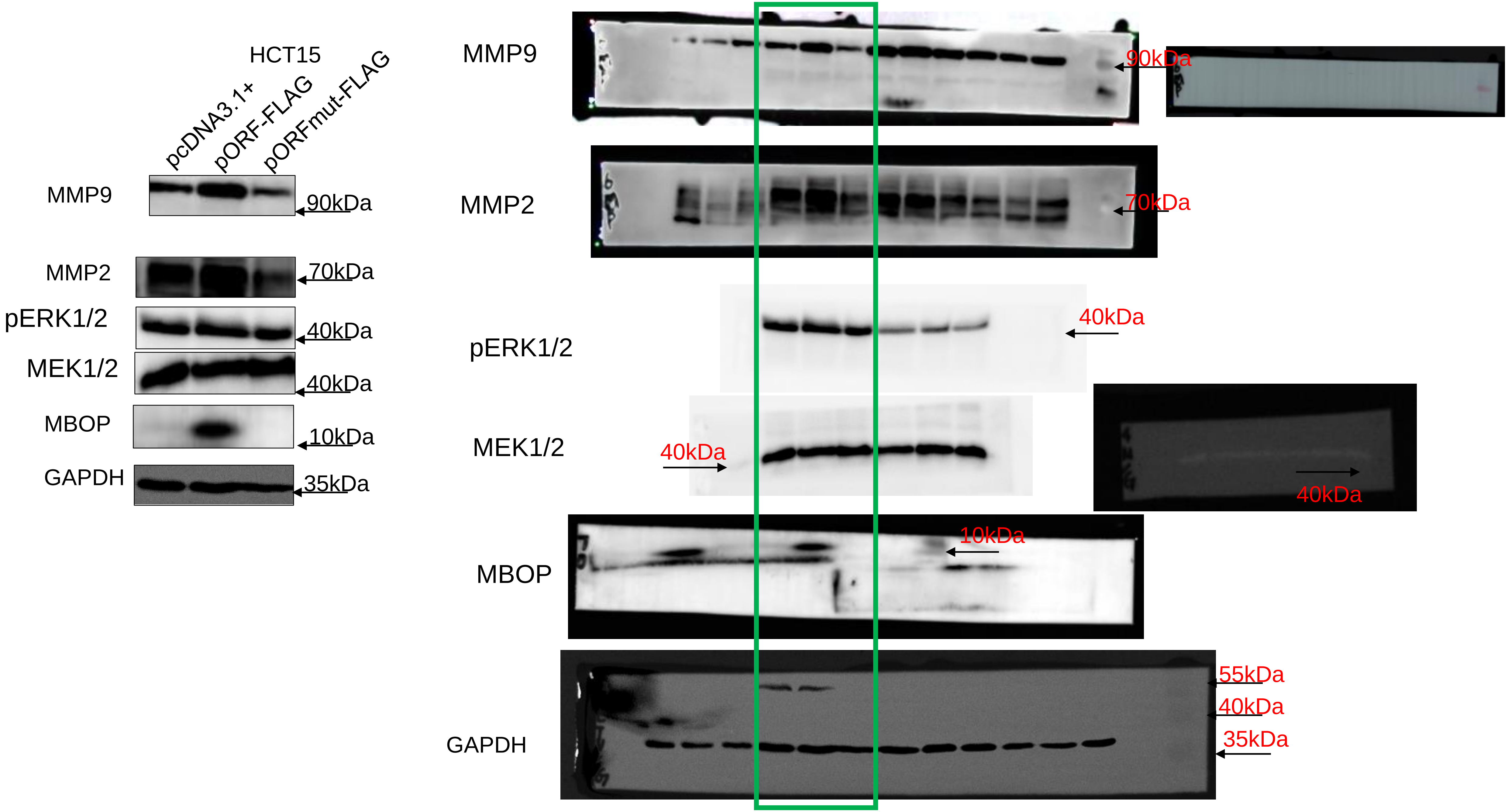

Fig. 5B LETF panel

HCT116	pcDNA3.1	pORF-FLA	pORFmut-FLAG
MMP9	97991	160487	90516
MMP2	91827	124863	94603
pERK	76516	90722	49964
MEK	23041	36633	28007
MBOP	115718	166193	106193
GAPDH	93618	101434	108044
MMP9/G	1.046711	1.582182	0.83777
Ratio1	1	1.511574	0.800383
MMP2/G	0.980869	1.230978	0.875597
Ratio2	1	1.254987	0.892675
pERK/G	0.817321	0.894394	0.462441
Ratio3	1	1.094299	0.565801
MEK/G	0.246117	0.361151	0.259218
Ratio4	1	1.467395	1.053232
MBOP/G	1.236066	1.638435	0.982868
Ratio5	1	1.325524	0.795158

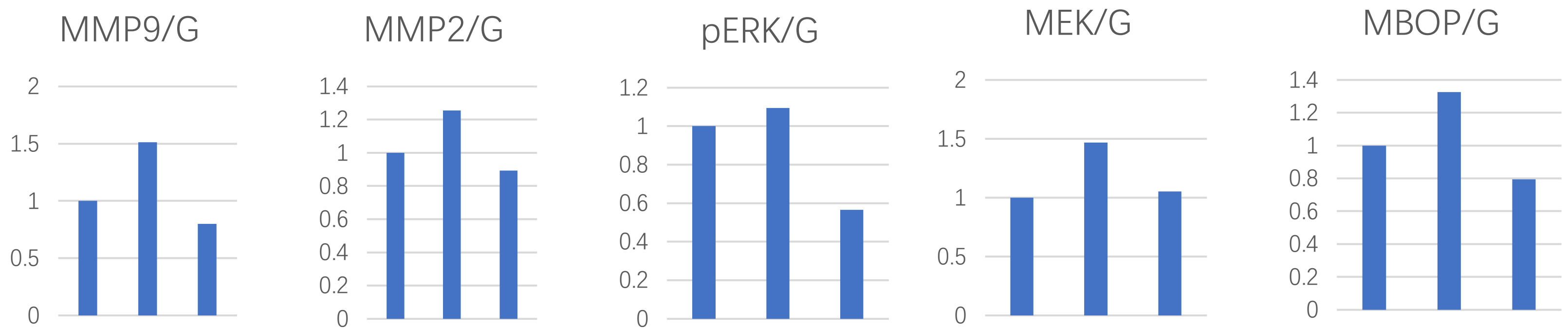

Fig. 5B RIGHT panel

HCT15	pcDNA3.1	pORF-FLA	pORFmut-FLAG
MMP9	55220	84879	42808
MMP2	118646	166067	102514
pERK	276341	348763	219093
MEK	319979	369248	316648
MBOP	125205	333368	122178
GAPDH	321706	328406	318262
MMP9/G	0.171647	0.258458	0.134506
Ratio1	1	1.505747	0.783615
MMP2/G	0.368803	0.505676	0.322106
Ratio2	1	1.371129	0.873382
pERK/G	0.858986	1.061987	0.688405
Ratio3	1	1.236326	0.801415
MEK/G	0.994632	1.124364	0.994929
Ratio4	1	1.130433	1.000299
MBOP/G	0.389191	1.015109	0.383891
Ratio5	1	2.608257	0.986383

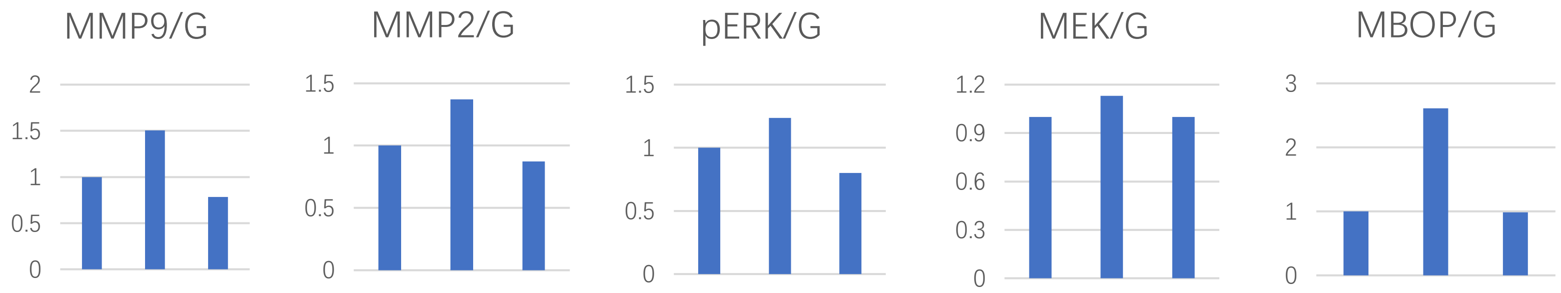

Fig. 5C

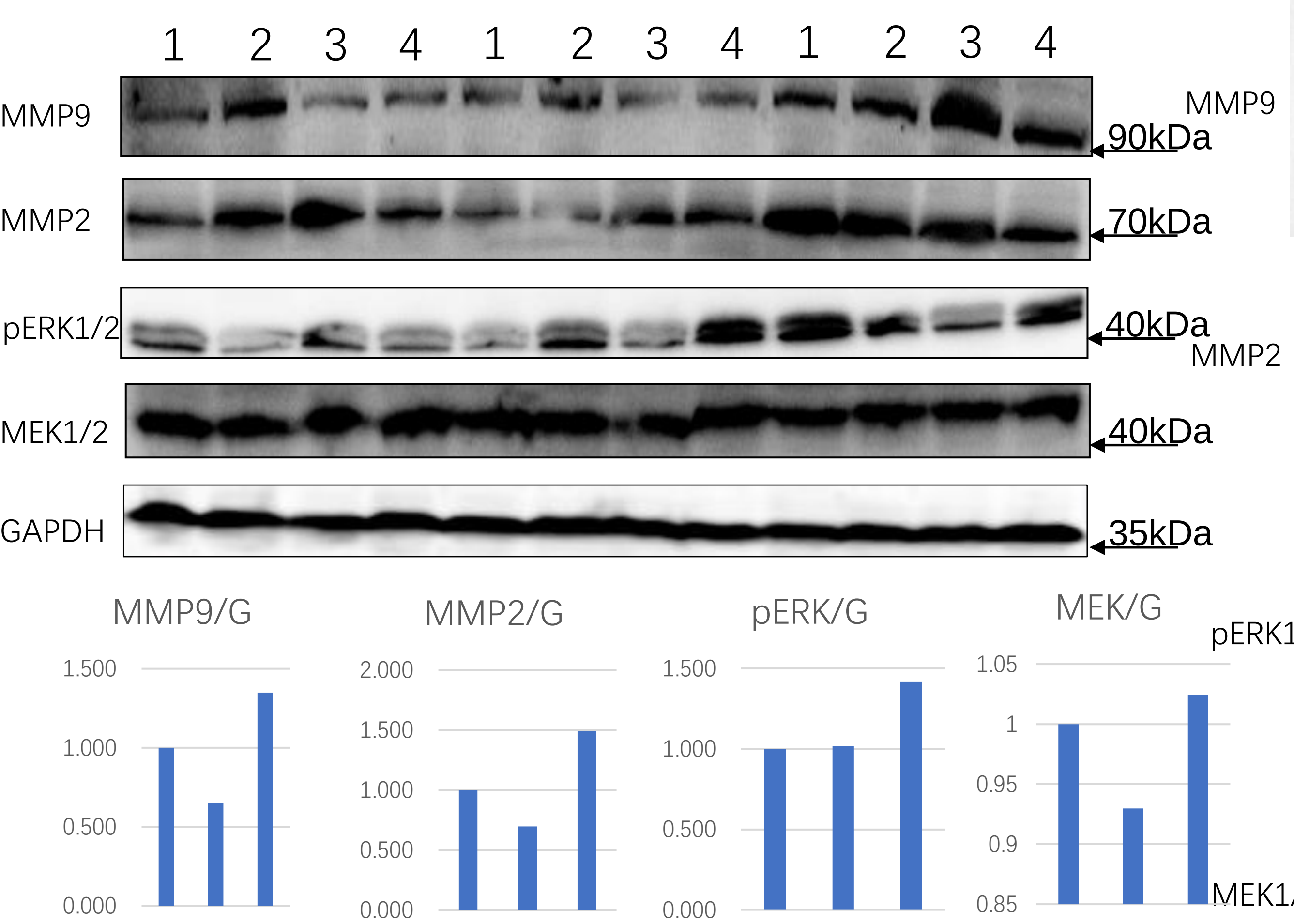

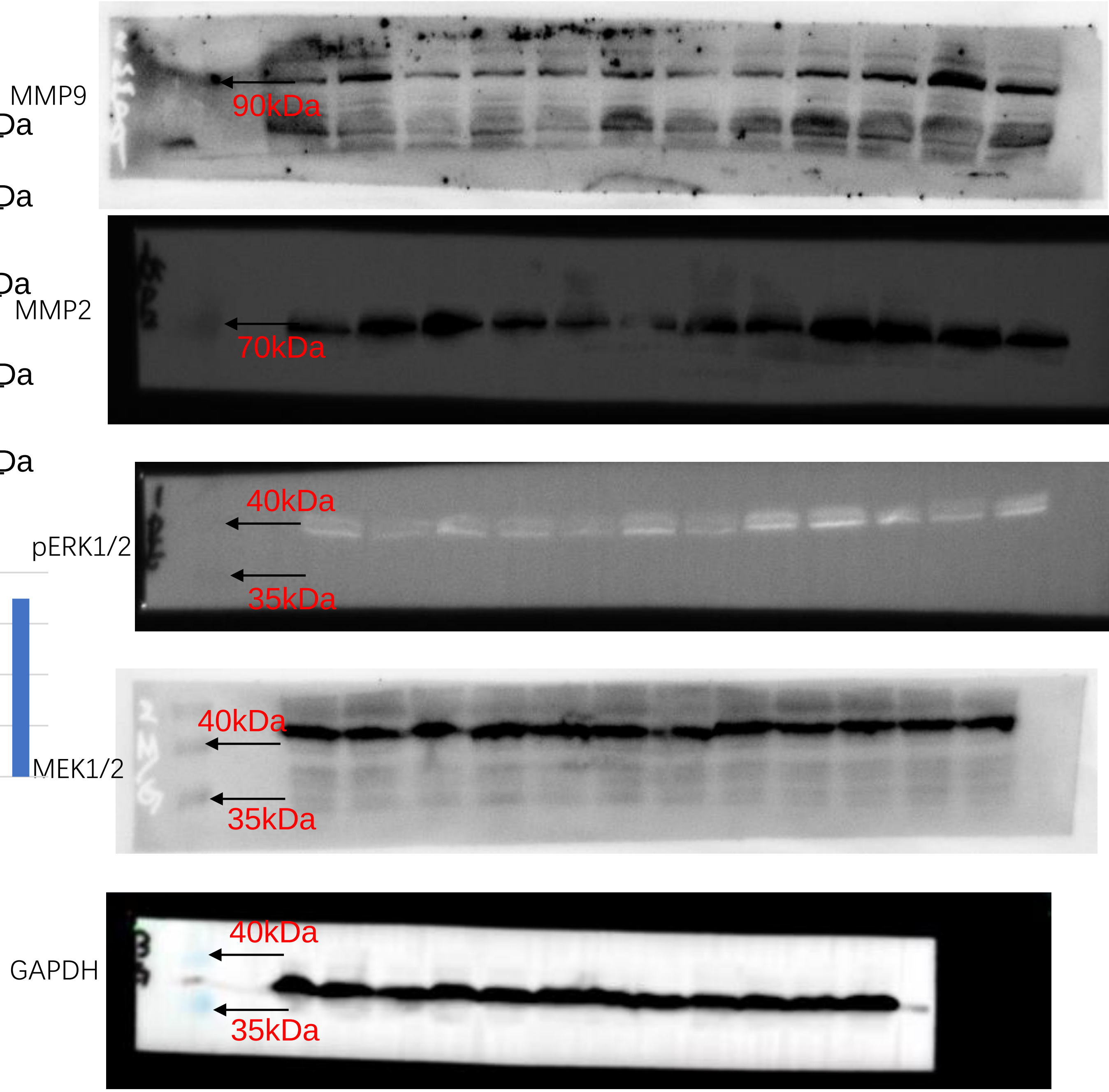

	co-GFP	ORFm-GFP	ORF-GFP
MMP9	953662	636150	1214527
MMP2	493569	354188	694467
pERK1/2	840178	879569	1125396
MEK1/2	983365	940154	951449
GAPDH	382707	393520	361475
MMP9/G	2.4918854	1.61656333	3.3599198
Ratio1	1.000	0.649	1.348
MMP2/G	1.290	0.900	1.921
Ratio2	1.000	0.698	1.490
pERK/G	2.195	2.235	3.113
Ratio3	1.000	1.018	1.418
MEK/G	2.569	2.389	2.632
Ratio4	1	0.92978781	1.0243748

Fig. 5D

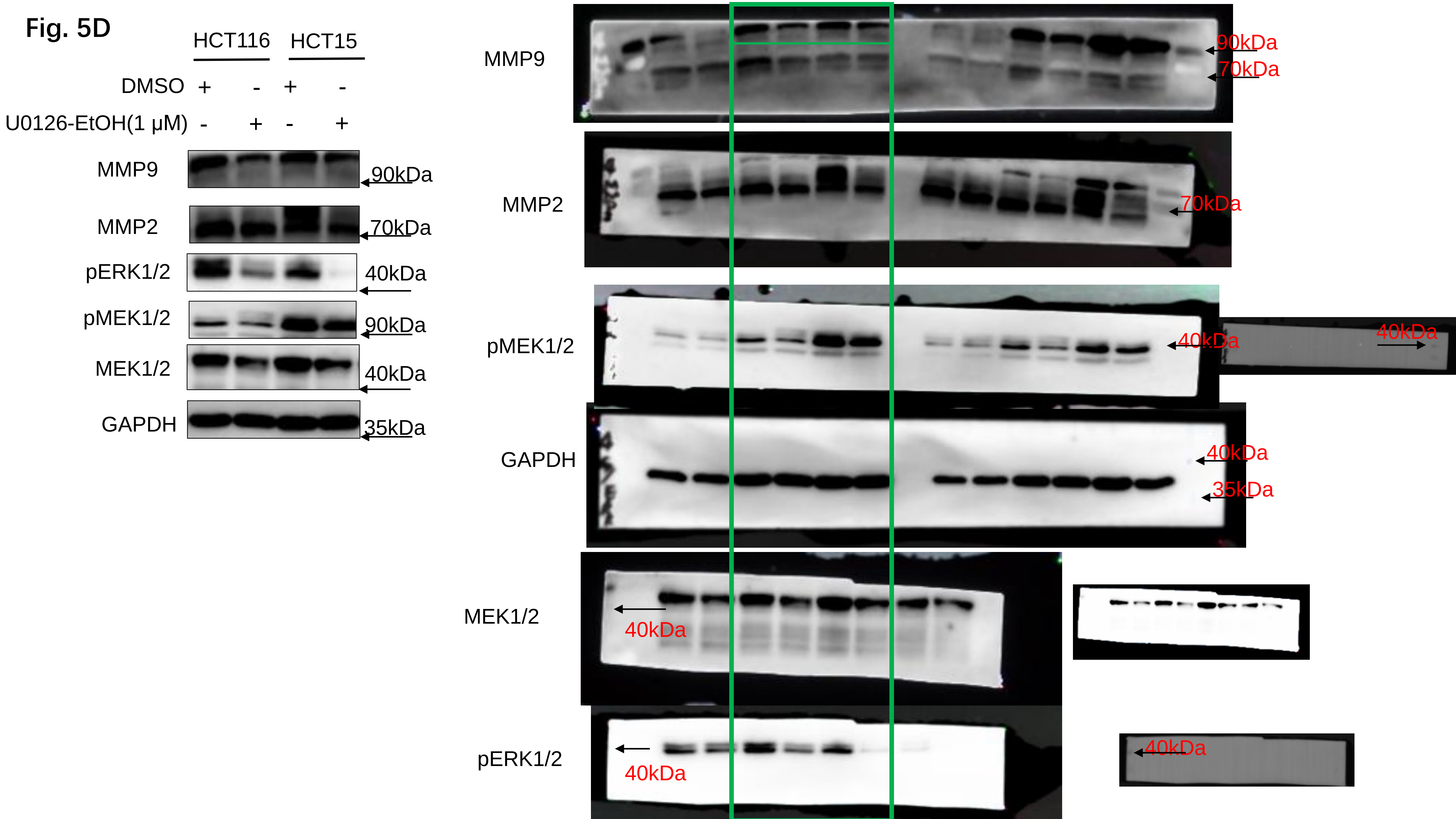
HCT116
HCT15
DMSO + - + -
U0126-EtOH(1 μM) - + - +
MMP9
90kDa
MMP2
70kDa
pERK1/2
40kDa
pMEK1/2
90kDa
MEK1/2
40kDa
GAPDH
35kDa
MMP9
90kDa
70kDa
MMP2
70kDa
pMEK1/2
40kDa
40kDa
GAPDH
40kDa
35kDa
MEK1/2
40kDa
pERK1/2
40kDa
40kDa

Fig. 5D

	DMSO	U0126-EtOH	DMSO	U0126-EtOH
MMP9	86236	46614	73919	53710
MMP2	66910	58407	75857	59973
pERK	75989	43810	60401	11723
pMEK	79357	67006	149779	137147
MEK	74004	52150	93797	64529
GAPDH	137307	134766	121209	122094
MMP9/GAPDH	0.6280525	0.34588843	0.6098475	0.439907
Ratio1	1	0.55073174	1	0.721339
MMP2/GAPDH	0.4873022	0.43339566	0.6258364	0.491203
Ratio2	1	0.88937765	1	0.784875
pERK/GAPDH	0.5534241	0.32508199	0.4983211	0.096016
Ratio3	1	0.58740125	1	0.192679
pMEK/GAPDH	0.5779531	0.49720256	1.2357086	1.12329
Ratio4	1	0.86028191	1	0.909025
MEK/GAPDH	0.5389674	0.38696704	0.7738452	0.528519
Ratio5	1	0.71797853	1	0.682978

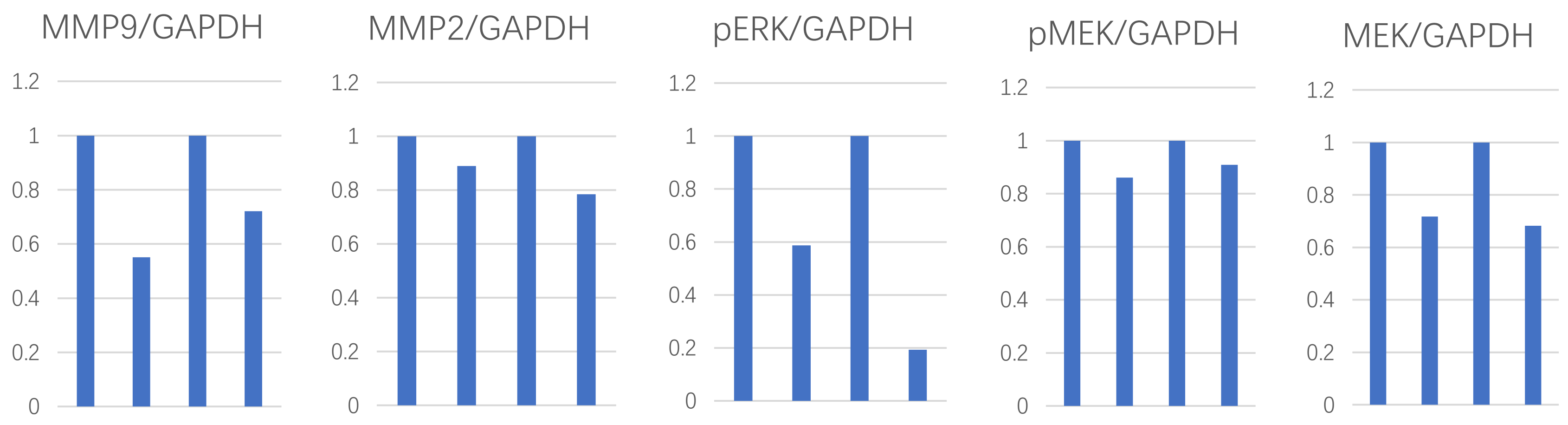

Fig. 5E-HCT116

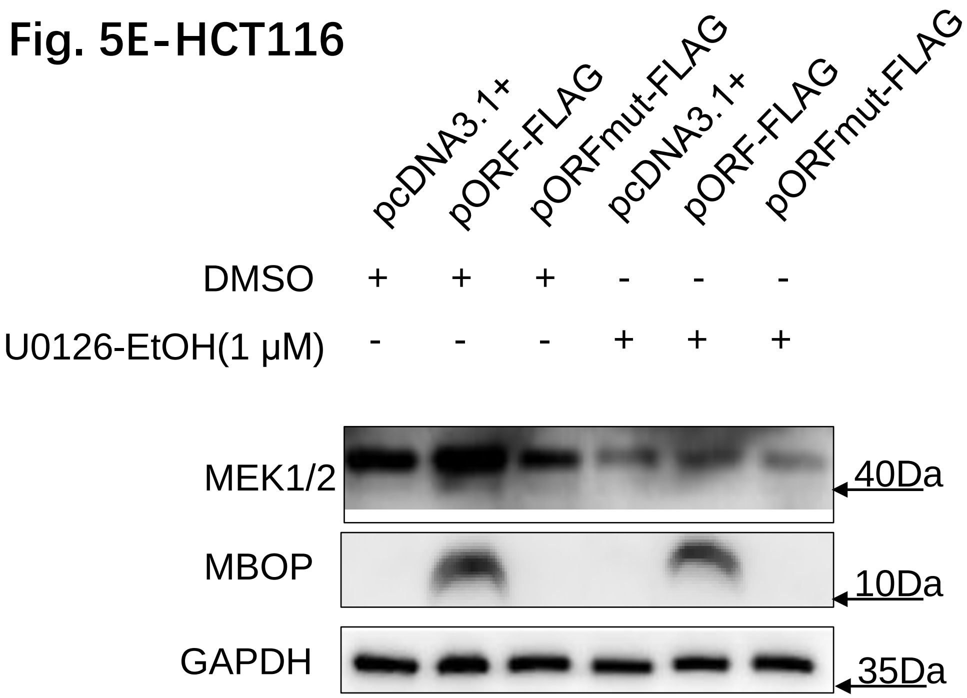

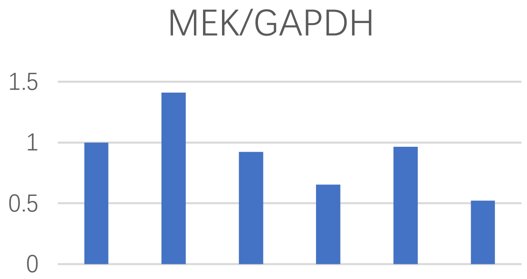

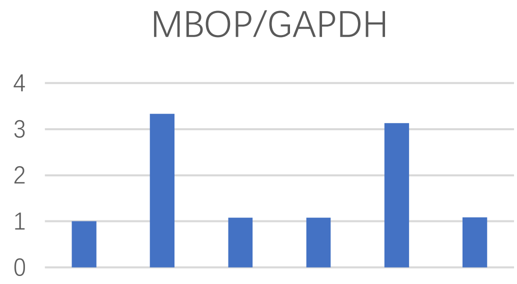

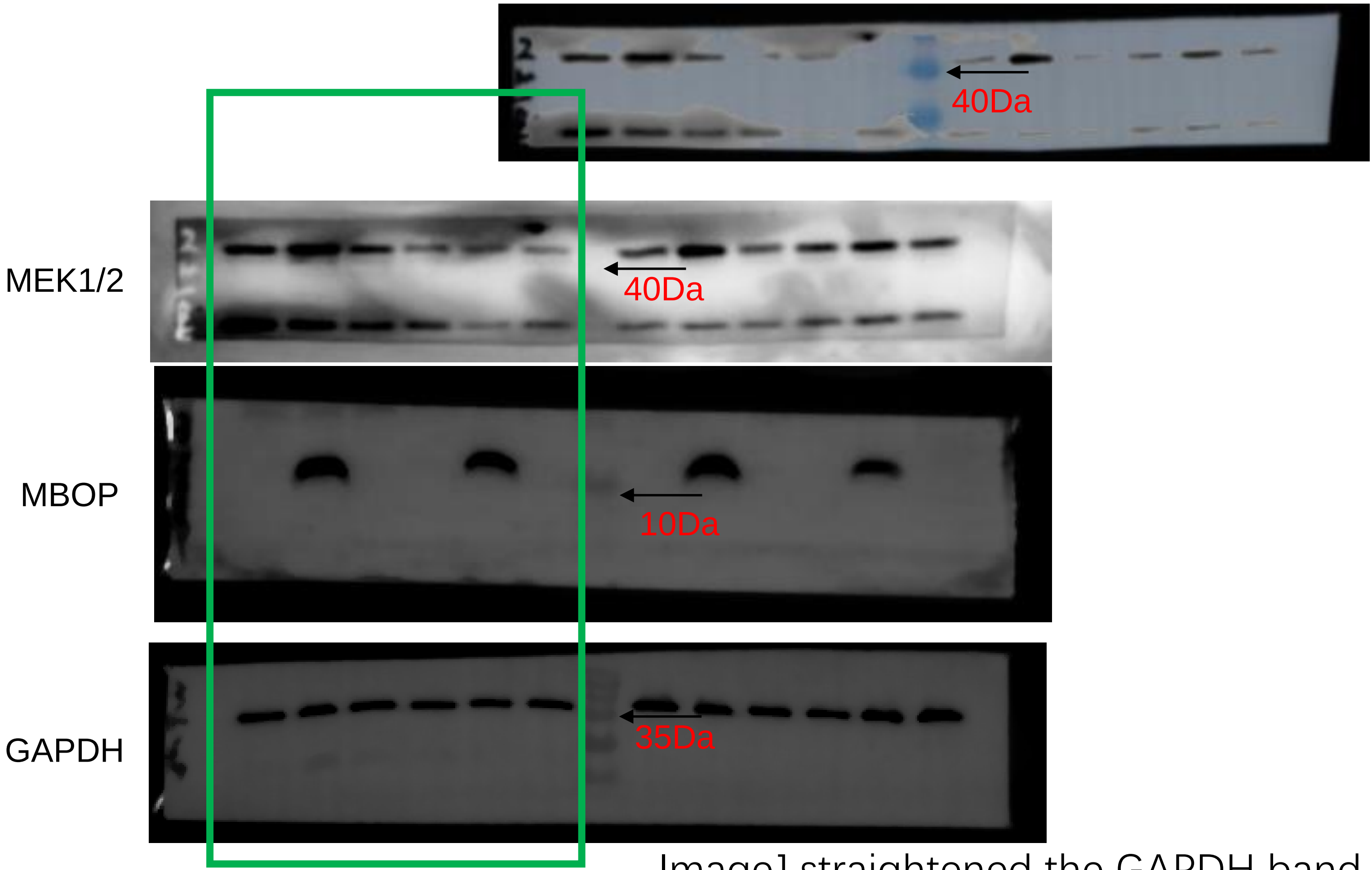

ImageJ straightened the GAPDH band

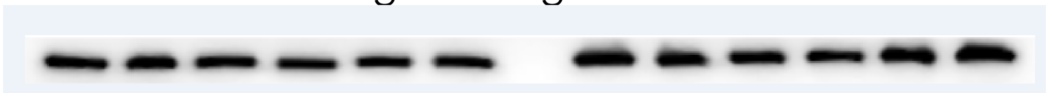

	pcDNA3.1+ DMSO	pORF-FLAG DMSO	pORFmut-FL DMSO	pcDNA3.1 U0126-Et(	pORF-FLA U0126-Et(	pORFmut-FLAG U0126-EtOH
MEK1/2	41706	50896	33290	22815	32337	18957
MBOP	27935	80604	26030	29024	71918	26550
GAPDH	45570	39456	39387	38209	36624	39729
MEK/GAPDH	0.9152074	1.28994323	0.8452027	0.597111	0.882946	0.477158
Ratio1	1	1.40945458	0.9235095	0.652432	0.964749	0.521366
MBOP/GAPDH	0.6130129	2.04288321	0.660878	0.759612	1.963685	0.668278
Ratio2	1	3.33252865	1.0780816	1	2.585117	0.879762

Fig. S4B-HCT15

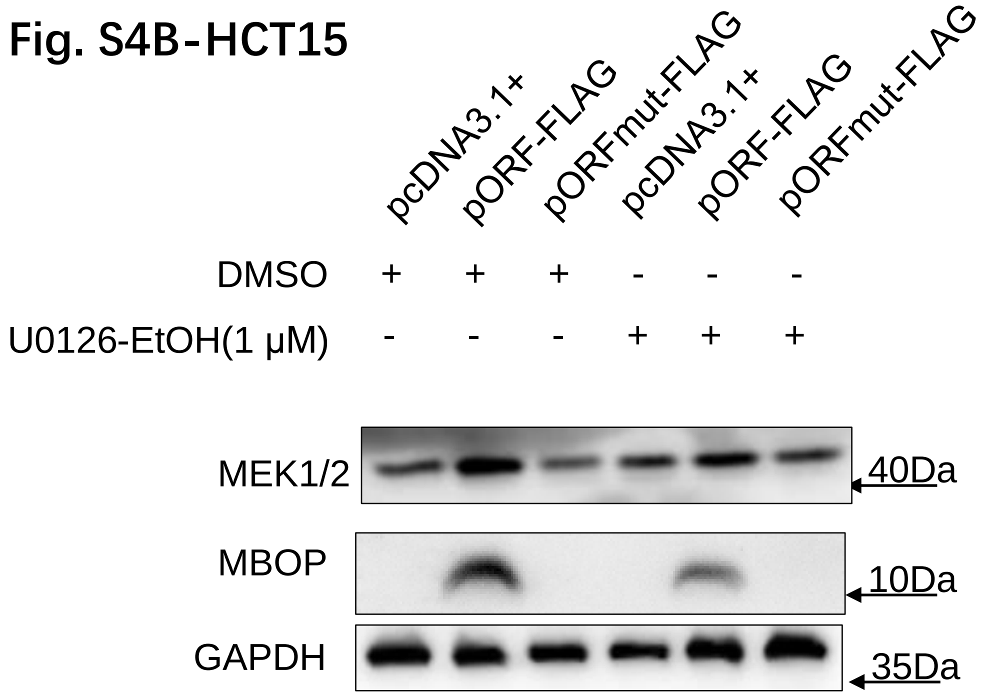

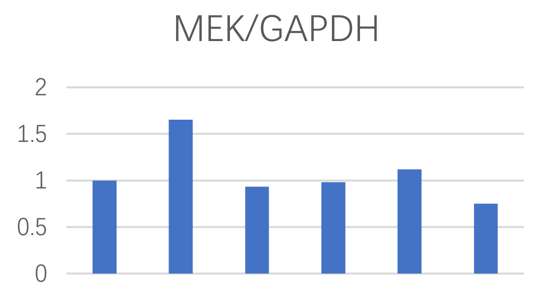

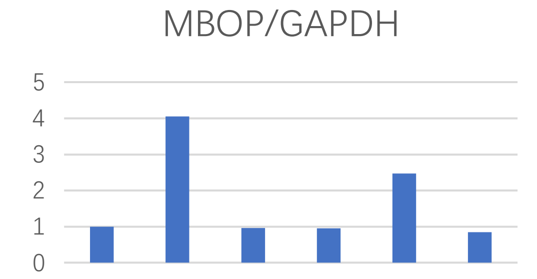

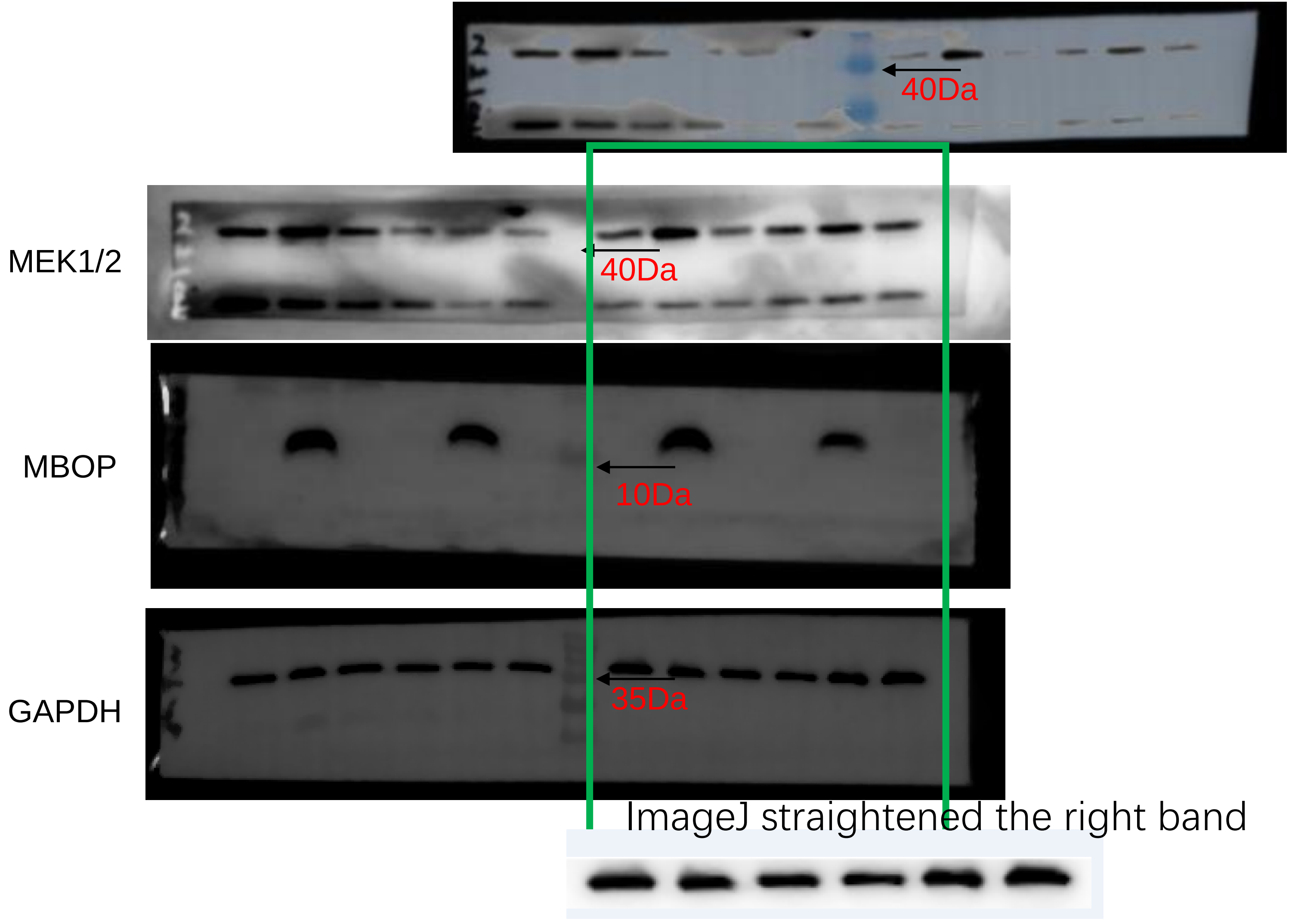

	pcDNA3.1+ DMSO	pORF-FLAG DMSO	pORFmut-Fl DMSO	pcDNA3.1 U0126-Et(	pORF-FLA U0126-Et(	pORFmut-FLAG U0126-EtOH
MEK1/2	59893	81671	50249	45836	60613	45378
MBOP	23284	77923	20102	17296	51925	19913
GAPDH	53075	43787	47618	41365	47920	53465
MEK/GAPDH	1.1284597	1.8651883	1.0552522	1.108087	1.264879	0.848742
Ratio1	1	1.65286209	0.9351262	0.981946	1.12089	0.752124
MBOP/GAPDH	0.4387	1.77959212	0.4221513	0.418131	1.083577	0.372449
Ratio2	1	4.05651312	0.9622779	0.953114	2.469972	0.848984

Fig. S4D

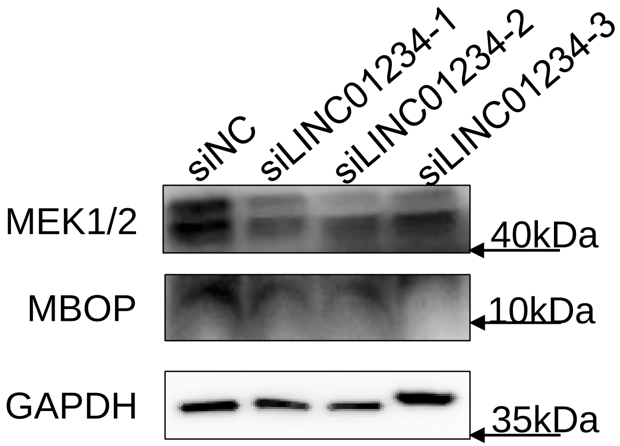

	siNC	siLINC01234-	siLINC01234	siLINC01234-3
MEK1/2	63203	31386	34907	46555
MBOP	148609	113823	98009	86266
GAPDH	45307	39118	35208	51327
MEK/GAPDH	1.3949942	0.80234163	0.9914508	0.907027
RATIO1	1	0.5751577	0.7107204	0.650202
MBOP/GAPDH	3.280045	2.90973465	2.7837139	1.680714
RATIO2	1	0.88710204	0.8486816	0.512406

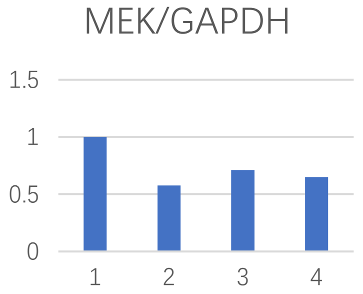

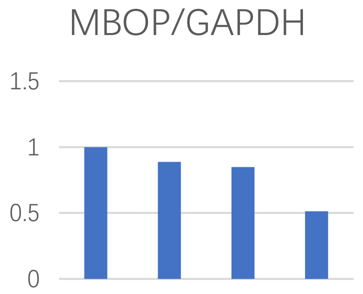

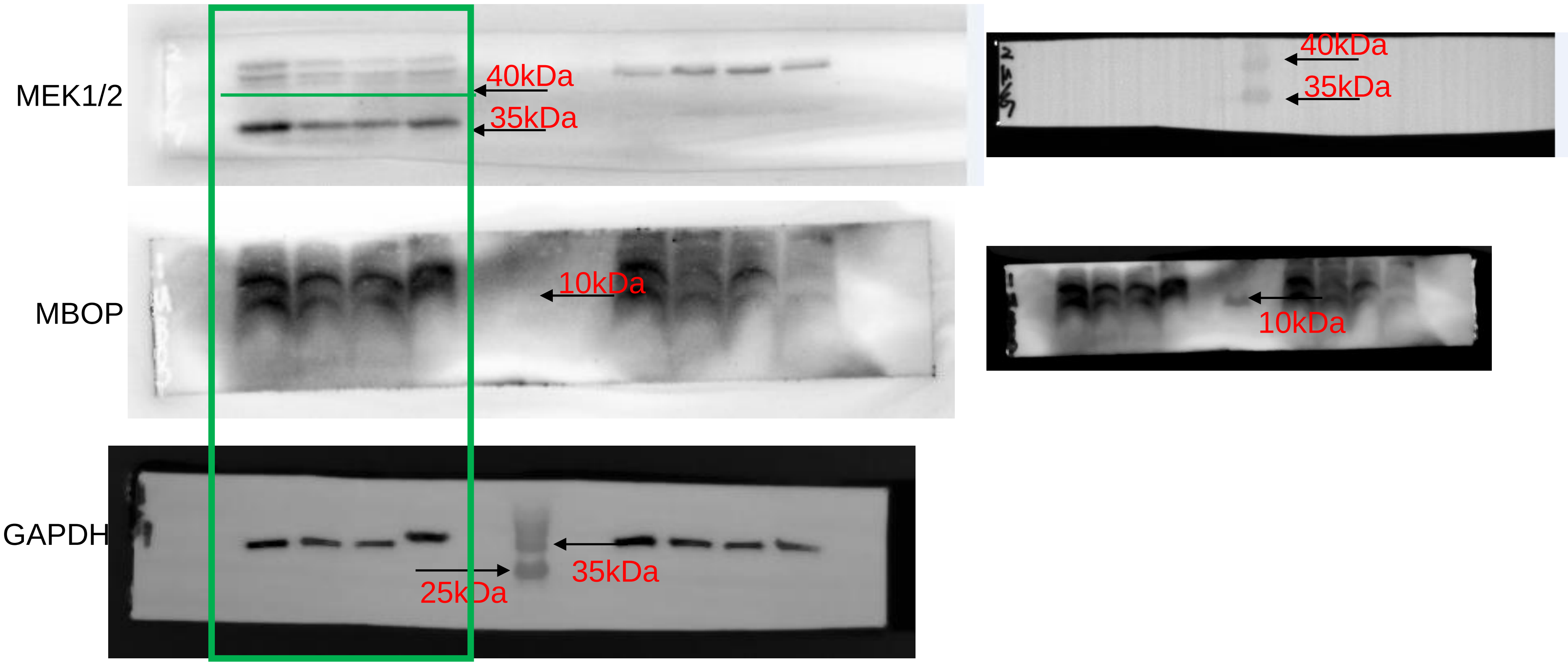

Fig. S4E

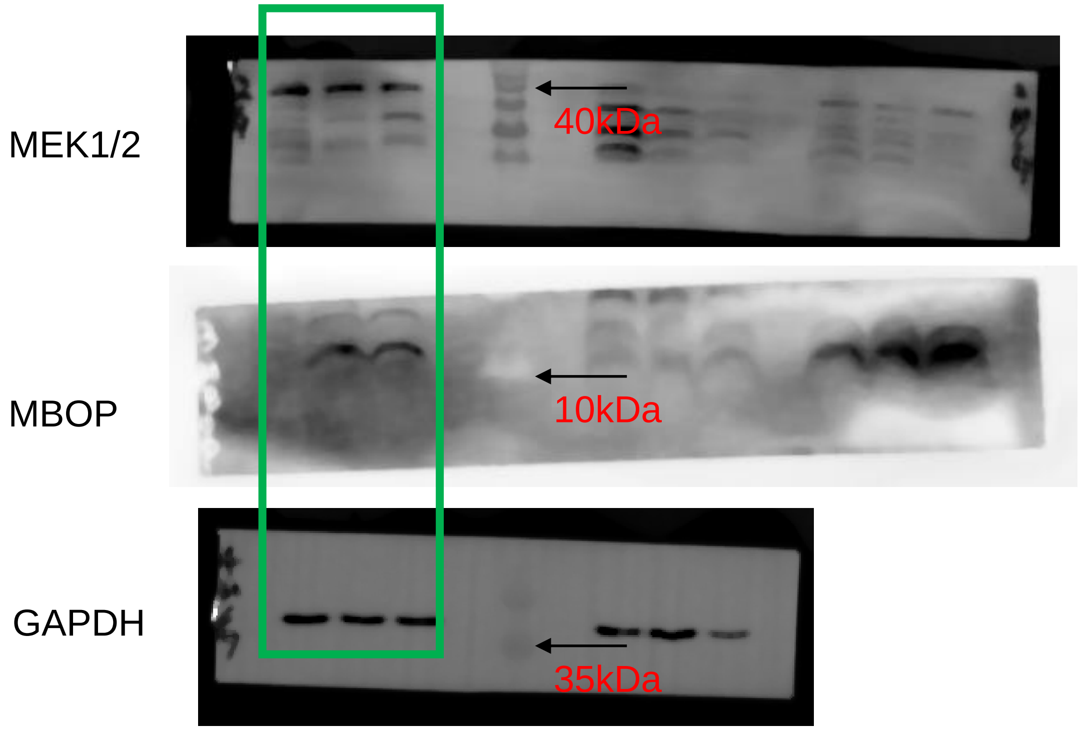

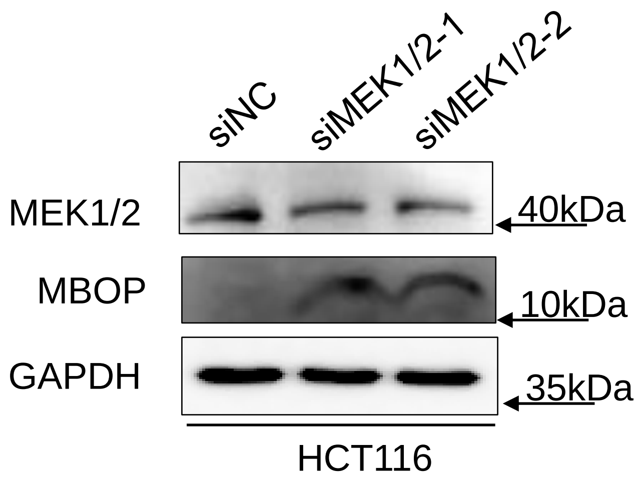

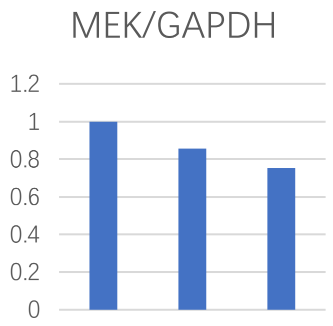

	siNC	siMAP2K1-1	siMAP2K1-1
MEK1/2	24458	19550	17016
MBOP	112174	137094	140588
GAPDH	29980	27962	27689
MEK/GAPDH	0.8158105	0.69916315	0.6145401
RATIO1	1	0.85701657	0.7532877
MBOP/GAPDH	3.7416278	4.90286818	5.0773954
RATIO2	1	1.31035702	1.3570017

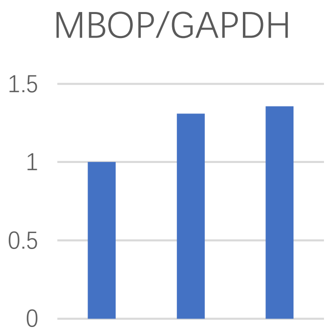

Fig. S4E

	pcDNA3.1+	pMEK-HA
MEK1/2	26006.000	55712.000
MBOP	231200	186415
GAPDH	19346.000	19169.000
MEK/GAPDH	1.3442572	2.90635923
RATIO1	1	2.16205589
MBOP/GAPDH	11.950791	9.72481611
RATIO2	1	0.81373829

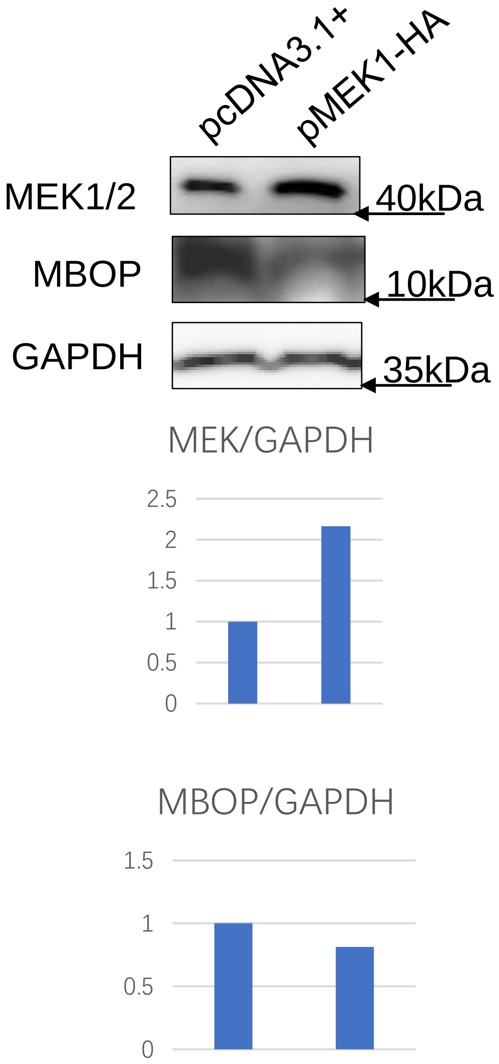

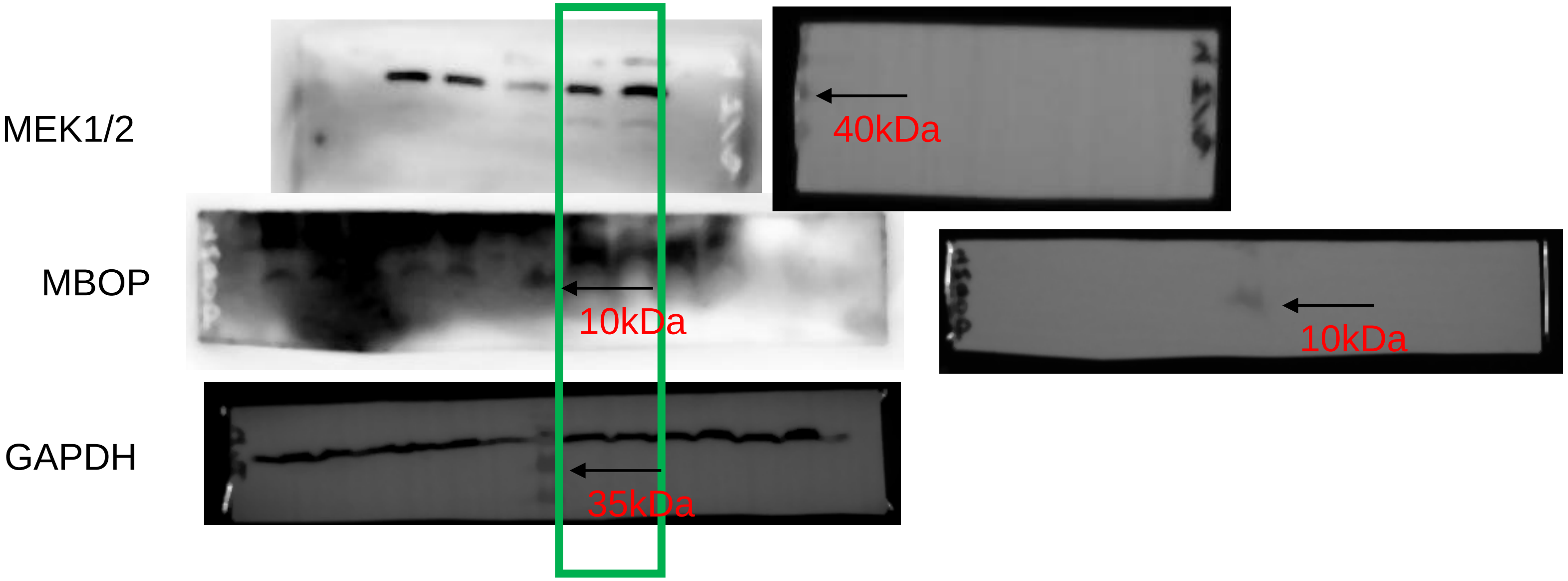

Fig. 6A

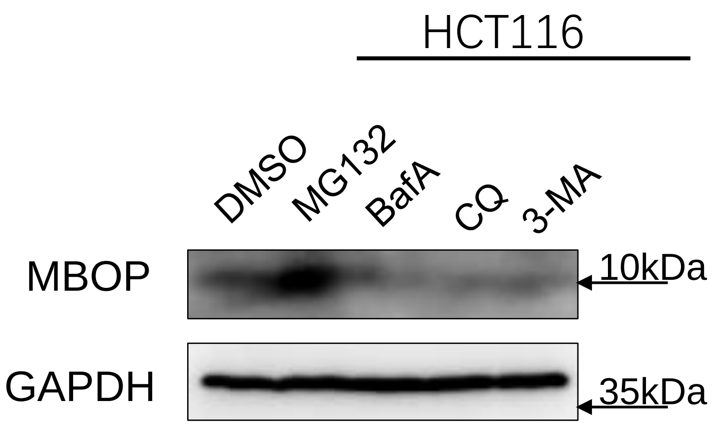

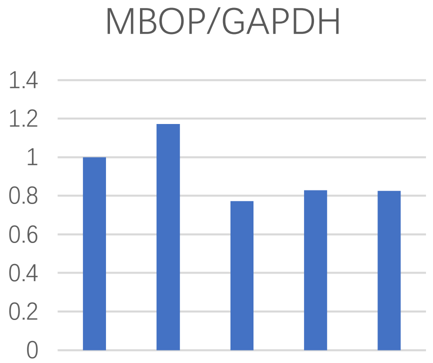

	DMSO	MG132	BafA	CQ	3-MA
MBOP	120685	168995	115016	106784	102569
GAPDH	65047	77705	80255	69456	66918
MBOP/GAPDH	1.855351	2.174828	1.433132	1.537434	1.532757
Ratio	1	1.172192	0.772432	0.828649	0.826128

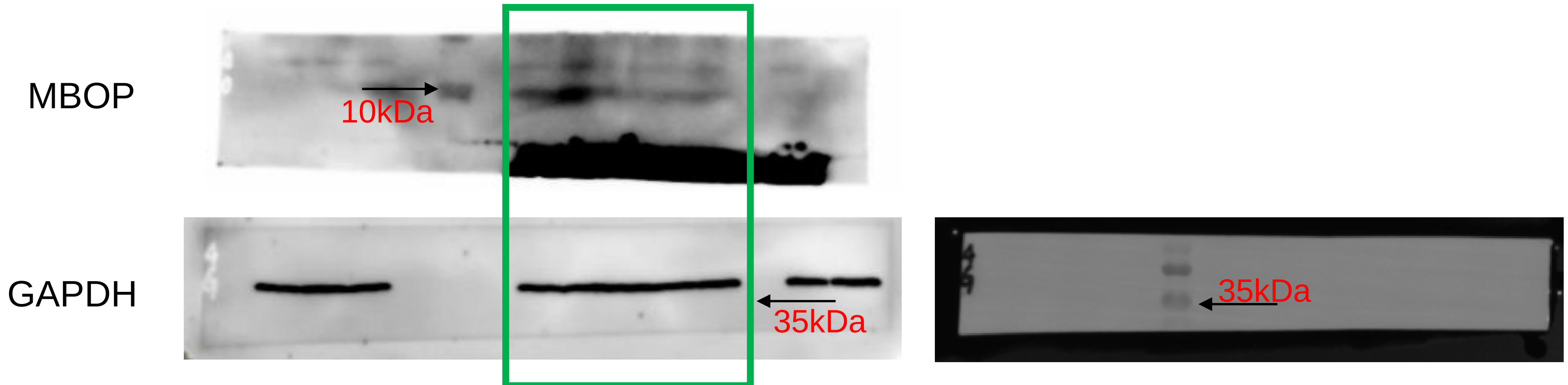

Fig. 6B

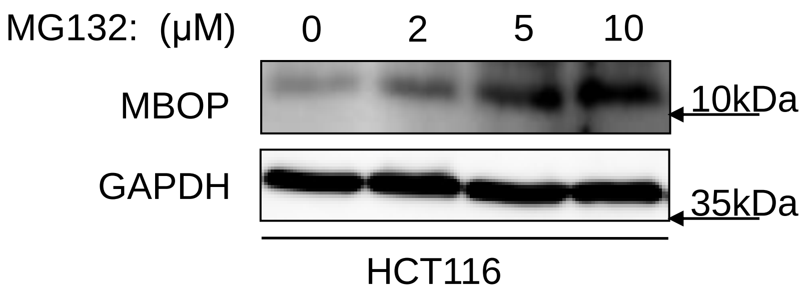

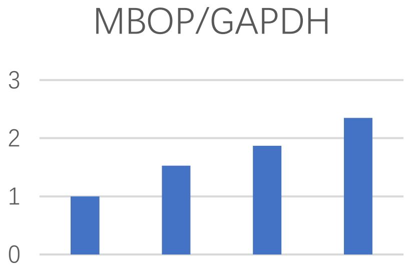

MG132(μM)	0	2	5	10
MBOP	89902	152457	182457	224910
GAPDH	109735	121709	119285	117002
MBOP/GAPDH	0.819265	1.252635	1.529589	1.922275
Ratio	1	1.528975	1.867027	2.346342

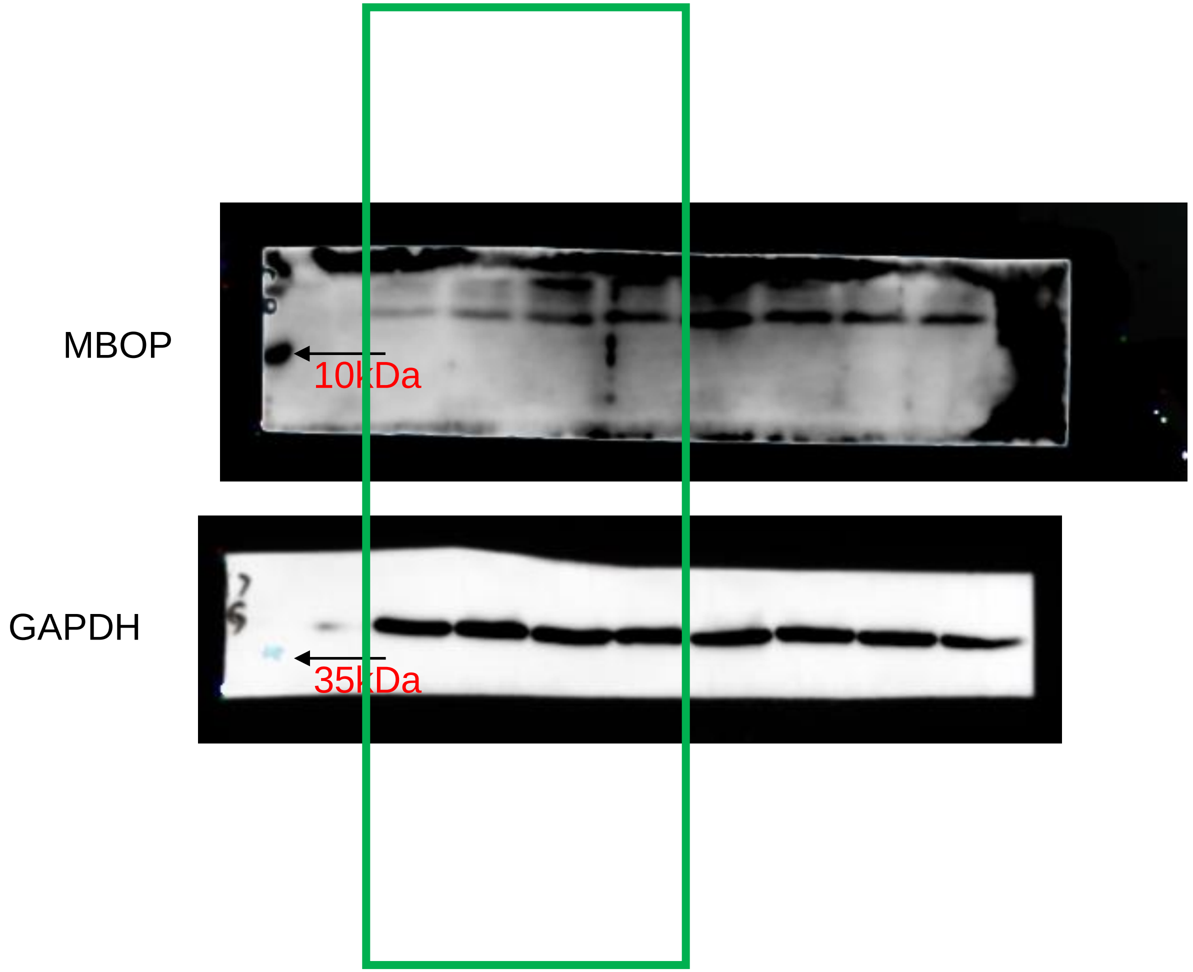

Fig. 6C right panel

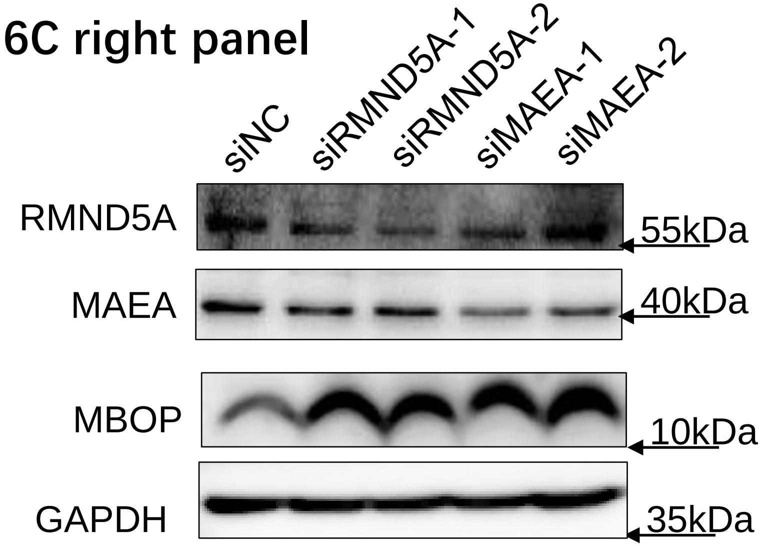

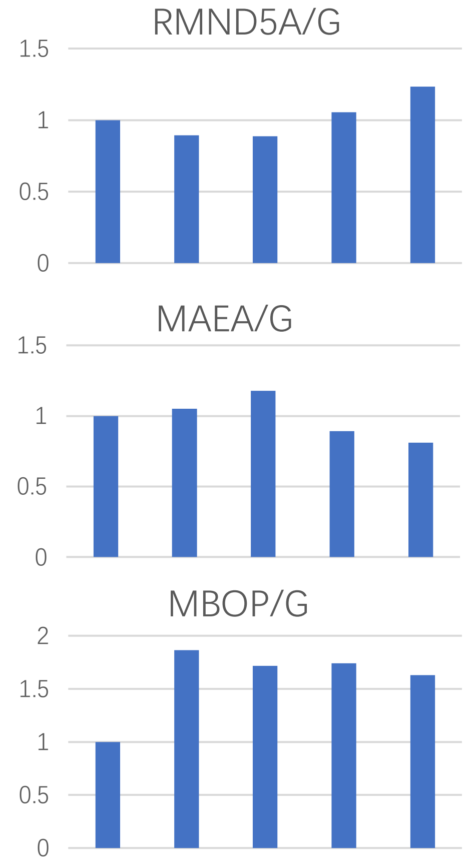

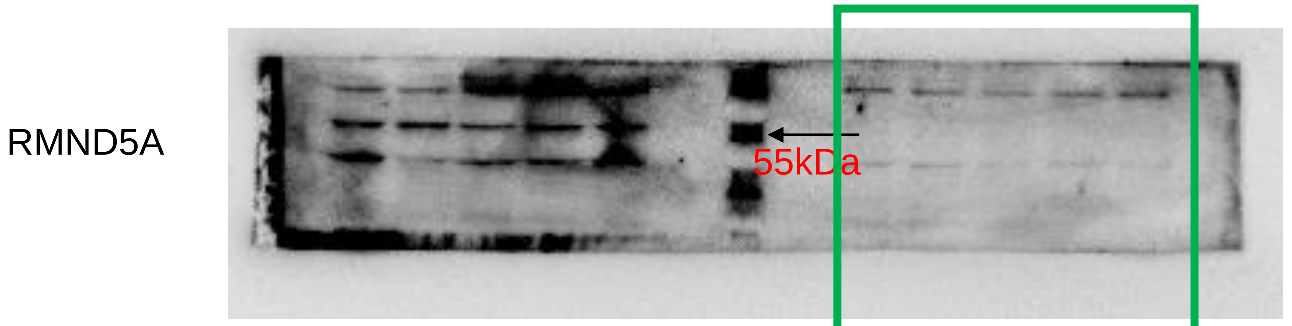

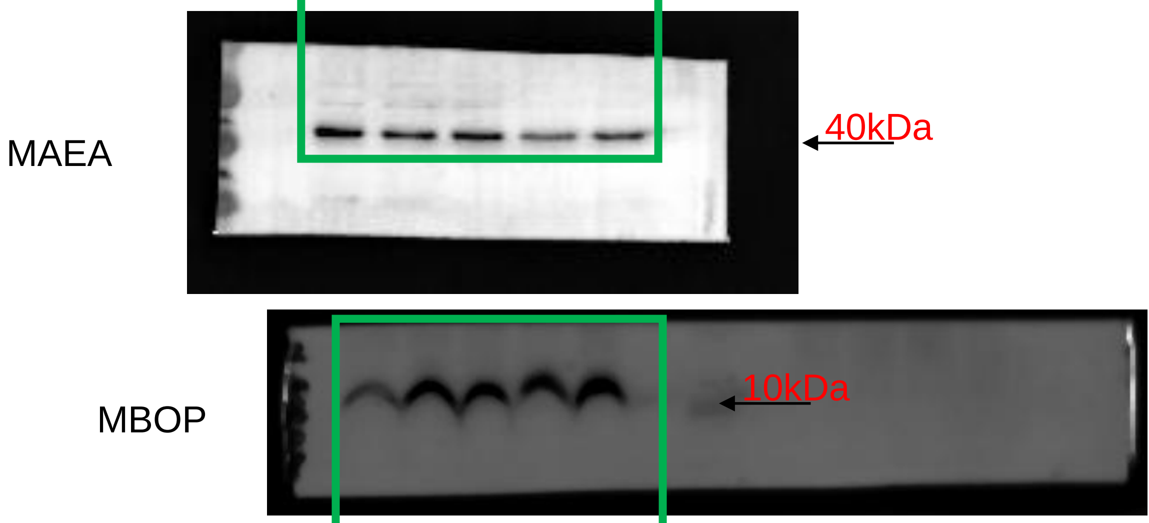

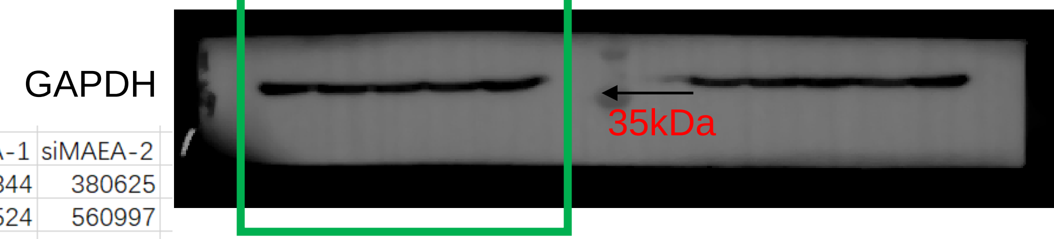

	siNC	siRMND5A	siRMND5A	siMAEA-1	siMAEA-2
RMND5A	296504	240512	234891	283844	380625
MAEA	665691	635141	700666	538524	560997
MBOP	85921	145151	131736	135478	145663
GAPDH	63803	57864	57040	57845	66350
RMND5A/GAPDH	4.64718	4.156505	4.118005	4.906976	5.736624
RATIO1	1	0.894415	0.88613	1.055904	1.234431
MAEA/GAPDH	10.43354	10.97644	12.28377	9.309776	8.455117
RATIO2	1	1.052035	1.177335	0.892293	0.810379
MBOP/GAPDH	1.346661	2.508485	2.309537	2.342087	2.195373
RATIO3	1	1.862745	1.71501	1.739181	1.630235

Fig. 6D

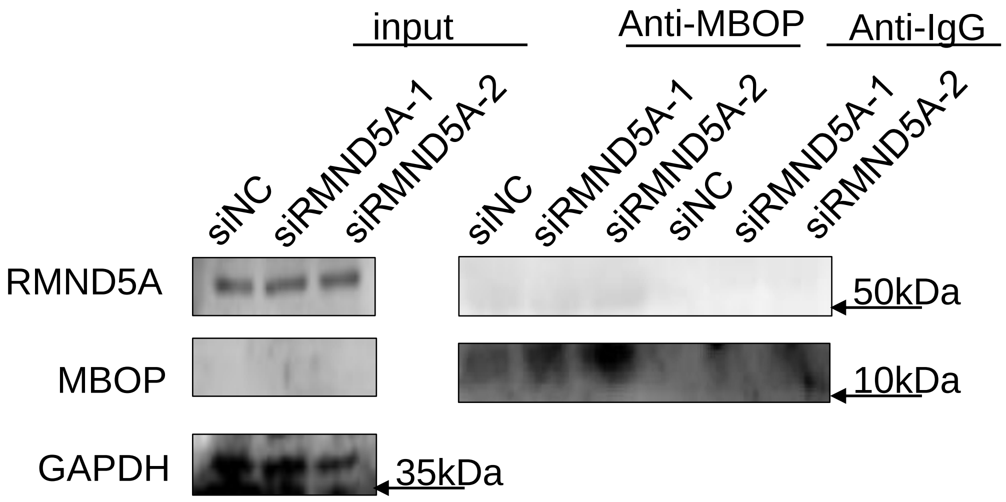

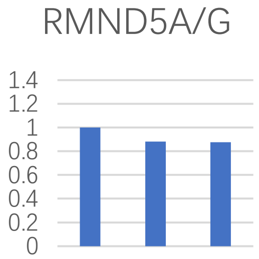

	siNC	siRMND5A	siRMND5A-2
RMND5A	47082	42810	39585
GAPDH	119894	123790	115203
RMND5A/G	0.392697	0.345828	0.343611
RATIO	1	0.880648	0.875003

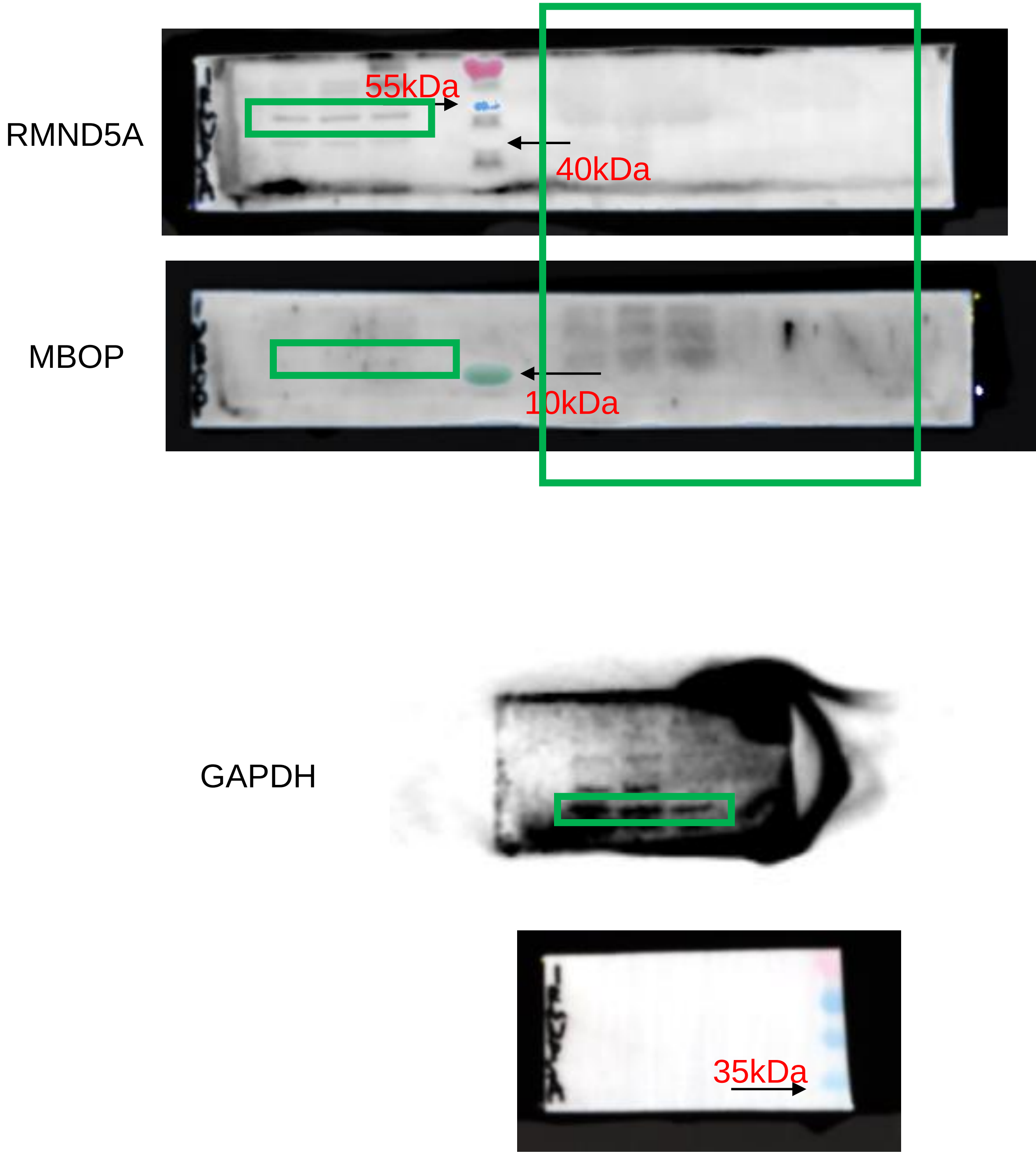

Fig. 6D

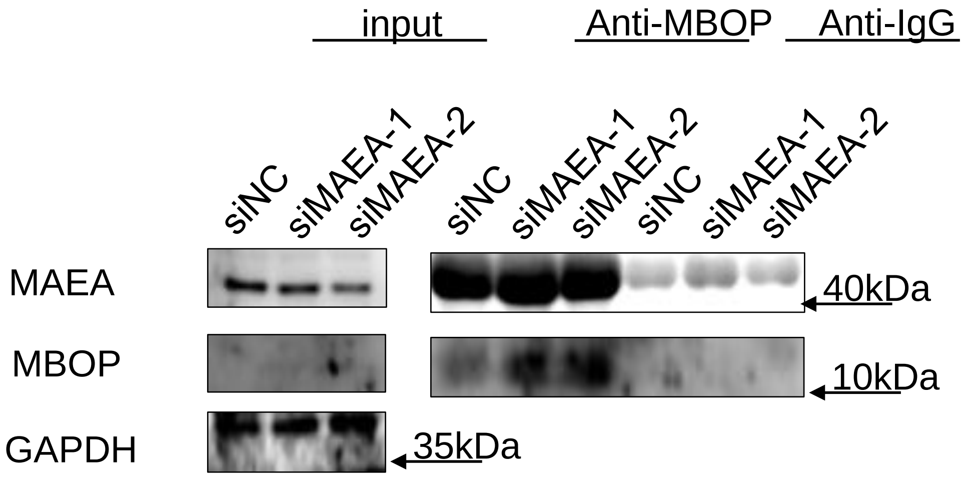

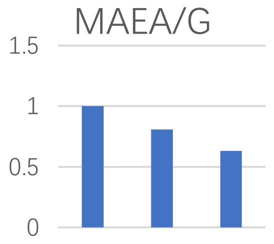

	siNC	siMAEA-1	siMAEA-2
MAEA	81853	61301	49913
GAPDH	164566	152450	159223
MAEA/G	0.497387	0.402106	0.313479
RATIO	1	0.808436	0.630251

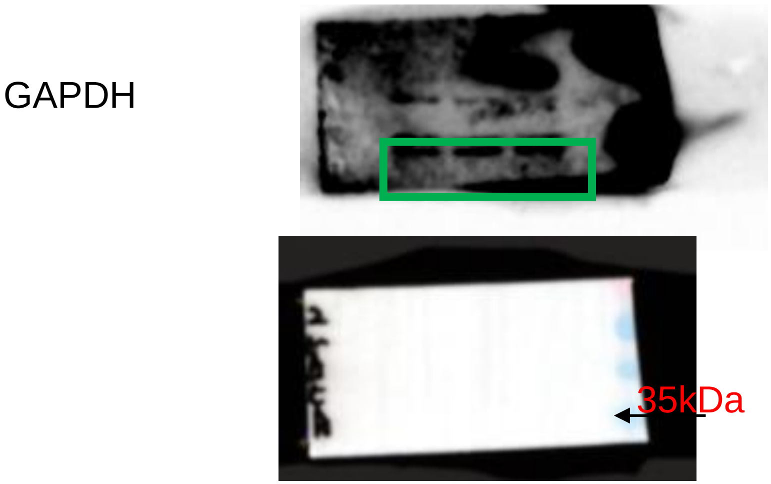

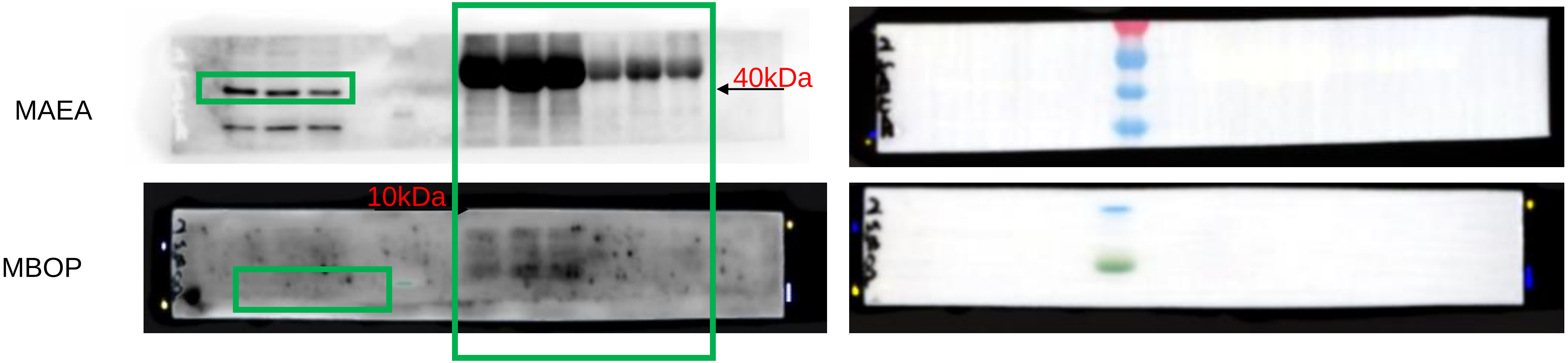

Supplement: Supplementary file 1 [file cancers-14-02338-s001.zip › File S1 Supplementary western blot results.pdf]
