# Supplementary material for: Oncopeptide MBOP Encoded by LINC01234 Promotes Colorectal Cancer through MAPK Signaling Pathway
Source: Cancers (Basel). 2022 May 9;14(9):2338. doi: 10.3390/cancers14092338 (PMC9100262; doi:10.3390/cancers14092338)
Supplement: Supplementary file 1 [file cancers-14-02338-s001.zip › Supplementary figures-MBOP.pdf]

Supplementary Figures

A

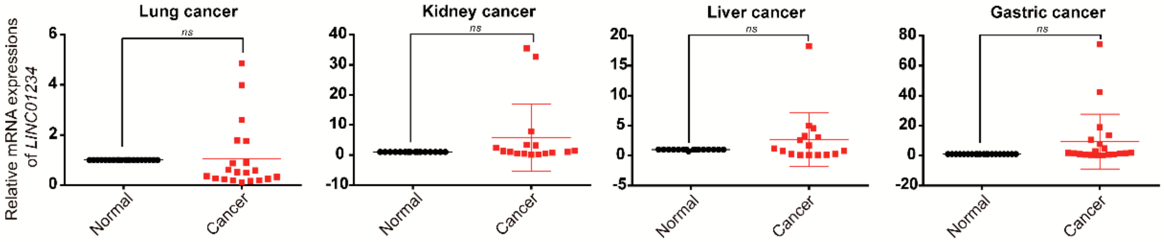

B

LncRNA	Acession Nubmer	Length	Reads in SW480	Reads in SW620
LINC01234	NR_110026.1	2222	1054	308
LINC01234	NR_110025.1	2088	294	29

Figure S1. *LINC01234* has ribosome binding sites across several perspectives (**A**). Expression of *LINC01234* in lung cancer (*n*=20), kidney cancer (*n*=16), liver cancer (*n*=16) and gastric cancer tissues (*n*=20), and data are represented as mean ± SD. (**B**). GSE139407 showed ribosome reads of *LINC01234* in colorectal cancer (CRC) cell lines SW480 and SW620.

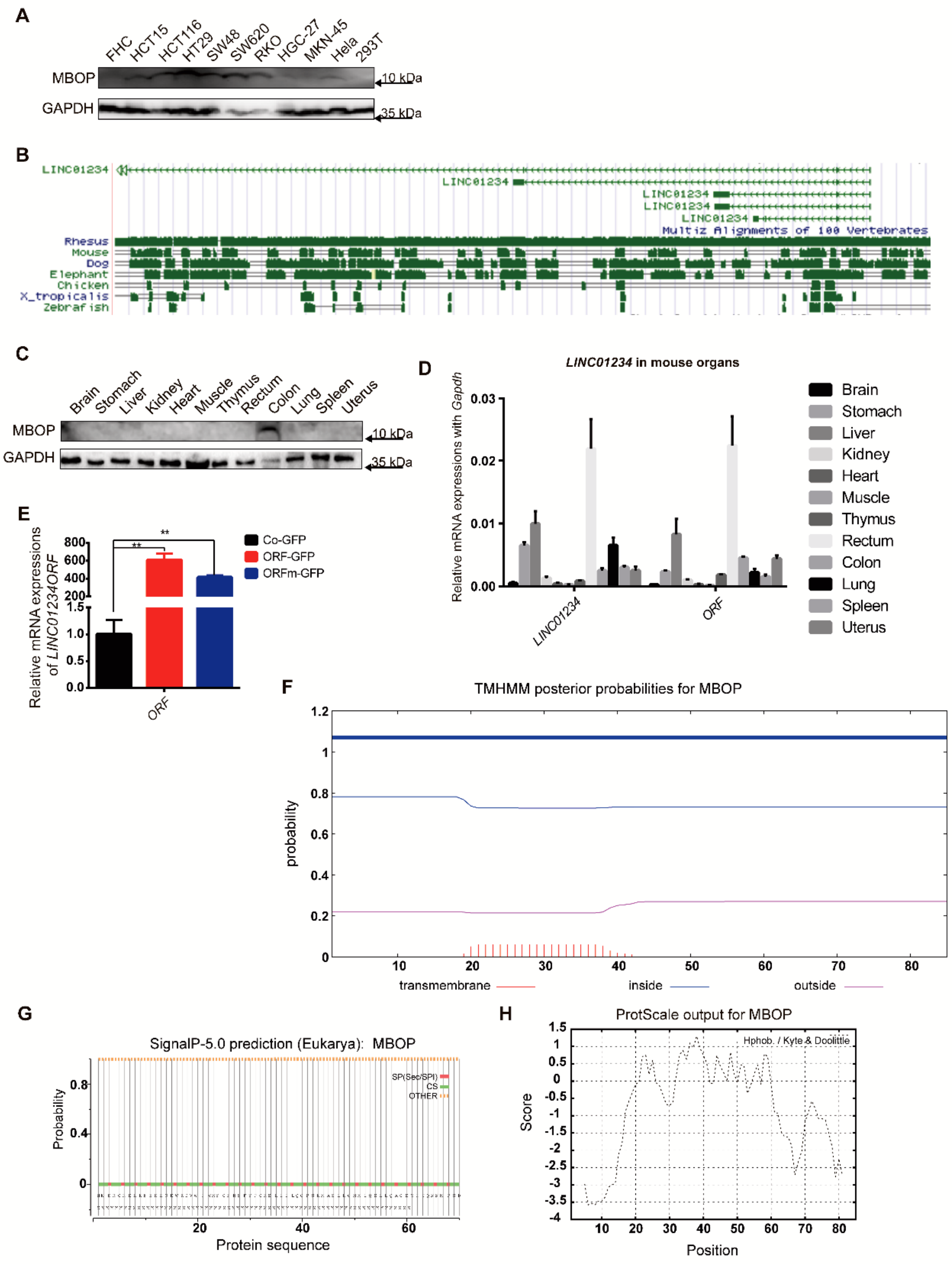

Figure S2. ***LINC01234* encodes an endogenous peptide highly expressed in CRC** (**A**). The expression of MBOP in various cell lines, including normal colonic epithelial cell line FHC, CRC cell lines HCT15, HCT116, HT29, SW48, SW620 and RKO, gastric cancer cell lines HGC-27 and MKN-45, cervical cancer cell line Hela, and embryonic kidney cell 293T. CRC cell lines showed higher expression of MBOP compared with other kinds of cell lines. (**B**). The conservation situation of *LINC01234ORF* in UCSC browser, and only rhesus monkeys shared similar sequence coverage with humans. (**C**). MBOP expressions in organs of a female BALB/c nude mouse, the colon showed a positive signal. (**D**). The mRNA expression of *LINC01234* and *LINC01234ORF* in organs of a female BALB/c nude mouse, data are represented as mean ± SD. (**E**). The expression situations of *LINC01234ORF* in lentiviral transfected cell lines HT29, data are represented as mean ± SD, **$p<0.005$. (**F**). Transmembrane

Hidden Markov Model server 2.0, (**G**). SignalP-5.0 server and (**H**). ProtScale all figured out there was no transmembrane region in MBOP.

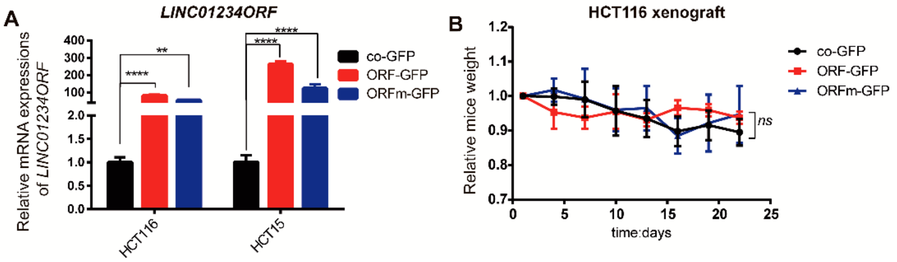

Figure S3. MBOP promotes CRC progression through cell migration and proliferation (**A**). The expression situations of *LINC01234ORF* in lentiviral transfected cell lines HCT116 and HCT15, data are represented as mean ± SD, **p<0.005, ****p<0.0001. (**B**). The relative mice weight of three groups, mice weight tended to decrease in all three groups, but no remarkable difference was observed.

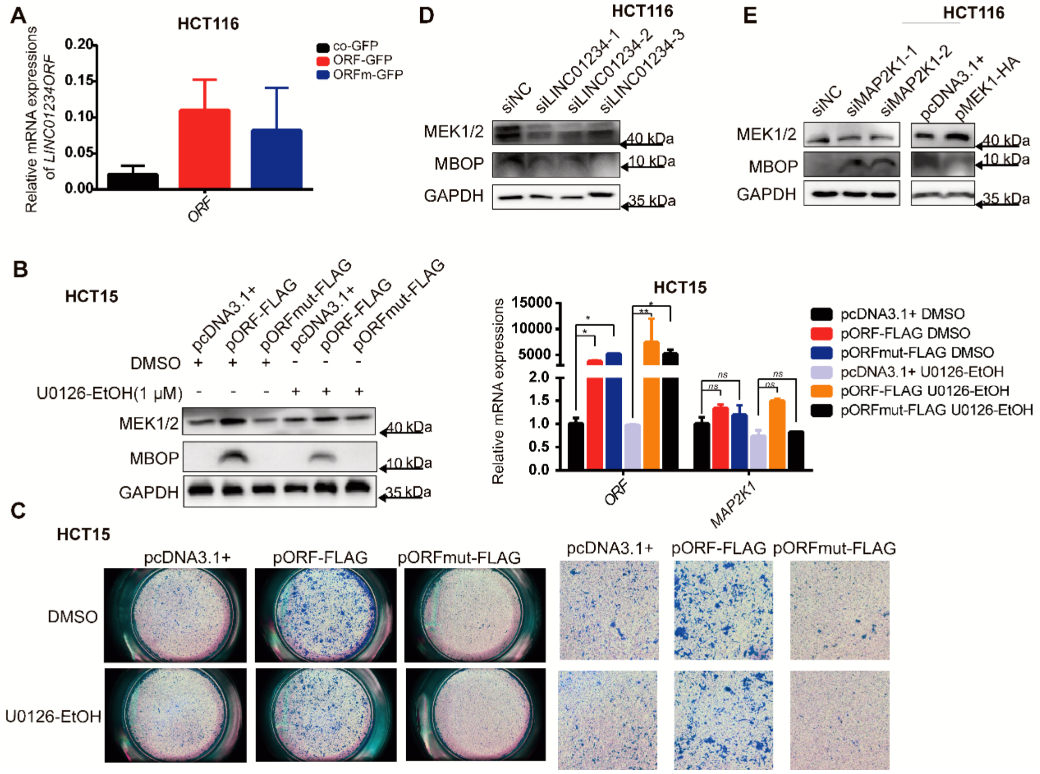

Figure S4. The MBOP/MEK1/pERK/MMP2/MMP9 axis in CRC (**A**) The relative expression of *LINC01234ORF* in tumors burdened by mice, which was represented as mean ± SD. (**B**). HCT15 cells were transfected with plasmids pcDNA3.1+, pORF-FLAG, and pORFmut-FLAG, followed by treatments of DMSO and U0126-EtOH. The overexpression of MEK1 and MBOP brought by transfecting cells with pORF-FLAG could be partially inhibited by U0126-EtOH, whereas the *MAP2K1* showed no significant alterations. Data are represented as mean ± SD, *p<0.05, **p<0.005. (**C**)The pro-migration effect driven by the transfection of pORF-FLAG could be partially reversed by U0126-EtOH.

(**D**). Knockdown of MBOP through siRNAs targeting *LINC01234* decreased the protein expression of MEK1. Note: Same siRNA-treated HCT116 samples from Fig. S4D were used to detect the expression of MBOP in Figure 2C. (**E**). Knockdown of MEK1 with siMAP2K1 increased the expression of MBOP while overexpression of MEK1 decreased the expression of MBOP.
